# Astrocyte regional specialization is shaped by postnatal development

**DOI:** 10.1101/2024.10.11.617802

**Published:** 2024-10-12

**Authors:** Margaret E. Schroeder, Dana M. McCormack, Lukas Metzner, Jinyoung Kang, Katelyn X. Li, Eunah Yu, Kirsten M. Levandowski, Heather Zaniewski, Qiangge Zhang, Edward S. Boyden, Fenna M. Krienen, Guoping Feng

**Affiliations:** 1. McGovern Institute for Brain Research, MIT, Cambridge, MA, USA.; 2. Department of Brain and Cognitive Sciences, MIT, Cambridge, MA, USA.; 3. Yang Tan Collective, MIT, Cambridge, MA, USA.; 4. The Broad Institute of MIT and Harvard, Cambridge, MA, USA.; 5. Center for Neurobiological Engineering and K. Lisa Yang Center for Bionics, MIT, Cambridge, MA, USA.; 6. Department of Biological Engineering, MIT, Cambridge, MA, USA.; 7. Koch Institute, MIT, Cambridge, MA, USA.; 8. Howard Hughes Medical Institute, Cambridge, MA, USA.; 9. Media Arts and Sciences, MIT, Cambridge, MA, USA.; 10. Princeton Neuroscience Institute, Princeton University, Princeton, NJ, USA

## Abstract

Astrocytes are an abundant class of glial cells with critical roles in neural circuit assembly and function. Though many studies have uncovered significant molecular distinctions between astrocytes from different brain regions, how this regionalization unfolds over development is not fully understood. We used single-nucleus RNA sequencing to characterize the molecular diversity of brain cells across six developmental stages and four brain regions in the mouse and marmoset brain. Our analysis of over 170,000 single astrocyte nuclei revealed striking regional heterogeneity among astrocytes, particularly between telencephalic and diencephalic regions, at all developmental time points surveyed in both species. At the stages sampled, most of the region patterning was private to astrocytes and not shared with neurons or other glial types. Though astrocytes were already regionally patterned in late embryonic stages, this region-specific astrocyte gene expression signature changed dramatically over postnatal development, and its composition suggests that regional astrocytes further specialize postnatally to support their local neuronal circuits. Comparing across species, we found divergence in the expression of astrocytic region- and age-differentially expressed genes and the timing of astrocyte maturation relative to birth between mouse and marmoset, as well as hundreds of species differentially expressed genes. Finally, we used expansion microscopy to show that astrocyte morphology is largely conserved across gray matter forebrain regions in the mouse, despite substantial molecular divergence.

## Introduction

The mammalian brain is composed of thousands of heterogeneous molecularly-defined cell types^1,2^. This heterogeneity is prominent between cells from different anatomical regions that arise from distinct developmental compartments. This regional specialization is critical for circuit formation and proper brain function. In recent years, this heterogeneity has been cataloged through large-scale single-cell and single-nucleus RNA sequencing (scRNAseq and snRNAseq, respectively), which enables molecular profiling in unprecedented detail and scale^3^. The past decade has seen the publication of multiple brain cell type transcriptomic atlases, including of the entire adult mouse brain^1,2^, the adult human brain^4^, the developing mouse^5^ and human^6^ brains, and the adult marmoset brain^7,8^. Most, but not all of these atlases have focused primarily on characterizing neurons, long considered the brain’s principal cell type. Indeed, several studies used cell sorting methods to enrich for neurons^9^.

Astrocytes, an abundant class of glia, play critical roles in neuronal circuit assembly and function in healthy and pathological states^10–14^. While their morphological heterogeneity has long been appreciated^15,16^, their molecular heterogeneity, particularly across brain regions, has been revealed more recently by bulk and single-cell RNA sequencing studies^8,17–21^ and earlier by microarray studies^22^. Early lineage tracing studies in the mouse spinal cord and brain revealed that astrocyte precursors from different embryonic domains are molecularly distinct^23,24^. Adult mouse astrocytes maintain epigenetic marks from their region-restricted radial glia ancestors^25^, which may contribute to the significant heterogeneity of adult astrocyte populations. There is also abundant evidence supporting the role of extrinsic cues in astrocyte regionalization, including the formation of various cortical morphological subtypes from a shared astrocyte progenitor^26^, the up- or down-regulation of ion channels, transporters, receptors in response to neuronal inputs^27^, and the molecular and morphological adaptation of distinct developmentally-patterned septal astrocyte subtypes after cross-region heterotopic transplant^28^.

As has been found with neurons, it is likely that transcriptionally-defined astrocyte populations are developmentally influenced by their respective microenvironments and have distinct functions in the healthy and diseased brain. Yet, the developmental time course of astrocyte regional patterning, the composition of astrocyte subtypes over development, and the conservation of these features between rodents and primates remain elusive^29^. To address this knowledge gap, we applied snRNAseq to characterize astrocyte molecular diversity across six developmental stages and four brain regions in mouse and marmoset. To complement the transcriptomic studies, we characterized complex astrocyte morphology and protein localization at high resolution across brain regions using expansion microscopy.

We used single nucleus sequencing to generate a dataset of 1.4 million brain cell nuclei across multiple stages and brain regions in mouse and marmoset. A unified study, with data generated from a single lab using highly consistent methodology, has the advantage of reduced technical variation compared to datasets integrated across research groups, nuclei isolation protocols, and sequencing platforms, which is difficult to remove *in silico*^30,31^.

Our analysis shows that astrocytes are regionally patterned before birth and at all subsequent time points. We found dramatic changes in the transcriptional signatures underlying astrocyte regional identity between birth and early adolescence in both species. We explored the functional implications of genes differentially expressed between astrocytes from different brain regions, and between astrocytes at different developmental time points, and we identified both region-shared and region-divergent developmental transcriptional signatures in astrocytes.

Many of the region-, age-, and species-differentially expressed genes in astrocytes were annotated in morphogenesis pathways. Indeed, astrocyte morphology, which is highly ramified and complex, including sub-micron scale processes that contact synapses and blood vessels, is essential for their many functions^32,33^. Therefore, to assess whether nanoscale astrocyte morphology is also regionally specialized, we used new variants of expansion microscopy, ExR^34^ and multiplexed ExR (multiExR), in combination with viral labeling of astrocytes to characterize astrocyte morphology and protein expression at the nanoscale. We found that gray matter astrocytes exhibit a high degree of morphological similarity across PFC, striatum, and thalamus, alongside differences in protein expression. Finally, we demonstrated proof of concept for brain-wide labeling and expansion of marmoset astrocytes via intravenous AAV injection and ExR.

## Results

### A multi-region transcriptomic atlas of the developing mouse and marmoset brain

**Figure 1.**
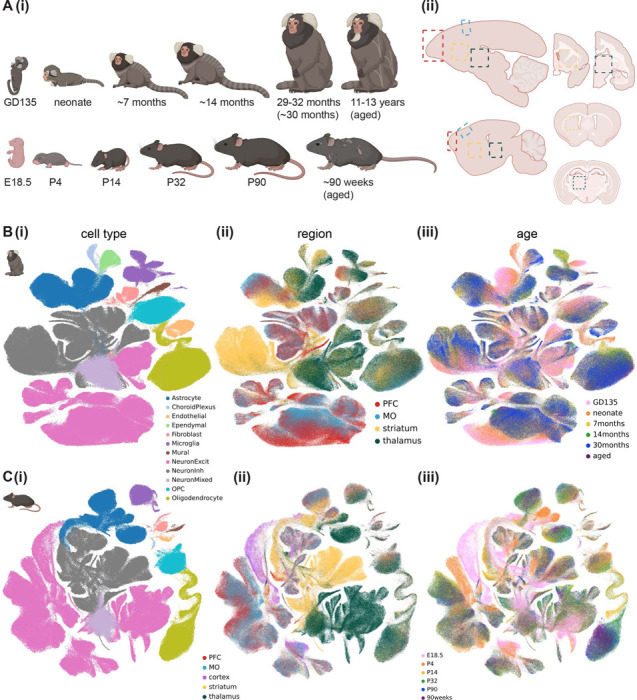
A multi-region transcriptomic atlas of brain cell diversity across postnatal development in marmoset and mouse. **A**, Cross-development, cross-region sampling strategy in marmoset (top row) and mouse (bottom row). **(i)** Developmental time points profiled (some approximate, see Methods and Table S1) GD, gestational day; E, embryonic day; P, postnatal day. **(ii)** Brain regions profiled, including prefrontal cortex (PFC, red dashed boxes), motor cortex (MO, blue dashed boxes), striatum (yellow dashed boxes), and thalamus (green dashed boxes), shown in either sagittal (left) or coronal slices (right, for subcortical regions only). Schematic generated using BioRender.com. **B-C**, Integrated UMAP embedding of marmoset (**B**, 881,832) or mouse (**C**, 597,668) nuclei from PFC, MO, striatum, and thalamus across all developmental time points assayed and a randomly downsampled portion of adult nuclei from our previous study^7^ colored by **(i)** assigned cell type **(ii)** dissected brain region, or **(iii)** developmental time point. Legend for **B-C(i)** is shared.

To create the cross-region, cross-species, cross-development snRNAseq atlas, we dissected prefrontal cortex (PFC), motor cortex (MO), striatum, and thalamus from freshly harvested mouse and marmoset brains at late embryonic, neonatal, early adolescent, late adolescent, young adult, and aged timepoints (Fig. 1A). We collected tissues from 2 marmoset donors, one male and one female, and from 3 mouse donors, at least one female, for each brain region and developmental time point except 90 weeks (Table S1). We generated single-nuclei suspensions from frozen tissue without enriching for any particular cell type, and generated single-nucleus transcriptomes using 10x Genomics Chromium v3.1 chemistry (see Fig. S1 for sequencing coverage statistics). Though the adult (4 donors aged 29–32 months, together labeled 30 months) marmoset snRNAseq data was generated using a different nuclei isolation protocol and reference genome^7^, the data integrated very well across studies (Fig. S2A). The data were also well integrated across biological sex (Fig. S2A-B).

After rigorous quality control, including removal of ambient RNA, low-quality nuclei, and doublets (see Methods), we obtained 597,668 mouse nuclei and 881,832 marmoset nuclei, which were composed of 12 broad cell classes (Fig. 1B(i), Fig. 1C(i); Fig. S2C-D). We annotated cell type (Leiden^35^-determined) clusters within each cell class in more detail (Fig. S3, Tables S2-4), using the Allen Brain Cell Atlas’s (ABCA) MapMyCells^36^ portal^36^ to refine our annotations of neuronal subtypes. The total neuron-to-astrocyte ratio (across regions and developmental time points) was slightly higher in mouse (6.28) than marmoset (5.24). Analysis of cluster distribution across regions revealed a modest amount of cross-region contamination (e.g. in marmoset, 4% of medium spiny neurons came from the thalamic dissection and 23% of thalamic excitatory neurons, which were mostly from GD135 and neonate time points, came from the striatal dissection), mostly in late embryonic and neonate samples. We corrected for this contamination post-hoc by reassigning the region annotation for contaminant nuclei based on the expression of *FOXG1* (a telencephalic marker), ABCA MapMyCells annotation, and/or subcluster identity (Fig. S4-5, see Methods). Throughout the paper, “dissected” brain region refers to the original region label for the sample in which the nucleus was processed, while “region” or “assigned region” refers to the brain region assigned post-hoc.

To more quantitatively assess cell type composition differences across dissected region (cortex, striatum, or thalamus), age, and sex, we used single-cell compositional data analysis (scCODA)^37^, which implements a Bayesian model of cell type counts to address the issue of low sample sizes in snRNAseq data. scCODA confirmed many significant differences in cell type proportion between regions and ages for each species, including the expected reduction in excitatory neurons in striatum and increasing oligodendrocyte abundance with age in both species, and minimal sex differences in cell type composition (Fig. S2E-F, Tables S5-8).

We found several cell type clusters that were enriched or depleted in developing (late embryonic or neonate) brains (Fig. S3C,G). For example, in both species, there were immature cortical excitatory neuron, microglia, and astrocyte clusters composed mostly of nuclei from late embryonic and fetal donors. The committed oligodendrocyte precursor (COP) and newly formed oligodendrocyte (NFOL) cluster was primarily composed of nuclei from the neonate time point in marmoset, with some nuclei even coming from late embryonic donors, but was primarily composed of nuclei from early adolescent (P14) donors in mouse, with no COP/NFOLs coming from E18.5 mouse.

### Astrocyte regional heterogeneity is embryonically patterned and unfolds over postnatal development

**Figure 2.**
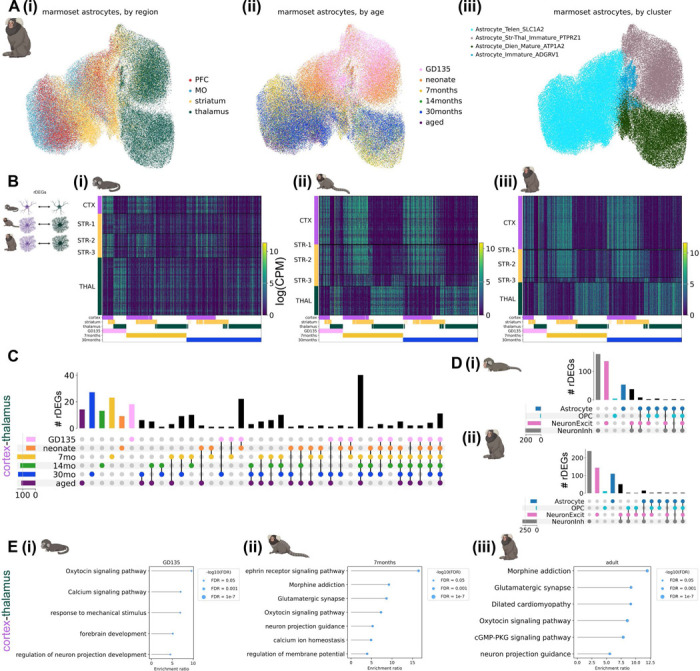
Developmental changes and cell-type specificity of astrocyte regional heterogeneity over postnatal development in the marmoset. **A**, Integrated UMAP embeddings of 103,009 marmoset astrocytes colored by **(i)** assigned brain region, **(ii)** developmental time point and **(iii)** Leiden cluster assignment. **B,** Expression heatmaps (rows are cells, columns are genes) of regional differentially expressed genes (rDEGs) between astrocytes from cortex, striatum, and thalamus at **(i)** GD135 and **(ii)** 7 month, and **(iii)** 30 month-old marmosets in log counts per million (logCPM). The raster plots beneath each heatmap indicate the time point (GD135, 7 months, or 30 months) and region(s) of upregulation (cortex, striatum, and/or thalamus) for each rDEG. Genes are ordered first by the time point at which they are an rDEG, then by the regions in which they are most highly expressed, and are plotted more than once if they are present at more than one time point. The same set of genes are plotted in the same order in **(i-iii)**. Striatal astrocytes are ordered by subtype identity (see Methods). **C**, UpSet plot showing the number of unique and overlapping cortex-thalamus rDEGs between developmental time points. The colored dots below each vertical bar indicate which age(s) share that set of rDEGs, while the colored horizontal bars indicate the total number of cortex-thalamus rDEGs for each age. **D**, UpSet plot (as in **(C)**) showing the number of overlapping cortex-thalamus rDEGs between OPCs (light blue), astrocytes (dark blue), excitatory neurons (pink), and inhibitory neurons (gray) for **(i)** neonate and **(ii)** adult marmoset. **E,** Gene ontology (GO) and pathway analysis on cortex-thalamus (enriched in either region) astrocyte rDEGs via WebGestalt 2024 in **(i)** GD135 **(ii)** 7 month and **(iii)** 30 month marmoset astrocytes. Lollipop plots show the enrichment ratio of GO Biological Process and KEGG pathways from an over-representation analysis with weighted set cover redundancy reduction, with tip size inversely proportional to the false discovery rate (FDR).

**Figure 3.**
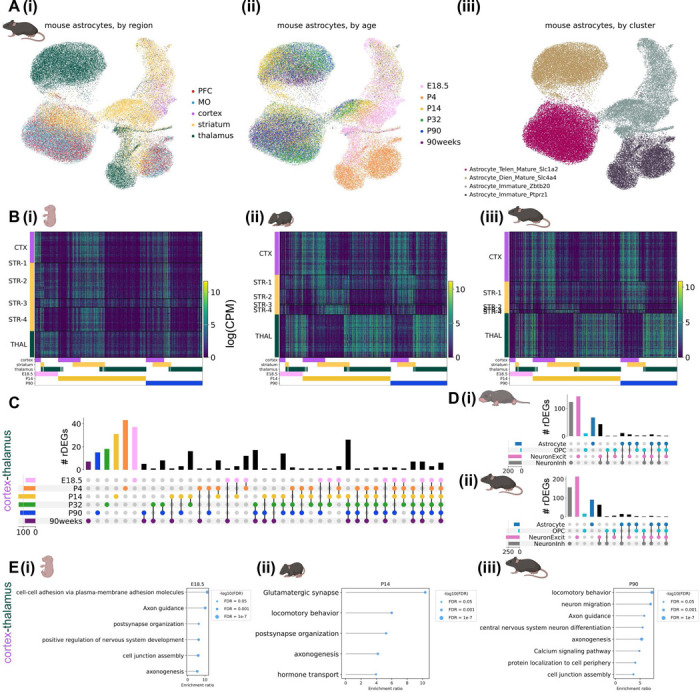
Developmental changes and cell-type specificity of astrocyte regional heterogeneity over postnatal development in the mouse. **A**, Integrated UMAP embeddings of 68,485 mouse astrocytes colored by **(i)** brain region **(ii)** developmental time point and **(iii)** Leiden cluster assignment. **B,** Expression heatmap (rows are cells, columns are genes) of regional differentially expressed genes (rDEGs) between astrocytes from cortex, striatum, and thalamus at **(i)** E18.5 **(ii)** P14, and **(iii)** P90 mice in log counts per million (logCPM). The raster plots beneath each heatmap indicate the time point (GD135, 7 months, or 30 months) and region(s) of upregulation (cortex, striatum, and/or thalamus) for each rDEG. Genes are ordered first by the time point at which they are an rDEG, then by the regions in which they are most highly expressed, and are plotted more than once if they are present at more than one time point. The same set of genes are plotted in the same order in **(i-iii)**. Striatal astrocytes are ordered by subtype identity (see Methods). **C**, UpSet plot showing the number of overlapping cortex-thalamus rDEGs between developmental time points. The colored dots below each vertical bar indicate which age(s) share that set of rDEGs, while the colored horizontal bars indicate the total number of cortex-thalamus rDEGs for each age. **D**, UpSet plot (as in **(C)**) showing the number of overlapping cortex-thalamus rDEGs between OPCs (light blue), astrocytes (dark blue), inhibitory neurons (gray), and excitatory neurons (pink) for **(i)** P4 and **(ii)** P90 mouse. **E,** Gene ontology (GO) and pathway analysis on cortex-thalamus (enriched in either region) astrocyte rDEGs via WebGestalt 2024 in **(i)** E18.5 **(ii)** P32 and **(iii)** P90 month mouse astrocytes. Lollipop plots show the enrichment ratio of GO Biological Process and KEGG pathways from an over-representation analysis with weighted set cover redundancy reduction, with tip size inversely proportional to the false discovery rate (FDR).

We observed striking regional heterogeneity among astrocytes at all developmental time points sampled in both species, particularly between astrocytes of diencephalic (thalamus) and telencephalic (cortex and striatum) origin (Fig. 2A, Fig. 3A). This is in line with multiple studies demonstrating embryonic regional patterning of astrocytes^6,8,24^. These regional populations further divided into an immature population, primarily composed of nuclei from late embryonic and neonatal time points, and a mature population, composed of nuclei from late adolescent timepoints onward. These separate populations suggest embryonically-patterned regional astrocyte populations undergo significant changes from the time of birth (neonate or P4) to early adolescence (7 months or P14). Notably, abundant populations of immature astrocytes remained present in the mouse striatum through adulthood (P90).

Transcription factors and morphogen gradients set up initial boundaries between developmental compartments such as the telencephalon and diencephalon^38,39^. It could be that such early influences are present only transiently at initial astrocyte specification, or it could be that later stages retain initial molecular distinctions and accumulate others over development. We calculated regional differentially expressed genes (rDEGs) at each developmental time point from metacells, 1-dimensional vectors of averaged normalized expression across all cells in a given grouping (see Methods), of each region. For marmoset, where each biological replicate donated 2 samples from each brain region, we required that rDEGs be above threshold requirements in both donors. We found 70 rDEGs whose expression differed between fetal cortical and thalamic astrocytes, 142 of such rDEGs by early adolescence (7 months), and 134 in adulthood (29–32 months, see Fig. 2B(i-iii) for the expression pattern of the union of these rDEGs at each timepoint). Focusing on cortex-thalamus rDEGs (which were most numerous, Table S9), we found that relatively few persisted across all developmental timepoints (Fig. 2C). 50% were shared between fetal and neonate, and 51% between late adolescent and aged, but only 4% continued to act as regional patterning signatures throughout the lifespan. We found that many more rDEGs were shared between late adolescent, young adult, and aged time points (61) than between fetal, neonate, and 7-month time points (23) and neonate, 7-month, and 14-month time points (25, Fig. 2C).

If early telencephalic and diencephalic patterning persists in astrocytes, cortex and striatum should retain common rDEGs compared to thalamus. To assess the degree of pairwise astrocyte rDEGs sharing across the 3 brain structures, we correlated the log fold-change difference in astrocyte regional gene expression between different region pairs (e.g., cortex-striatum vs. cortex-thalamus) for both rDEGs (log fold-change > 0.5) and non-rDEGs at fetal, early adolescent, and adult timepoints. We found that cortex-striatum vs. cortex-thalamus fold-changes exhibited a high degree of correlation in fetal marmoset (Pearson’s r = 0.78), which decreased over developmental time (Pearson’s r = 0.47 in adult marmoset, Fig. S6A-C(i)). At all 3 time points, striatum-thalamus vs. striatum-cortex fold-changes were negatively or weakly correlated (r = −0.43, −0.26, and 0.04 at GD135, 7 months, and 30 months respectively, Fig. S6A-C(ii)). Finally, thalamus-striatum vs. thalamus-cortex fold-changes were highly correlated (r > 0.80) at all 3 timepoints (Fig. S6A-C(iii)). Together, these results suggest that cortical and striatal astrocytes share transcriptional divergence from thalamic astrocytes at all ages, but become more transcriptionally similar later in development.

Dorsal radial glia populate the neocortex in a stereotyped progression, giving rise first to glutamatergic neurons, then to astrocytes, and finally to oligodendrocytes^40–42^. In the developing thalamus, radial glial progenitors likely follow the same cell type sequence^41,43^. As we showed previously for variable genes across the adult neocortex^7^, far more adult cortex-thalamus rDEGs are private to astrocytes than are shared with neurons or OPCs, despite their shared lineage^42^ (Fig. 2D). Surprisingly, this remained true even at the earliest stages we sampled (GD135 and neonate). Together these observations suggest that astrocytes gain regional identity early in their maturation, but that their continued regional identity is facilitated by distinct genes across their lifespan (Fig. 2C).

Many rDEGs nominate core cellular functions that may be further regionally specialized in astrocytes. For example, ephrins such as *EFNB2* and *EFNA5* are up-regulated in cortical astrocytes and ephrin receptor *EPHB1* is upregulated in cortical and striatal astrocytes. Neuron-astrocyte signaling via ephrin ligands and receptors regulates axon guidance and synaptogenesis^44^. Cyclic-AMP-related signaling molecules *ADCY1* and *ADCY8* are upregulated in thalamic astrocytes. As in neurons, astrocytic cAMP is an important second messenger following GPCR activation^45^, and modulates synaptic plasticity^46^. *ITPR1*, a calcium channel that controls calcium release from the endoplasmic reticulum, an important source of intracellular calcium during astrocyte signaling^47^, is also upregulated in thalamic astrocytes. Additionally, astrocyte rDEGs included ion channels (e.g. *TRPM3*, a non-selective Ca^2+^ permeable ion channel and thalamic rDEG), synapse-related proteins (e.g., *SPARC* in thalamic astrocytes, which regulates synaptogenesis^48^), neurotransmitter receptors (e.g., *SLC6A11* or GAT3, a thalamic rDEG and GABA transporter, and *GRM3* or mGluR3, a telencephalic rDEG and metabotropic glutamate receptor), and a thyroid hormone receptor (*SLCO1C1*, a cortical rDEG).

To characterize astrocyte rDEG pathways in a more unbiased manner, we used WebGestalt 2024^49,50^ over-representation analysis to test for enrichment of cortex-thalamus rDEGs (bidirectionally, i.e. upregulated either in cortex or thalamus) in GO Biological Process and KEGG pathways. Enriched pathways implicated oxytocin and calcium signaling and neuronal projection development for GD135 astrocyte rDEGs; ephrin signaling, synaptic transmission, and calcium ion homeostasis for 7-month astrocyte rDEGs; and glutamatergic synaptic transmission, oxytocin signaling, and cGMP-PKG signaling for adult marmoset astrocyte rDEGs (Fig. 2E). A summary of WebGestalt results for cortex-thalamus astrocyte rDEGs each age is provided in Table S10. Together, these results suggest that astrocytes are regionally specialized with the physiological adaptations necessary to support neuronal transmission and activity in their local environment.

As with marmoset, mouse astrocyte gene expression varied across developmental timepoints and most astrocyte rDEGs were not shared with other cell types (Fig. 3A-D, Table S11). E18.5 astrocyte cortex-thalamus rDEGs (79 total) included *Cacna2d1*, *Cntn5*, *Nrxn1*, *Creb5*, *Slco1c1*, and *Slc6a11*, and together were enriched for biological processes including cell-cell adhesion, axon guidance, and postsynaptic organization (Fig. 3E(i), Table S12). P14 astrocyte cortex-thalamus rDEGs included 26 of the rDEGs present at E18.5 (19% of total P14 rDEGs), in addition to rDEGs that only emerged at P14 including voltage-gated calcium channel subunit *Cacna1a*, the glutamate-gated kainate receptor *Grik4*, the N-glycoprotein *Thsd7a*, the cholesterol transporter *Gramd1b*, and the inward-rectifying potassium channel *Kcnj6*. Together, P14 cortex-thalamus astrocyte rDEGs were enriched in glutamatergic synapse, hormone transport, and postsynaptic organization pathways (Fig. 3E(ii), Table S12). There were 124 cortex-thalamus astrocyte rDEGs at P90, which included many of the rDEGs present at earlier time points (17% of P90 rDEGs were present at E18.5 and 52% were present at P14), and were enriched in neuron migration, axon guidance, calcium signaling, and cell junction pathways (Fig. 3dE(iii), Table S12).

As we did with marmoset, we assessed in mouse the degree to which regions shared pairwise astrocyte rDEGs by correlating log-fold change gene expression differences between region pairs at E18.5, P14, and P90. We found that cortex-striatum vs. cortex-thalamus fold-changes exhibited a high degree of correlation at E18.5 (Pearson’s r = 0.85), which decreased dramatically at P14 (r = 0.26) and increased again at P90 (r = 0.40, Fig. S6D-F(i)). Compared to marmoset, striatum-cortex vs. striatum-thalamus rDEGs were more positively correlated at P14 (r = 0.48) and P90 (r = 0.19, Fig. S6D-F(ii)). Consistent with marmoset, thalamus-striatum vs. thalamus-cortex fold-changes were highly correlated (r > 0.70) at all 3 timepoints (Fig. S6D-F(iii)). Together, these results suggest that while cortical and striatal astrocytes share transcriptional divergence from thalamic astrocytes, likely reflecting the their distinct telencephalic-diencephalic origins, mouse striatal astrocytes develop and maintain a unique transcriptional signature distinct from cortex and thalamus.

To validate the existence of these regional astrocyte populations and the differential expression of selected rDEGs *in situ*, we conducted multiplexed RNA fluorescence *in situ* hybridization (FISH) using the RNAscope HiPlex technology (Advanced Cell Diagnostics) in neonate and adult animals of both species. We used CellProfiler 4.2.5^51,52^ to quantify the fraction of astrocytes positive for each target gene in each region and the fraction of each astrocyte nuclei covered by the probe for each target gene in each region (see Methods). Most rDEGs followed the expected regional and developmental expression pattern in marmoset astrocytes, including *SPARC*, which was enriched in diencephalic astrocytes, *FOXG1*, which marked telencephalic astrocytes, *GFAP,* which was elevated in thalamus in adult but not neonate, and *KCNH7*, which was a telencephalic rDEG in neonate but not adult (Fig. S7-8, see Methods and Supplementary Note 1). Similarly, we found that most mouse astrocyte rDEGs followed the expected regional and developmental expression pattern in P4 and P90 mouse astrocytes, including *Clmn, Slco1c1, Csmd1*, and *Kcnd2* (Figs. S9-10, see Table S13 for source data and statistics).

To assess the extent of astrocyte intra-regional heterogeneity, we performed subclustering on cortical, striatal, and thalamic astrocytes from all developmental time points separately for each species (Fig. S11, see Methods). We found at least 4 astrocyte subclusters within each region, which primarily distinguished protoplasmic and fibrous/interlaminar subtypes (the latter being identified by *GFAP*, *AQP4*, and/or *ID3* expression^53^) and immature and mature astrocytes. In both species, the majority of astrocytes in the cortex and striatum were protoplasmic. In the marmoset thalamus, a larger proportion of astrocytes were *GFAP*+, *AQP4*+, or *ID3*+ (Fig. S11C), suggesting a higher proportion of fibrous astrocytes, consistent with the greater abundance of white matter in this region (Fig. S2C(i)). Nevertheless, it is unclear the extent to which the definitions of protoplasmic, fibrous, and intralaminar apply outside of the cortex. The mouse striatum had the most intra-regional heterogeneity, with 16 subclusters (Fig. S11E), in large part due to immature populations including *Top2a*+ rostral migratory stream progenitors. As in previous studies, we found that *CRYM* marks a subset of striatal astrocytes^54^ and *SPARC* marks thalamic astrocytes in both species^21^. Taken together with our FISH data, which was obtained in gray matter regions, our subclustering analysis suggests that most of the astrocytes in our study, and therefore likely most of the resulting rDEGs, arise from protoplasmic or gray matter astrocytes.

### Shared and subtype-specific predicted mechanisms of neuron-astrocyte communication

**Figure 4.**
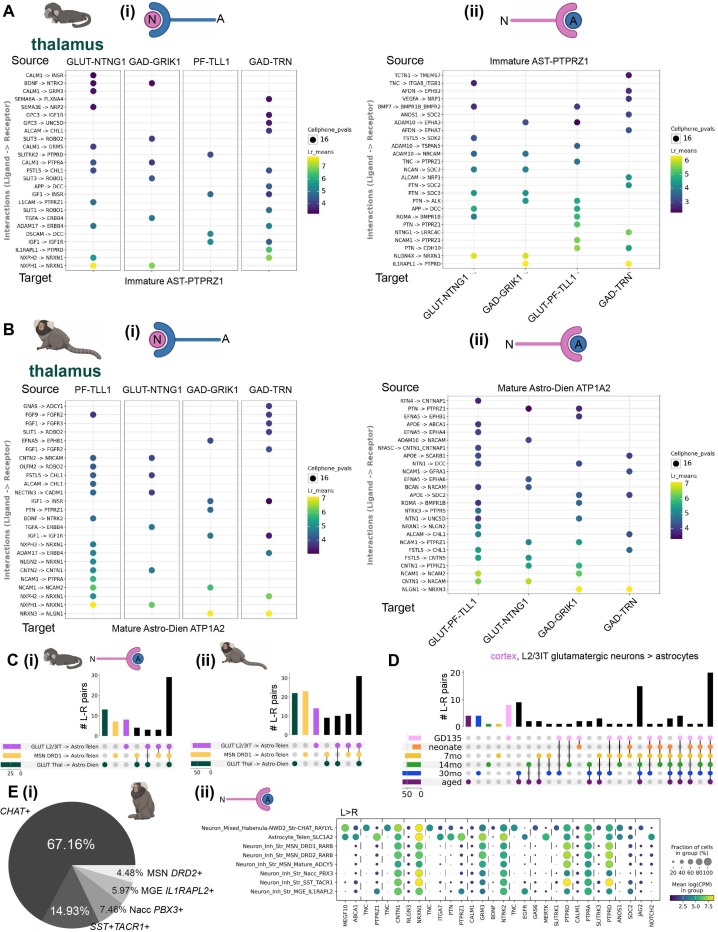
Cell-cell communication analysis for neuron-astrocyte and astrocyte-neuron predicted ligand-receptor pairs across regions and developmental time points in marmoset. **A,** Dot plot showing magnitude and specificity of the top 25 near-unique (shared with at most one other neuronal cluster) CellPhoneDB-predicted **(i)** neuron-astrocyte and **(ii)** astrocyte-neuron ligand receptor pairs for the most abundant astrocyte and neuronal Leiden clusters in the fetal marmoset thalamus. The source cell (top of the plot) expresses the ligand (left side of arrow on the row labels), while the target cell (bottom of the plot) expresses the receptor (right side of arrow on the row labels). The color of the dot indicates ligand-receptor expression magnitude (“Lr_means”, calculated as the average of the mean expression of the ligand in the source group and the mean expression of the receptor in the target group), while the size of the dot is inversely related to the p-value on ligand-receptor expression sensitivity (-log10(p)). **B,** Same as **(A)**, for the 14-month marmoset thalamus. **C,** UpSet plot showing the number of overlapping neuron-astrocyte predicted ligand-receptor pairs between regions, from the most abundant neuronal and astrocyte subtypes in each region for **(i)** fetal and **(ii)** late adolescent marmoset. For cortex (purple), glutamatergic L2/3IT neurons to cortical astrocytes; striatum (yellow), *DRD1*+ medium spiny neurons to striatal telencephalic astrocytes; and thalamus (green) thalamic glutamatergic neurons to thalamic astrocytes. The colored dots below each vertical bar indicate which regional neuron-astrocyte (N-A) subtypes share that set of L-R pairs, while the colored horizontal bars indicate the total number of L-R pairs for each N-A subtype. **D**, UpSet plot (as in **(C)**) showing the number of overlapping cortical glutamatergic L2/3IT neuron to cortical astrocyte predicted ligand-receptor pairs between ages. **E,** Examination of unique predicted ligand-receptor interactions between individual striatal neuron Leiden clusters and striatal astrocytes. **i,** Pie chart showing the proportion of unique striatal neuron to striatal astrocyte predicted ligand-receptor pairs for each Leiden cluster of striatal neurons in the adult marmoset. *CHAT*+, cholinergic interneurons (ChINs); Nacc, nucleus accumbens; MSN, medium spiny neurons, MGE, medial ganglionic eminence. **ii,** Dotplot showing expression of selected unique (not significantly enriched in other striatal neurons) ChIN-astrocyte ligand-receptor pairs in ChINs (top row), striatal astrocytes (second row), and other striatal neurons (bottom 6 rows) in the adult marmoset. Ligands are odd gene names, starting from *MEGF10* and receptors are the even gene names on their right, starting with *ABCA1*. The full list of unique ChIN-astrocyte predicted ligand-receptor pairs and their associated mean expression and CellPhoneDB p-values are provided in Table S15.

Many of the astrocyte rDEGs implicated neuron-astrocyte communication, suggesting that the regional molecular identity of astrocytes may arise in part from customized interactions with the vast diversity of specialized neuronal types across the mammalian brain^1,4,55^. Our previous analysis showed that rDEGs are not substantially shared across neurons and glia (Figs. 2–3D), which rules out the influence of pan-cell type regional patterning. Neurons and astrocytes communicate via myriad signaling pathways. We assessed whether neuron and astrocyte cell type pairs sampled from the same region were over-enriched for known ligand-receptor (L-R) interactions. Using CellPhoneDB, which calculates the magnitude and specificity of expression of annotated L-R pairs across pairs of cell types. To increase the specificity of our predicted L-R results, we restricted CellPhoneDB analysis to neurons and astrocytes only (see Methods). Across most brain regions and ages in the marmoset, we found neurexin and neuroligin (NRXN/NLGN) family members, contactin (CNTN) family members, fibroblast growth factor and receptor (FGF/FGFR) family members, and neural cell adhesion molecule (NCAM) family members to be the most enriched predicted neuron-astrocyte and astrocyte-neuron L-R genes (Table S14). Despite the commonality of these L-R pairs between astrocytes and all neuronal subtypes, for each neuronal subtype in a given region, we found unique or near unique L-R and R-L pairs with astrocytes. For example, in the fetal marmoset thalamus, *SLT3*→*ROBO2* is specific to parafascicular/immature *TLL1*+ neurons and immature thalamic astrocytes, while *AFDN*→*EPHA7* signaling is specific to immature astrocytes and TRN GABAergic neurons, compared to the other neuronal subtypes examined (Fig. 4A). Later in development, at 14 months, many of the same neuron-astrocyte and astrocyte-neuron L-R combinations were present, while some new pairs, such as *EFNA5*→*EPHB1* for thalamic astrocytes to *GRIK1*+ midbrain-derived GABAergic neurons, emerged (Fig. 4B).

To summarize the shared and divergent expression of predicted L-R pairs underlying neuron-astrocyte communication across regions, we examined the overlap of these pairs for the most abundant neuronal cluster and the most abundant astrocyte cluster (presumed predominantly protoplasmic) in cortex, striatum, and thalamus (Fig. 4C). We found that many L-R pairs were shared across regions at both GD135 and 14 months (43% and 29% of total L-R pairs, respectively), while the thalamus (at GD135), and later striatum (at 14 months) had the most L-R pairs not shared with other regions for the clusters examined. Several region-specific neuron-astrocyte/astrocyte-neuron L-R pairs were in the same family as those present in other regions. For example, in 14-month marmoset, *EFNA5*→*EPHA5* was unique to cortical astrocytes → cortical L2/3IT glutamatergic neurons, while *EFNA5*→*EPHA7* was unique to striatal astrocytes → *DRD1*+ medium spiny neurons, and *EFNA5*→*EPHA6* was shared across all three regional A→N subtype pairs. To assess how the expression of L-R pairs underlying neuron-astrocyte communication changes over the course of development, we examined the overlap of cortical glutamatergic L2/3IT neuron to cortical astrocyte L-R pairs at different developmental time points. In contrast to the expression of rDEGs, a larger proportion (20/90, 22%) of L-R pairs were shared between all time points (Fig. 4D), suggesting that these putative mediators of neuron-astrocyte communication emerge early and are maintained throughout development. However, 15/90 (17%) L-R pairs emerged at 7 months and were maintained throughout adulthood. At later developmental time points, many more L-R pairs were shared between ages than were unique (Fig. 4D). This, along with the increased proportion of rDEGs shared across later time points (Fig. 2D), suggests that the expression of molecules underlying neuron-astrocyte communication stabilizes postnatally in marmosets at some point between 0 and 7 months.

A single astrocyte may communicate with dozens of neurons and thousands of synapses^54,56^. This implies that different neuron types within a single brain region may have developed specialized means of communicating with the same astrocyte. We examined unique L-R pairs (meaning that the pair did not rise to specificity significance for other striatal neurons, see Methods) between striatal neuron subtypes and striatal astrocytes, focusing on a single time point (adulthood) when L-R expression has stabilized (Fig. 4D). Striatal cholinergic interneurons (ChINs or *CHAT*+ interneurons) had the most unique predicted L-R pairs with astrocytes, and vice-versa, while *DRD1*+ medium spiny neurons and astrocytes had no unique predicted L-R pairs (Fig. 4E(i), Table S15). Examination of the expression of predicted ChIN-striatal astrocyte ligands and receptors shows that some are more specific to these two cell types than others (Fig. 4E(ii)). For example, *TNC*→*ITGA7* expression was very specific to ChINs and striatal astrocytes while *NLGN3*→*NRXN1* was shared by other striatal neuronal clusters.

Overall, patterns of predicted neuron-astrocyte and astrocyte-neuron communication in mouse were similar to marmoset (Fig. S12), including implication of neurexin and neuroligin, contactin, fibroblast growth factor and receptor, and neural cell adhesion molecular families. Many of the region-specific L-R pairs from one region were from the same families as the unique pairs from other regions (Table S14). Mouse thalamus had more unique L-R pairs between its neurons and astrocytes when compared to the cortex and striatum at P4 and P90 (Fig. S12C). We then compared L-R pairs specifically from cortical L2/3IT glutamatergic neurons to cortical astrocytes across ages. Unlike in marmoset, mice had more age-specific L-R pairs at earlier time points (particularly at P4, 17/84 or 20% of all L-R pairs unique at this time point) before stabilizing with more shared L-R pairs at later time points, suggesting that mediators of neuron-astrocyte interactions emerge relatively later in mouse (Fig. S12D). Only 11% of L2/3IT→astrocyte L-R pairs were shared across the lifespan. We also compared L-R pairs between subclusters of mouse striatal neurons to striatal astrocytes. Many of the unique L-R pairs were also attributed to ChINs, but the mouse astrocytes also had additional unique L-R pairs from immature *Top2a*+ neurons, *Pvalb*+ MGE neurons, and *Drd3*+ LGE neurons (Fig. S12E(i), Table S14). Taken together, these results suggest that many L-R pairs potentially underlying neuron-astrocyte communication are shared across developmental time points and regions in both species. However, more neuron-astrocyte predicted L-R pairs emerged later in development, and neuronal subtypes and regional astrocyte populations also had near-unique predicted L-R pairs.

### Maturation of telencephalic glia lags behind diencephalic glia

In mouse, initiation of gliogenesis in the diencephalon precedes that in the telencephalon by approximately 1 gestational day (E13.5 vs E14.5^57,58^). To determine whether relative immaturity of diencephalic glia compared to telencephalic glia could explain the robust regional expression differences we observed at each sampled time point (Figs. 2, 3), we examined the developmental trajectory of astrocytes in pseudotime, a prediction of position along a low-dimensional developmental trajectory based on RNA expression only, using Palantir^59^. We first calculated pseudotime trajectories for the oligodendrocyte lineage, which follows a known differentiation trajectory from oligodendrocyte progenitor cells (OPCs) to committed oligodendrocyte precursors (COPs) to newly formed oligodendrocytes (NFOLs) to myelin-forming and mature oligodendrocytes (MFOLs, MOLs)^60^. Pseudotime analysis with Palantir recovered this developmental trajectory in both species (Fig. S13A-B). Furthermore, it underscored the precocious myelination in the marmoset brain compared to mouse, as evidenced by larger pseudotime values, indicating higher maturity along the pseudotemporal trajectory (values range from 0 to 1), from NFOLs/MOLs in GD135, neonate, and 7-month marmoset oligodendrocytes compared to mouse E18.5, P4, and P14 time points (Fig. S13C-D).

**Figure 5.**
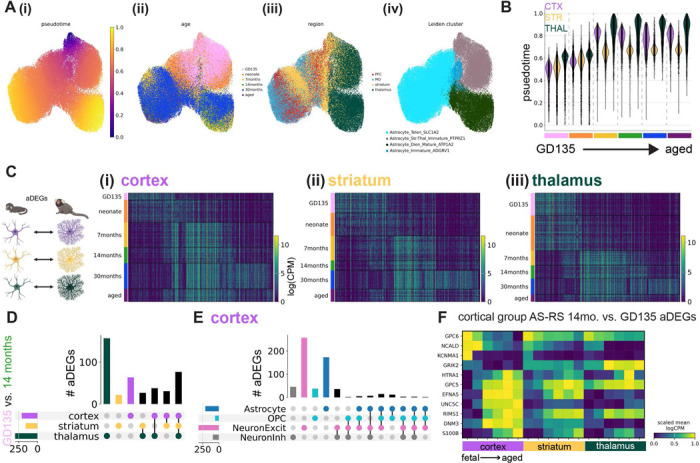
The postnatal developmental specification of marmoset astrocytes within and across brain regions. **A,** Integrated UMAP embeddings of 103,009 marmoset astrocytes colored by **(i)** Palantir-predicted pseudotime **(ii)** developmental time point **(iii)** brain region (assigned) and **(iv)** Leiden cluster assignment. **B,** Violin plot (scanpy’s default) showing the estimated distribution of pseudotime values for the astrocytes in **(A)** grouped by region within each developmental time point (color code as in **A(i)**). Vertical dashed lines indicate separation between time points. **C,** Heatmaps (rows corresponding to nuclei and columns to gene) showing expression in logCPM of astrocyte age differentially expressed genes (aDEGs) in astrocytes from **(i)** cortex, **(ii)** striatum, and **(iii)** thalamus, grouped by developmental time point as indicated on the left of the heatmap. The strategy for calculating aDEGs is schematized on the left. **D**, UpSet plot showing the number of overlapping GD135 vs. 14-month astrocyte aDEGs between cortex, striatum, and thalamus. The colored dots below each vertical bar indicate which region(s) share that set of aDEGs, while the colored horizontal bars indicate the total number of cortex-thalamus aDEGs for each region. **E**, UpSet blot (as in **(D)**) showing the number of overlapping GD135 vs. 14-month cortical astrocyte aDEGs between OPCs (light blue), astrocytes (dark blue), excitatory neurons (pink), and inhibitory neurons (gray). **F,** Matrix plot showing mean expression of selected cortex group AS-RS aDEGs (rows) in marmoset astrocytes grouped by region and developmental time point (columns, blocked by region first and then by increasing age within each region block). Expression units of mean logCPM are standardized between 0 and 1 by subtracting the minimum and dividing by the maximum for each trait.

In astrocytes from both species, pseudotime analysis revealed a transcriptional developmental trajectory within astrocytes that aligned with the known developmental trajectory and annotation of mature and immature Leiden clusters (Fig. 5A, Fig. S13E-F). In marmoset, GD135 astrocytes had on average the lowest pseudotime values, followed by neonate astrocytes, then by 7-month and older astrocytes, which all showed similar pseudotime value distributions (Fig. 5B). Pseudotime values were slightly higher in mature diencephalic versus mature telencephalic astrocytes in both species at mature time points (Fig. 5A(iii), Fig. S13E-G). Next, for each region we binned astrocytes by pseudotime quintile rather than by actual age and recomputed rDEGs. As with rDEGs grouped by actual age, the number of rDEGs increased from pseudotime bin 1 (PT1) to pseudotime bin 5 (PT5): 56 rDEGs at PT1, 114 at PT2, 131 at PT3, 168 at PT4, and 185 at PT5. We found that matching by predicted maturational stage largely recapitulated the original rDEGs calculated from actual age: 52% of PT1 cortex-thalamus rDEGs overlapped with GD135 rDEGs, 77% of PT2 rDEGs overlapped with neonate rDEGs, 39% of PT3 rDEGs overlapped with 7-month rDEGs, 80% of PT4 rDEGs overlapped with 14-month rDEGs, 82% of PT5 rDEGs overlapped with adult rDEGs, and 86% of PT5 rDEGs overlapped with aged rDEGs. This suggests that regional imprinting of astrocytes is not simply driven by relative differences in the birth timing of cells across the different brain structures.

### Age-dependent refinement of astrocyte identity

Our previous analysis focused on molecular distinctions that emerge in astrocytes that reside in different brain regions (Figs. 2,3). We next sought to determine the sequence of molecular changes that unfold in astrocytes within a given region over time. We calculated age differentially expressed genes (aDEGs) within each brain region from metacells of each age (see Methods). In each brain region, there were over 100 unique aDEGs (unique after pooling pairwise age combinations). In marmoset, the largest fraction of aDEGs distinguished GD135/neonate from 7-month and older astrocytes (Fig. 5C). aDEGs enriched in the 29–32 month dataset could conceivably arise from the different sample preparation and reference genome used in our previous study^7^; for this reason we used the 14-month time point to further assess age-related changes across regions.

Examining the overlap of marmoset astrocyte GD135 vs. 14-month aDEGs between brain regions (409 aDEGs in total), we found that ~19% were shared between cortex, striatum, and thalamus. The striatum had a modest number of GD135 vs. 14-month aDEGs not shared with other regions (21/409), while the cortex had 3-fold more (63/409), and the thalamus had the most (156/409), as expected given the stark regional heterogeneity between telencephalon and diencephalon (Fig. 2). Additionally, we found that few to no GD135 vs. 14-month aDEGs were shared between astrocytes, OPCs, and excitatory neurons or astrocytes, OPCs, and GABAergic neurons in the cortex (Fig. 5E). We found similar results in mice, where we calculated P4 vs. P90 rDEGs, as mouse E18.5 astrocytes were transcriptionally immature relative to marmoset GD135 astrocytes and GD135-P4 timepoints appear to have better correspondence, as discussed in the next section). One notable difference from marmoset was that early adolescent (P14) astrocytes in mice expressed many aDEGs shared with embryonic and neonate timepoints (Fig. S14A-C).

To aid the interpretation of region-shared vs. region-specific aDEG profiles, we divided astrocyte aDEGs into 3 groups: group CA-RS (cell type agnostic, region specific) are astrocyte aDEGs that are also aDEG in neurons and OPCsfor a given brain region; group AS-RA (astrocyte specific, region agnostic) are shared between astrocytes of all brain regions; and group AS-RS (astrocyte specific, region specific) are specific to astrocytes in a given brain region. CA-RS aDEGs are shared between all analyzed cell types in a given region and not shared with other regions. We found very few (3 or less) CA-RS aDEGs within marmoset GD135–14-month comparisons. Full lists of all mouse and marmoset aDEGs for P4 vs. P90 and GD135 vs. 14-month comparisons respectively, including CA-RS, AS-RA, and AS-RS lists, are provided in Tables S16-17.

Group AS-RA aDEGs reflect the universal aspects of astrocyte transcriptional identity at each developmental stage, regardless of brain region. We found 74 of these within marmoset GD135 vs. 14-month aDEGs and 56 within mouse P4 vs. P90 aDEGs. In both species, the predicted functions of group AS-RA aDEG protein products were varied and included cell adhesion/repulsion, gap junction formation, neurotransmitter signaling, cholesterol efflux, potassium transport (mouse), and BDNF signaling (marmoset), among others. Group AS-RS aDEGs reflect the brain region’s influence on the maturation of astrocytes *only* in a given brain region. In contrast to group CA-RS (above) they are unique to astrocytes (vs. OPCs and glutamatergic and/or GABAergic neurons) in a given brain region, and (unlike group AS-RA) are not shared with astrocytes in other brain regions. We found 20 of these in striatum, 51 in cortex, and 125 in thalamus within GD135 vs. 14-month aDEGs. In both species, the developmental pattern of selected cortical group AS-RS aDEGs was similar but not identical in the striatum, and more dissimilar with the thalamus (Fig. 5G(ii), Fig. S14D).

### Conservation and divergence of astrocyte patterning in mouse and marmoset

Hundreds of differentially expressed genes distinguish adult human and mouse astrocytes^61^, and engrafting human glial progenitors into mouse brain results in mature astrocytes that retain certain functional and morphological human-specific astrocyte characteristics^62^. This suggests that aspects of an astrocyte’s developmental program are cell intrinsic and are shaped by species-specific features of its genome. We therefore aimed to compare transcriptional signatures of telencephalic and diencephalic regional astrocyte populations between marmoset and mouse. We integrated a randomly downsampled subset (100,00 nuclei each) of mouse and marmoset nuclei (all cell types included) using 547 highly variable 1:1 ortholog genes selected from top differentially expressed genes of superclusters (related groups of Leiden clusters) shared across species with the semi-supervised variational auto-encoder scANVI^63^ (see Methods). The resulting integrated UMAP plot showed broad conservation of superclusters between mouse and marmoset, despite differences in cell type proportions across development (Fig. S15A-B).

**Figure 6.**
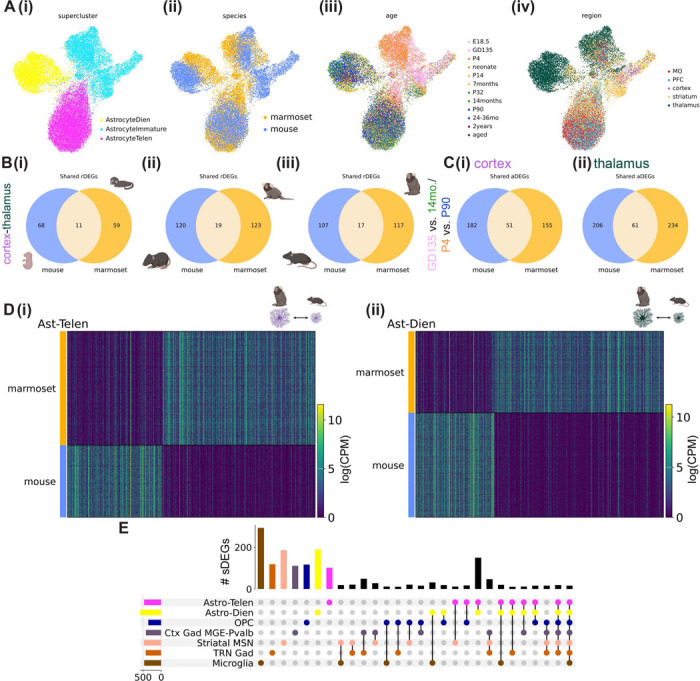
Conservation and divergence of the development of astrocyte heterogeneity in mouse and marmoset. **A,** scANVI-integrated UMAP embeddings of marmoset and mouse astrocytes, colored by **(i)** supercluster, **(ii)** species, **(iii)** age, and **(iv)** region. **B,** Venn diagrams showing regional differentially expressed genes (rDEGs) between cortex and thalamus astrocytes shared across mouse and marmoset at **(i)** fetal, **(ii)** early adolescent, and **(iii)** young adult time points. **C,** Venn diagram showing age differentially expressed genes (aDEGs) shared between mouse and marmoset astrocytes within the **(i)** cortex and **(ii)** thalamus. **D,** Heatmaps showing expression in logCPM of species differentially expressed genes (sDEGs) between marmoset and mouse within **(i)** telencephalic astrocytes and **(ii)** diencephalic astrocytes. **E,** UpSet plots showing shared sDEGs across superclusters, comparing telencephalic astrocytes, diencephalic astrocytes, oligodendrocyte precursor cells (OPCs)medial ganglionic eminence-derived and PVALB+ cortical GABAergic neurons (Ctx Gad MGE-PVALB), thalamic reticular nucleus (TRN) GABAergic neurons, striatal medium spiny neurons (MSNs), and microglia. The colored dots below each vertical bar indicate which supercluster(s) share that set of sDEGs, while the colored horizontal bars indicate the total number of mouse-marmoset sDEGs for each supercluster. For simplicity, only supercluster combinations with 10 or more shared sDEGs are shown.

Species-integrated astrocytes partitioned into three superclusters that segregated by developmental stage and by brain structure (diencephalon vs telencephalon) (Fig. 6A). This indicates that at the level of broad cephalic domains, regionalization patterning is conserved between the two species. Mature telencephalic astrocytes showed better species integration than diencephalic or immature astrocytes, and immature mouse astrocytes composed a distinct cluster (Fig. 6A). The mouse-specific cluster includes the *Top2a*+ immature astrocyte population seen in the neurogenic subventricular zone throughout the lifespan (Fig. S16) which forms part of the rostral migratory stream. This finding implies that relative to birth, mouse astrocytes are less mature than their marmoset counterparts, in line with our findings about oligodendrocyte maturation (Fig. S13A-B). Another method called SATURN^64^, which avoids 1:1 mapping of genes, had largely concordant results (Fig. S15D-E).

We next tested whether genes that best distinguished astrocytes from a given brain region in one species were more likely than chance to be rDEGs in the other species. Focusing on astrocyte cortex-thalamus rDEGs at each developmental time point, we found that the majority of rDEGs were not shared across species, and that the proportion of shared rDEGs decreased only slightly from fetal to early adolescence time points, from ~14–16% to ~13–14% in both species (Fig. 6D, full list of species-overlapping and species-unique cortex-thalamus astrocyte rDEGs at each developmental time point in Table S18). A chi-square test revealed that the proportion of mouse rDEGs that were also marmoset rDEGs was not greater than chance (see Methods, p-value from a Chi-square test = 0.051 at fetal time point and 0.18 at adult time point), and that the proportion of marmoset rDEGs that were also mouse rDEGs was greater than chance only at early time points (p-value from a Chi-square test = 0.038 at fetal time point, 0.043 at neonate time point, and 0.20 at adult time point).

The majority of astrocyte aDEGs in cortex and thalamus were not shared between species. 22% of mouse cortical P4-P90 aDEGs were shared with marmoset GD135–14-month aDEGs, and a similar proportion (23%) of thalamic P4-P90 aDEGs were shared with marmoset GD135–14-month aDEGs. Marmoset shared 25% of its cortical aDEGs and 21% of its thalamic aDEGs with mouse. In the cortex, group AS-RA aDEGs were more likely to be shared across species (16% and 21% shared for marmoset and mouse, respectively) than group AS-RS aDEGs (<2% shared for both species), or aDEGs assigned to neither group (15% and 12% shared for marmoset and mouse, respectively). A full list of species-overlapping and species-unique astrocyte aDEGs is provided in Table S19.

Next, we directly tested for differential expression of 1:1 orthologs between species within shared superclusters. We calculated species differentially expressed genes (sDEGs) based on 1:1 orthologs between species within each integrated supercluster using our metacell method (see Methods). Complete lists of sDEGs for each supercluster analyzed are provided in Table S20. We found hundreds of sDEGs in both telencephalic (464 total) and diencephalic (579 total) astrocytes whose expression could clearly distinguish between marmoset- and mouse-derived populations (Fig. 6D, Table S21). 50% of the genes that distinguish diencephalic astrocytes between species were shared with telencephalic astrocytes. These telencephalic-diencephalic astrocyte shared sDEGs made up a larger fraction (62%) of telencephalic astrocyte sDEGs. This suggests that evolution has acted on the astrocyte class as a whole, while also shaping divergent regional astrocyte programs between species. Additionally, we found that most telencephalic and diencephalic astrocyte sDEGs were not shared with other superclusters, including (listed in order of the number of unique sDEGs for that supercluster) OPCs, cortical MGE-derived *PVALB*+ interneurons, GABAergic TRN neurons, striatal MSNs, and microglia (Fig. 6E). This result underscores that evolutionary divergence of a cell type’s transcriptome unfolds at different rates across cell types^65–67^. Taken together, these findings support both conservation and divergence of postnatal astrocyte regional specialization in mouse and marmoset.

### Astrocytes have regionally divergent protein expression but converge on similar morphologies

Many of the genes we found to vary in astrocytes by region, age, and species implicate processes involved in morphological specification. Indeed, astrocyte morphology, which is highly ramified and complex, is essential for their specialized functions: end feet contact blood vessels to help form the blood-brain barrier and shuttle water and nutrients, while terminal processes closely appose synapses to uptake ions and neurotransmitters^32^. Because many of these morphological features exist at the sub-micron scale, conventional light microscopy is not sufficient to visualize the full morphological complexity of astrocytes^33^. We wondered whether nanoscale astrocyte morphology might also be regionally specialized between gray matter regions in cortex, striatum, and thalamus, as recently demonstrated using diffraction-limited approaches for 13 regions in the mouse brain^68^. Thus, we used expansion revealing (ExR), a new variant of protein decrowding expansion microscopy^34^, to visualize nanoscale astrocyte morphology and compare morphological properties in the PFC, striatum, and thalamus.

**Figure 7.**
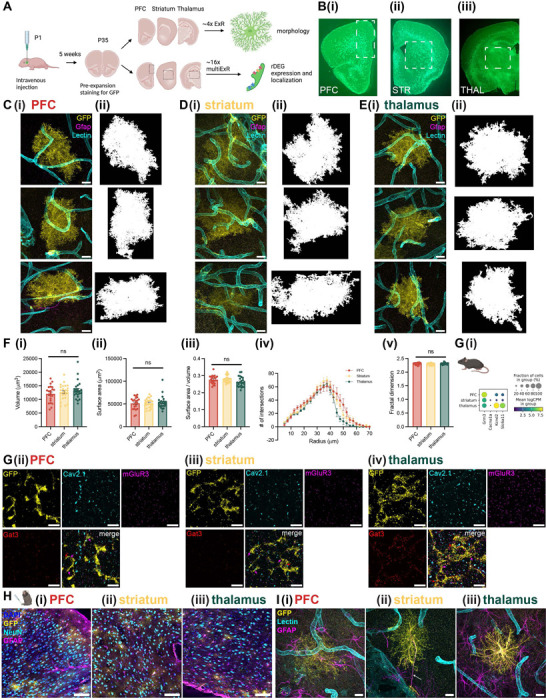
Expansion microscopy of virally-labeled astrocytes in the mouse and marmoset brain. **A,** Viral labeling approach for mouse astrocytes (see Methods for details). Created using BioRender.com. **B,** Brain slice hemispheres containing regions of interest after pre-expansion staining for GFP, as visible through the eyepiece on a dissecting microscope under blue light illumination. Boxed regions indicate approximate dissected and imaged regions for **(i)** PFC, **(ii)** striatum and **(iii)** thalamus. **C-E, (i)** Maximum intensity projections of background-subtracted images of ~4x expanded astrocytes in the **(C)** prefrontal cortex, **(D)** striatum, and **(E)** thalamus, co-stained with GFP and the blood vessel marker Lectin. Shown are 3 examples of high GFP-expressing astrocytes from 3 separate mice. Scale bar, 10μm in biological units. Contrast was adjusted to 35% saturation (Fiji’s “auto”). See Supplementary Movies 1–18 for 3D-projected image volumes **(ii)** Maximum intensity projections of 3D binary segmentations of the astrocytes in **(i)** used as input for morphology analysis. **F,** Bar plots showing quantified morphological properties for mouse astrocytes from PFC, striatum, and thalamus (n = 22–24 astrocytes from 2 female and 6 male mice for each region, with statistical significance determined using a linear mixed effects model with “animal” as the random effect group variable): **(i)** volume, **(ii)** surface area, **(iii)** surface area / volume, **(iv)** Sholl analysis (number of intersections with concentric shells as a function of radius), and **(v)** fractal dimension by the box counting method (see Methods). **G, (i)** Dot plot showing mean expression in astrocytes and percent of astrocytes expressing, by region, of rDEG protein products and Cav2.1 (*Cacna1c*) stained using multiExR, in the P32 mouse brain. **(ii-iv)** Maximum intensity projection composites of registered, cropped (in x and y and to 32 z-slices) regions of GFP-labeled astrocyte processes co-stained with Cav2.1, mGluR3, and Gat3 over 2 rounds of staining using the multiExR protocol. Colored arrows indicate colocalization of the protein in the corresponding pseudo-colored channel on astrocyte processes. Shown are processes from astrocytes in the **(ii)** PFC **(iii)** striatum and **(iv)** thalamus of the same mouse. Scale bar, 1μm. See Fig. S17C for full fields of view. Contrast was manually adjusted by setting minimum and maximum intensity values. **H,** Extended focus-projected (in TissueGnostics software) composite field of view of virally-labeled astrocytes in an adult female (19–241) marmoset **(i)** PFC, **(ii)** striatum, and **(iii)** thalamus, co-stained for GFP, GFAP, and NeuN. Scale bar, 100μm. Images were manually contrast adjusted to eliminate autofluorescence background and are not equally contrasted across regions. **I,** Maximum-intensity projections of background-subtracted composite images of virally-labeled, ~3x expanded astrocytes from the marmoset in **(G)**, co-stained for Lectin and GFAP. Shown are astrocytes from the **(i)** PFC, **(ii)** striatum and **(iii)** thalamus as dissected in Fig. S17D. Scale bar, 10μm in biological units. White arrow in **(ii)** indicates an astrocyte process extending ~50μm to contact a large-diameter blood vessel. Images were contrast adjusted for Lectin and GFAP channels at 35% saturation (Fiji’s “Auto”), and manually for GFP to increase brightness and reduce background signal.

We used a viral approach to label astrocytes for expansion. Specifically, we injected neonate Aldh1l1-Cre mouse pups with AAV PHP.eB CAG-FLEX-eGFP-WPRE^69^ (Addgene #51502) via the facial vein (Fig. 7A). After 5–6 weeks of viral expression and pre-expansion staining for GFP, astrocytes were brightly labeled and distributed throughout the brain (Fig. 7B). We found that a single expansion step, yielding an expansion factor of ~4x, was sufficient to visualize complex astrocyte morphology in gray matter regions of the mouse PFC, striatum, and thalamus (Fig. 7C-E(i)).

Qualitatively, labeled astrocytes were similar in size, shape, and level of morphological complexity across brain regions (Fig. 7C-E). To quantitatively assess morphological differences across regions, we created 3D binary segmentations of manually cropped astrocyte volumes using MATLAB (Fig. 7C-E(ii)). We calculated the volume and surface area (measures of size), surface area to volume ratio (S:V, a measure of shape, inversely proportional to size), fractal dimension (FD) using the box counting method^70^ (a measure of complexity and self-similarity), and branching complexity via Sholl analysis^71^, all measures which have been used to characterize astrocyte morphology^33,72^. After correcting for multiple comparisons across the 4 measures (volume, surface area, S:V, and FD), we found no statistically significant differences between astrocytes from different brain regions (Fig. 7F, n = 22–24 astrocytes from 2 female and 6 male mice for each region, linear mixed effect model with “animal” as the random effect group variable, see Table S22 for results and a summary of z-scores and corrected and uncorrected p-values on each coefficient). Similarly, Sholl analysis revealed a similar shape to the distribution of the number of intersections between the segmented astrocyte and 3D concentric shells as a function of shell radius between PFC, striatum, and thalamus (Fig. 7F(iv)). However, there were subtle differences revealed by Sholl analysis, including thalamic astrocyte intersections decreasing more rapidly than cortical or striatal at larger radii of 50–70μm. This result suggests distal thalamic astrocyte processes may be less morphologically complex than their cortical or striatal counterparts.

We next probed rDEG protein product expression level and localization in astrocytes at the nanoscale using multiplexed expansion revealing (multiExR), a version of ExR that enables super-resolution imaging of up to 20 protein targets in a single field of view (Kang et al., 2024, in press). We processed tissue from 2 of the virally-labeled Aldh1l1-Cre mice used for ~4x ExR, achieved an expansion factor of ~16x, and proceeded with 2 rounds of staining (see Methods, Table S23). We observed localization of the synaptic protein Cav2.1 (*Cacna1c*) and rDEG protein products mGluR3 (*Grm3*, telencephalic rDEG) and Gat3 (*Slc6a11*, thalamic rDEG) on and near astrocyte processes (Fig. 7G(ii-iv), colored arrows). We examined contrast-adjusted maximum intensity projections from 3–6 fields of view per region from 2 mice, and found that Gat3 expression was higher in thalamic astrocytes, as expected (Fig. 7G(i)). The expression level of mGluR3, predicted to be a telencephalic rDEG, was similar across regions, perhaps reflecting the smaller differences in mean expression level and fraction of cells positive between regions for this rDEG (Fig. 7G(i)). We also observed a few loop-like structures on astrocyte processes at this resolution, consistent with the “reflexive loops” visible with other super-resolution techniques^73^ (white arrows in Fig. S17C). Taken together, these results support the differential expression of Gat3 protein between telencephalic and diencephalic astrocytes, and reveal the localization of rDEG protein products on and near astrocyte processes, in close proximity to neuronal synapses.

With the goal of comparing astrocyte morphology across species, we attempted a similar viral labeling approach in marmoset. Because there are no transgenic marmosets expressing Cre recombinase in astrocytes, we used the astrocyte-specific *gfaABC1D* (cloned from pZac2.1 gfaABC1D-tdTomato^74^, Addgene #44332) promoter to deliver GFP to marmoset astrocytes, which had been previously demonstrated to work in macaques^75^. We packaged this construct in BI103, a new AAV capsid that can cross the blood-brain-barrier in marmosets^7^, injected via the tail vein at 1×10^14^ vg/kg, waited 68 days before tissue harvest, and perfused with the ExR fixative (30% acrylamide and 4% paraformaldehyde). We observed expression of GFP in astrocytes in our target brain regions (Fig. 7H), as well as in a second marmoset injected at a lower dose (Fig. S17E). We proceeded with ~3x expansion and were able to visualize complex astrocyte morphology alongside GFAP and Lectin staining for astrocyte processes and blood vessels, respectively (Fig. 7I).

The relatively dimmer signal in marmoset astrocytes (Fig. 7I, Fig. S17D(ii-iv)), and our inability to capture the full volume of marmoset astrocytes within the boundaries of the gel, precluded quantitative cross-region or cross-species comparison of astrocyte morphological properties. Qualitatively, compared to mouse, marmoset astrocytes were slightly larger, but had a similar level of morphological complexity and also made several contacts with nearby blood vessels. Of note, GFAP staining was qualitatively more common in marmoset astrocytes (Fig. 7I). We also found at least one instance of an astrocyte (in this case, in the striatum) extending a process a distance of ~50μm to contact a large blood vessel (Fig. 7I(ii), white arrow), reminiscent of the varicose projection astrocytes found in the great ape cortex^76^. We observed similar extended processes in the mouse (Fig. S17A). These results illustrate the utility of ExR for characterizing primate astrocyte morphology across brain regions. With further optimization of the viral labeling approach in marmoset and experiments in additional animals, a quantitative description of marmoset morphology across regions is possible.

## Discussion

Astrocytes are a ubiquitous, versatile brain cell type with increasingly appreciated roles in health and disease. While their regional molecular heterogeneity has been evident for some time^77,78^, the source of this regional heterogeneity, in particular, the relative contributions of embryonic patterning versus response to environmental cues after birth, is not well understood^79^. To help bridge this knowledge gap, we generated a unified, multi-region, postnatal developmental snRNAseq of mouse and marmoset brain cells. Because our dataset contains all brain cell types, we anticipate this atlas will be a valuable resource for the field. As such, we have made both raw and processed data publicly available on NeMO and the Broad Single Cell Portal, respectively (see Data and Code Availability). The latter is useful for exploring cell type clusters and querying the expression pattern of genes of interest across ages and regions, and does not require coding expertise.

We found that astrocytes were regionally patterned before birth in both species, a discovery that was not unexpected given the prevalence of homeobox patterning genes among astrocyte regionally differentially expressed genes^8^, evidence from a recent study showing regionally patterned glioblasts in the first-trimester human brain^6^, and older lineage tracing showing regional allocation of astrocytes based on the region of their originating radial glia^24^. However, we found dramatic changes in astrocyte regional identity between birth and early adolescence, in line with their maturation during this period. This period also coincides with peak synaptogenesis, pruning, and myelination^80^, consistent with the notion that astrocyte specialization depends on the activity of neighboring cells^81^.

The functions of embryonically-patterned and postnatally-acquired astrocyte rDEGs were varied, but implicate known astrocyte processes, including supporting synaptic transmission, ion transport, neurotransmitter uptake, cell-cell adhesion, and morphological specification. The function of some rDEGs, including *SLC6A11* (GAT-3) and *SPARC*, has been studied in astrocytes, and shown to be important in modulating the effects of brain injury^82^ and controlling synaptogenesis^48^, respectively.

We found that neuron-astrocyte and astrocyte-neuron predicted ligand-receptor pairs, many of which were specialized for distinct neuronal subtypes, were upregulated during postnatal development into adulthood, again supporting the hypothesis that astrocytes specialize in postnatal development to meet the needs of local neurons. Despite the striking regional heterogeneity of astrocytes, we found that many predicted neuron-astrocyte and astrocyte-neuron ligand-receptor pairs were shared across regions. Even those not shared across regions were functionally similar, suggesting neurons and astrocytes have developed a common language of molecular communication across the forebrain. Indeed, some of our rDEGs were members of the same protein family or functional class, pointing to variations on a common theme of neuron-astrocyte crosstalk across brain regions. Many of the top predicted neuron-astrocyte ligand-receptor pairs, such as neurexins and neuroligins, are more traditionally associated with neuron-neuron contact at the synapse^83^. However, adhesion molecules such as ephrins, neurexins/neuroligins, and NrCAMs have been shown to play important roles in neuron-astrocyte communication^44^.

In both species, we found hundreds of age differentially expressed genes (aDEGs), many of which astrocyte-specific but region-agnostic, some of which were astrocyte-specific and region-specific, and very few of which were cell type-agnostic but region-specific. The thalamus had the most unique astrocyte developmental gene expression signature of the three brain regions, suggesting that thalamic astrocytes undergo distinct developmental changes from their telencephalic counterparts. Our astrocyte-specific, region-agnostic aDEGs can be interpreted as a core forebrain astrocyte developmental program, and were more likely to be shared across the species. For example, in marmoset, this included *NTRK2*, which encodes the BDNF receptor TrkB, the short isoform of which has been shown to be essential for astrocyte morphogenesis^84^. Perhaps unsurprisingly, several of our region-specific aDEGs were also rDEGs, and/or had a high degree of functional overlap with rDEGs.

In both species, most astrocyte rDEGs, aDEGs, and sDEGs were not shared with OPCs or neurons, suggesting that astrocyte region and age specializations are unique, as opposed to general to all radial-glia derived cell types in the same region, developmental time point, or species. This suggests either that regional gene expression signatures change throughout neuro- and glio-genesis, or that the downstream transcriptional effects of this early regional patterning depend on the daughter cell’s fate. Evidence for both exists in the cortex^85^. Why neurons and astrocytes “remember” their region of origin, albeit in different ways, while the oligodendrocyte lineage does not retain most of its regional signature, at least at the transcriptomic level, is an outstanding question for future study.

The present study characterized two mammalian neuroscience model species, mouse and marmoset. While mice and humans have a high degree of genetic conservation^86^, mice have significant limitations as a model for studying the human brain including lack of well-developed prefrontal cortex, complex social behaviors, poor visual acuity. In light of these limitations, non-human primates, with whom we share much closer genetic ancestors, have long served as more translationally-relevant models of human behavior and neuropathology. The common marmoset has become an increasingly popular non-human primate model in neuroscience studies due to its faster generation time for genetic engineering, shorter lifespan than other larger primates for developmental and late-onset disease studies, and complex social behaviors^87^.

Our data suggest that the development of astrocyte regional heterogeneity, marked by embryonic regional patterning along cephalic boundaries followed by dramatic postnatal specialization, is broadly conserved between mouse and marmoset. However, the expression of many rDEGs and aDEGs differs across species, and we identified hundreds of species differentially expressed genes within both telencephalic and diencephalic astrocytes (Fig. 5). Taken together, these findings suggest that each species may have evolved by recruiting different set of genes that facilitate postnatal regional specialization of astrocytes. We found that many cell types in the marmoset brain are transcriptionally more mature at time of birth than their mouse counterparts, in line with previously documented precocious development in early postnatal marmosets^88^. This species divergence in transcriptional maturity at time of birth suggests that researchers should use caution when comparing early postnatal time points between rodents and NHPs, especially in light of differences in developmental tempo between species^89^.

We used expansion revealing (ExR) combined with a viral astrocyte labeling approach to circumvent the diffraction limit of light microscopy and the limitations of immunostaining, respectively, to visualize nanoscale astrocyte processes in both species. We accomplished brain-wide viral labeling of astrocytes in the marmoset via intravenous injection and used expansion microscopy to visualize primate astrocyte morphology, which we anticipate will be of use to the primate research field. Our quantitative profiling of astrocyte morphology in the mouse brain shows that morphological differences between astrocytes from gray matter regions in PFC, striatum, and thalamus are subtle. Though prior studies have characterized more appreciable morphological differences in mouse astrocytes across brain regions, these are not directly comparable to ours, as they examined different brain regions, used conventional light microscopy, and/or a different labeling strategy such as immunohistochemistry^54,68,90^. It is possible, and indeed likely given that several astrocyte rDEGs are related to morphological specialization, that there are additional differences in aspects of astrocyte morphology not quantified in the present study. We demonstrate that expansion microscopy, particularly ExR, offers an inexpensive and accessible alternative to other super-resolution approaches for characterizing nanoscale astrocyte morphology. We anticipate other groups might adapt this approach to studying morphological changes in astrocytes after manipulation and/or in disease contexts.

There are several notable limitations to the current study, only some of which we discuss here. The first is the reliance on 10x Chromium snRNAseq, which is subject to dropout and 3’ bias, and produces short reads that cannot be used to map splice variants or many single-nucleotide polymorphisms that may differ between cell types and species. The second is the relatively small sample size, especially for marmosets due to practical limitations including cost, which limits our ability to compare between sexes. Third, we relied on pathway analysis to summarize patterns and deduce functional implications arising from sets of rDEGs. Our use of WebGestalt did not incorporate any fold-change or p-value information for genes, treating each DEG equally regardless of its differential expression level, which may skew results. Furthermore, pathway analysis is only as accurate as the underlying annotations, which can be lacking for glial biology. Finally, many genes are involved in several pathways. For these reasons, we encourage interested readers to directly examine our DEG lists provided in the Supplementary Tables.

Fourth, our approach for labeling astrocytes for morphological analysis relies on viral infection and manual identification of the brightest astrocytes for imaging, which may be biased towards a certain astrocyte subtype, such as those with more vascular contacts. Future studies might explore whether local administration of virus, alternative capsids, alternative transgenic lines, and or alternative promoters, would label a broader range of astrocytes. Finally, we relied on two spatial multiplexing techniques, RNAscope HiPlex and multiExR, to assess rDEG mRNA and protein expression *in situ*, respectively. Any multiplexing technique that involves repeated stripping and restaining suffers from some level of reduced fluorescence intensity in later rounds, as well as some amount of registration error. Therefore, any researcher interested in following up on a gene or protein of interest that was imaged in a later round should perform additional confirmatory studies with a single round of imaging.

Taken together, our data support a model of astrocyte regional specialization that includes both embryonic patterning and postnatal specialization in response to local environmental cues, including synapse formation and neuronal activity, as has been previously suggested^78^. To determine whether or not early transcriptional patterning is required for proper postnatal astrocyte specialization for such a role, a cross-region astrocyte heterotopic transplant would be illuminating. That is, would a thalamic-born astrocyte be able to acquire the transcriptional and morphological profile of a cortical astrocyte if transplanted in early postnatal life? Evidence from such an experiment in septal astrocyte populations suggests the answer is yes^28^. Alternatively, but not mutually exclusively, early developmental regional patterning may “prime” astrocytes to receive and react appropriately to the signals they receive in their local niches later in development, as a recent study has shown in the context of GABA-induced morphogenesis^91^. We anticipate the current study will be a useful starting point for hypotheses such as these.

### Data and Code Availability

Raw data (sequencing reads in fastq format) for all 10x Chromium snRNAseq samples and CellBender-cleaned aligned counts matrices (in .h5 format) are publicly available for download on the Neuroscience Multi-omic Data Archive (NeMO) at: https://data.nemoarchive.org/biccn/grant/u01_feng/feng/transcriptome/sncell/10x_v3.1/. Pre-processed, clustered, and annotated data (in .h5ad format) is available for download, exploration, and gene search on the Broad Single Cell Portal at: https://singlecell.broadinstitute.org/single_cell/study/SCP2719/a-multi-region-transcriptomic-atlas-of-developmental-cell-type-diversity-in-mouse-brain (mouse) and https://singlecell.broadinstitute.org/single_cell/study/SCP2706/a-multi-region-transcriptomic-atlas-of-developmental-cell-type-diversity-in-marmoset-brain#study-summary (marmoset). Registered RNAscope HiPlex FISH image stacks, raw and registered expansion microscopy image volumes and binarized 3D tracings (both in .tif format) are available for download on BossDB at https://bossdb.org/project/schroeder2024. Custom scripts used to analyze data are available at https://github.com/Feng-Lab-MIT/AstrocyteHeterogeneity.

## Methods

### Marmoset tissue harvest for snRNAseq.

Common marmosets were housed in AAALAC-accredited facilities at MIT, in spacious holding rooms with a 12 hour light/dark cycle, temperature 74.0 ± 2.0°F (23.3 ± 1.1°C), relative humidity of 50 ± 20 %, and unrestricted access to food and water. Cages contained a variety of perches and enrichment devices. Procedures were conducted with prior approval by the MIT Committee for Animal Care (CAC) and following veterinary guidelines. A list of marmosets used in this study and their ages is provided in Table S1. Marmosets (GD135 – 13+ years old, 10 individuals for snRNAseq, 4 individuals for FISH, and 2 individuals for AAV injection), were euthanized and brains harvested as previously described^7^. Marmosets were generated from a total of 15 breeding pairs. Briefly, animals were deeply sedated by intramuscular injection of ketamine (20–40 mg/kg) or alfaxalone (5–10 mg/kg), followed by intravenous injection of sodium pentobarbital (10–30 mg/kg). When the pedal with-drawal reflex was eliminated and/or the respiratory rate was diminished, animals were trans-cardially perfused with ice-cold sterile PBS. Whole brains were rapidly extracted into fresh PBS on ice.

After transporting the brain to the lab on wet ice, the brain was sectioned into coronal blocking cuts (slabs, 2–8mm in thickness) using a chilled custom-designed marmoset brain matrix^7^. Surgical tools were autoclaved and allowed to cool before use. All tools, the matrix, and the dissecting block were cleaned with RNase Zap^™^ wipes (ThermoFisher) prior to each dissection. Slabs were transferred to a pre-chilled dissecting block and regions were dissected using a marmoset atlas as reference^92^ (Table S24). The areas targeted for prefrontal cortex includes areas 8, 9, 10, 11, 47L, 14R, 46, 47, 13, 32, and 45; areas 6M, 6DC, 4c and 4ab for motor cortex; caudate and putamen for striatum, and all thalamic nuclei except posterior regions of the pulvinar and lateral geniculate nucleus for thalamus. Fetal and neonate brains were not dissected using the brain matrix due to their small size. Instead, the brains were hemi-sectioned, placed on a cooled dissecting block, and the prefrontal cortex and motor cortex (only for neonate, not dissected at GD135) were scooped from the surface of either hemisphere using anatomical landmarks. Two large (several mm) coronal slabs approximately spanning from the anterior beginning of the temporal lobe to its posterior end were cut using a razor blade, and the striatum and thalamus were dissected from the anterior and posterior slabs respectively (Fig. S4A-B). For one neonate replicate (21–197), the brain was frozen and stored at −80°C for several months, placed at −20°C overnight prior to the day of dissection, and thawed on ice prior to dissection. Dissected tissue was transferred to chilled 1.5mL microcentrifuge tubes, snap-frozen in liquid nitrogen, and stored at −80°C until nuclei isolation. Dissections began within 90 minutes of euthanasia and were performed in a median time of ~40 minutes (range 30–80 minutes).

### Mouse tissue harvest for snRNAseq.

Animal work was performed in accordance with protocols approved by MIT’s Committee on Animal Care and NIH guidelines. All postnatal mice were wild-type C57BL/6J originally obtained from Jackson Laboratories and bred in-house. Timed pregnant C57BL/6J females were either obtained from Jackson Laboratories to arrive between gestation day 11 and 15 or were impregnated in house by setting up overnight mating pairs with females in proestrus or estrus phase. Embryos were harvested at E18.5 (18 days after the plug date). Mice were housed in a facility with a light cycle running from 07:00 to 19:00, temperature 20–22.2°C, humidity 30–70%, and food and water available *ad libitum*. Postnatal mice were not derived from timed pregnant females. Instead, age was determined during regular pup checks by experienced researchers based on the Jax Mice Pup Appearance Chart. Thus, ages are approximate within +/− 0.5 days for P4 neonates, within +/−1 day for P14 early adolescents, within +/− 3 days for P32 juvenile mice and P90 young adult mice, and within +/−1 week for aged mice (90 weeks). Except for the P32 and aged time points, mice were obtained from different litters, and minimal replicate effects were observed in the snRNAseq data, suggesting adequate matching of developmental time points across replicates. A list of mice used in this study is provided in Table S1.

Non-neonate animals were acclimated to the lab space for at least 30 minutes prior to beginning euthanasia. Euthanasia took place between 9am-12pm to control for circadian rhythm effects, with a maximum of four animals processed per batch. Non-neonate animals were deeply anesthetized with isoflurane and decapitated. Heads were briefly submerged in liquid nitrogen for 3 seconds. Neonates were anesthetized via hypothermia and decapitated. Surgical tools were autoclaved and allowed to cool before use. All tools, brain mold, and the dissecting block were cleaned with RNase Zap^™^ wipes prior to each dissection. Brains were harvested and sagittally sectioned at 1mm thickness for a total of 2 mm from the midline for either hemisphere (total of four ~1mm slices), on a brain mold using chilled razor blades. For P4 animals, two ~2mm sections from the midline were used. Tissue was dissected from slices on a chilled dissecting block exposed to room air, using a dissecting microscope at 1.6X magnification. Regions of interest were identified using the Allen Institute reference brain atlas at the appropriate time point. Dissected tissue was placed in cooled 1.5mL microcentrifuge tubes and spun down in a tabletop mini centrifuge prior to snap freezing in liquid nitrogen before storage at −80°C until nuclei isolation. 2–3 neonates were pooled in each tube. Time from decapitation to snap freezing ranged from 7–13 minutes per animal. Samples from at least 3 mice (at least 1 female) are represented at each developmental time point (except 90 weeks) and for each brain region. However, due to failures during microdissection, nuclei isolation, and 10x Genomics chip running, not all biological replicates are balanced across brain regions (e.g., some replicates have only one brain region present).

For embryonic brain microdissection, the pregnant dam was deeply anesthetized with an overdose of isoflurane, decapitated, and placed on a cooled dissecting block. The abdomen was opened and placentas were removed from the abdominal cavity. Embryos were harvested from the placenta and rapidly decapitated one-by-one. Heads were frozen on metal disks over dry ice for 5–10 minutes until frozen solid, stored at −20°C for 1.5 hours prior to microdissection. Heads were cut approximately in half using a small mouse brain mold and placed on a dry-ice cooled metal platform. Regions of interest were dissected using a tissue punch (1.27mm Ted Pella MilTex Biopsy Punch with Plunger, 15110–10) to extract tissue from most medial surface on either hemisphere. 2–3 embryos were pooled in each tube. The other dissection procedures were the same as described above. Dissections were performed in less than 20 minutes per set of tubes from decapitation to snap freezing.

### Nuclei isolation and single-nucleus RNA sequencing.

Nuclei were extracted from frozen tissue using the 10x Genomics Chromium Nuclei Isolation Kit (Protocol CG000505, Rev A). Manufacturer instructions were followed with the following notable exceptions: 1) Most samples were dissected and frozen directly in Sample Dissociation Tubes (omitting step e), 2) Total lysis time was decreased to 10–14 total minutes of incubation in the lysis buffer (longer for marmoset and larger tissue chunks) from when lysis buffer was first added to the first sample (effectively shortening protocol step h) before proceeding to step i; 3) Tissue mass was larger than the 45mg upper limit recommendation for some marmoset samples; 4) Lysis buffer was supplemented with Roche Protector RNase inhibitor at 0.2U/uL, and 5) if no pellet was visible following any centrifugation steps, ~200uL or less of supernatant was retained for samples with a visible pellet or debris. For the final resuspension step (step s), if no pellet was visible, less than ~40–100uL of supernatant was retained at the bottom of the tube and no additional volume was added prior to nuclei counting. To avoid large clogs, large chunks of marmoset tissue were split in half and processed in parallel for nuclei isolation, and re-pooled prior to 10x Chromium chip loading. For nuclei isolation, a maximum of 4 samples were processed in series by a single researcher in a given preparation (usually 8 samples total, with 2 researchers in parallel). For tissue dissociation (step f), samples with a small amount of tissue (~20mg or less) were processed (transferred to wet ice, coated with 200uL lysis buffer, and dissociated with pestle) one at a time. For dissociating larger amounts of tissue (larger than ~20mg) that required some thawing before pestle dissociation, samples were transferred to wet ice and coated with 200–300uL of lysis buffer in parallel, and then homogenized with the pestle one at a time.

Nuclei concentration was quantified by staining suspensions with DAPI, loading on a C-Chip hemocytometer, imaging on a fluorescence microscope, and using the Fiji 3D Object Counter plugin to automatically quantify the number of nuclei within 4 large grid squares (0.8uL of volume). Debris was assessed by comparing the signal in bright field to the signal in DAPI and nuclei quality was assessed by examining DAPI-stained nuclei at 40–60x magnification prior to starting the 10x Genomics Chromium snRNAseq protocol. Nuclei suspensions with an unacceptable amount of debris (i.e., large clumps in bright field that were not DAPI+) and/or blebbing (i.e., with most nuclear membranes appearing substantially disrupted) were discarded. Because marmoset tissue is precious, a higher level of debris and/or blebbing was tolerated for marmoset nuclei suspensions. Nuclei suspensions were diluted to a target concentration of 1,000 nuclei/uL for 10x Genomics Chromium chip loading.

snRNAseq libraries were prepared using 10x Genomics Chromium Next GEM Single Cell 3ʹ Reagent Kits v3.1 (Protocol CG000315 Rev C or Rev D) following manufacturer instructions. Time from tissue lysis to 10x Chromium chip loading averaged ~2 hours. Whenever possible, channels were loaded with enough nuclei suspension to recover a target of 10,000 nuclei. Initial cDNA amplification was performed using 13 PCR cycles and Sample Index PCR was performed using 11–12 PCR cycles. Amplified cDNA (product of protocol step 2) and libraries (product of protocol step 3) were quantified and quality-checked using both Qubit (HS dsDNA Assay) and a Fragment Analyzer. Libraries were pooled and sequenced on an Illumina NovaSeq at the Broad Genomics Platform. Data are available for download at: https://data.nemoarchive.org/biccn/grant/u01_feng/feng/transcriptome/sncell/10x_v3.1/.

### Read alignment.

Reads were aligned to an optimized mouse reference genome based on the mouse GRCm38 primary sequence assembly (version 2)^93^ or or a modified marmoset mCalja1.2.pat.X assembly (https://www.ncbi.nlm.nih.gov/datasets/genome/GCF_011100555.1/) provided courtesy of Michael DeBerardine and Fenna Krienen (Princeton Neuroscience Institute) in which mitochondrial genes from CM021961.1 (https://www.ncbi.nlm.nih.gov/nuccore/1820101357/) were annotated using MITOS2 (https://doi.org/10.1093/nar/gkz833). The marmoset reference genome was generated from the .fasta and .gtf files using Cell Ranger “mkref” v7.1.0 using default parameters without filtering for any gene/transcript biotype. 10x Genomics Cell Ranger software version 7.1 was used for alignment and counting via the 10x Genomics Cloud Analysis platform. For samples that were sequenced across several library pools, fastq files were grouped prior to alignment to create one cell-by-gene (cell x gene) counts matrix per sample. CellBender (v0.2.0) remove-background^94^ was used to remove ambient RNA and call nuclei with default parameters and expected_cells = 10,000, total-droplets-included = 40,000 and the –cuda flag. CellBender-cleaned cell x gene matrix .h5 files were read into Python in the anndata^95^ format using a custom function written by Stephen Fleming (https://github.com/broadinstitute/CellBender/issues/57).

### Calculation of sequencing coverage statistics.

To determine whether we achieved our target of 40,000 sense reads per nucleus and calculate the sequencing coverage statistics shown in Fig. S1 and Table S25, we ran a light quality control on CellBender-cleaned cell x gene matrices. Briefly, nuclei with fewer than 1,000 unique molecular identifiers (UMIs) and fewer than 800 genes expressed were removed, as were genes with nonzero expression in fewer than 10 cells. We note these cutoffs are more stringent than what we used for preprocessing (see “snRNAseq data preprocessing and quality control” section), to account for the lack of doublet removal and additional manual curation that is much more time consuming. Nuclei with greater than 4% of reads aligning to the mitochondrial genome (prefix “mt-” for mouse or “MT-” for marmoset) were removed.

### Sex determination in mouse and marmoset fetal and neonate samples.

Because 2–3 mouse E18.5 brain regions were pooled into a single tube for generating the nuclei suspension without sex determination, we do not have metadata about sex for these samples. Instead, we performed sex assignment on a per-nucleus basis after snRNAseq based on the expression of Y-chromosome genes. Specifically, if a nucleus had *Zfy1*, *Zfy2, Usp9y*, *Uty*, *Eif2s3y*, *Kdm5d*, or *Ddx3y* expression above 2 log counts per million, it was assigned male sex. This resulted in 41.37% male nuclei for the E18.5 samples, likely an underestimate due to dropout.

Marmoset GD135 donors and one neonate donor (21–197) did not have their sex determined anatomically prior to euthanasia. To determine their sex, we performed PCR-based sex genotyping on either skin or brain tissue. Briefly, DNA was extracted from tissue using the NucleoSpin Tissue kit (Macherey-Nagel) and eluted in nuclease-free water. We genotyped for *ZfX/Y*^96^ and *SRY*^97^ using the following PCR primers (from 5’ to 3’):

- ZfX/Y Forward (modified from the original publication^96^): CTGTGCATAACTTTGTTCCTG
- ZfX/Y Reverse (modified from the original publication^96^): CAGTTGCCTTTGTCATCATC
- SRY Forward: TACAGGCCATGCACAGAGAG
- SRY Reverse: CTAGCGGGTGTTCCATTGTT

And ran the following protocol on a thermocycler:

- 94°C - 2 min
- 98°C - 10 sec
- 58°C - 30 sec
- 68°C - 40 sec
- 35 or 40 rxns (34x or 39x)
- 68°C - 3 min
- 4°C - Infinite

We then digested 10uL of the ZFX/Y product using the DdeI and MseI enzymes in separate reactions (New England Biolabs). We were able to see clear separation of bands on a 2% agarose gel run at 135V for 40–45 min. Digesting the ZfX/Y PCR product with DdeI will yield double bands if the animal is XX and triple bands if the animal is XY. Digesting the ZfX/Y PCR product with MseI, which can only cut the ZfY band, will show smaller bands below the ~500bp PCR product for XY animals and a single band for XX animals. An *SRY* band of around 218bp indicates male sex. Our predicted sexes also matched *SRY* gene expression after snRNAseq.

### snRNAseq data preprocessing and quality control.

All scripts used for preprocessing and downstream analyses are available on GitHub at https://github.com/Feng-Lab-MIT/AstrocyteHeterogeneity. Preprocessing was conducted on a species-wide, cross-age, cross-region basis. Filtered counts matrices were pre-processed using scanpy^98^. Briefly, nuclei with fewer than 800 unique molecular identifiers (UMIs) and fewer than 500 genes expressed were removed, as were genes with nonzero expression in fewer than 10 cells. Nuclei with more than 4% of counts annotated as mitochondrial genes (prefix “MT-” in marmoset or “mt-” in mouse) were removed. Counts were normalized to 1 million per cell and log-transformed using scanpy’s “log1p” function. Highly variable genes (HVGs) were identified from raw counts on a per-batch basis using scanpy’s “seurat_v3” method with 4,000 top genes. HVGs present in less than 10% of batches or of mitochondrial origin (prefix “MT-” in marmoset or “mt-” in mouse) were removed. We used the scanpy-based single cell variational inference (scVI^99^) package to create and train a variational autoencoder on a subset of the cell x gene matrix with highly variable genes only with the following parameters: batch_key corresponding to 10x genomics reaction, raw counts layer, “gene-batch” dispersion, and training with GPU. The resulting nonlinear embedding was used to create a neighborhood graph for clustering and calculate UMAP^100^ coordinates with the scanpy “sc.pp.neighbors” and “sc.tl.umap” functions. scVI and the scvi-tools package were the basis for many downstream analyses. We used Solo, an automated doublet removal package^101^ based on the scVI model, to calculate doublet scores for each nucleus on a per-batch basis. For the mouse data, one nucleus was removed from the “Exp074_mmP35_2D” 10x reaction to circumvent a known bug in the Solo package (https://discourse.scverse.org/t/solo-scvi-train-error-related-to-batch-size/1591/2). Doublet/singlet thresholds were determined manually by examining the doublet-vs. singlet-score scatter plot and a predicted doublet rate of 13–15%, a conservative estimate based on 10x Genomics’ predicted ~8% for a target recovery of 10,000 nuclei. We decided to use this manual threshold to prevent removal of developing cells that are more likely to be flagged doublets automatically.

### Global (species-wide, cross-age, cross-region) and annotation.

After automated doublet removal, highly variable genes were re-calculated and the scVI model was re-trained. Leiden clustering was performed with a resolution of 1. Top differentially expressed genes in each cluster (i.e., putative marker genes) were identified using scanpy’s rank_genes_groups function with log-normalized counts and the Wilcoxon rank-sum method. Top marker genes, expression of known marker genes, and dendrograms were used to annotate clusters per the following convention: [Cell class]_[Excit or Inh*]_[region*]_[cortical layer*]_[age*]_[known marker*]_[first rank_genes_group marker]-[second rank_genes_group marker*], where the asterisked attributes were used variably, as applicable. Low-quality clusters were manually identified and removed, highly variable genes re-calculated, the scVI model re-trained, and clustering and annotation processes were repeated. Remaining doublet clusters were manually removed, and the neighborhood space and UMAP coordinates were recalculated. Neuronal annotations were subsequently refined based on predicted mapping to the Allen Brain Cell Atlas using MapMyCells^36^ (see Alignment to Allen Brain Cell Atlas with MapMyCells section below).

Adult (29–32 month) marmoset data used in this study was generated previously^7^, and includes data from 4 donors. CellBender background-removed cell x gene matrices for the four regions of interest (annotated as “pfc”, “m1”, “striatum”, and “thal”) were preprocessed as described above, except that mitochondrial genes were not annotated and therefore not used for quality control. Adult marmoset cell x gene matrices were randomly downsampled to 40,000 nuclei per region to match the approximate number of nuclei in each age-region combination in the developmental dataset. Of note, the adult marmoset snRNAseq reads were aligned to the cj1700 transcriptome, which lacks mitochondrial genome annotation (https://www.ncbi.nlm.nih.gov/datasets/genome/GCF_009663435.1/). 22,582 genes overlapped between the mCalJa1.2.pat.X (developmental and aged data, 31,308 genes) and cj1700 (adult data, 27,304 genes) reference-aligned datasets. Data from GD135, neonate, 7-month, 14-month, and aged timepoints were integrated with the downsampled adult (29–32 month) data using scVI using only highly variable genes, clustered, and annotated and described above. Clusters with the vast majority or all nuclei derived from adult (29–32 month) marmoset data were removed, as they likely derive from differences in dissection strategies between the two studies. We also removed a small subcluster of 233 adult astrocytes that clustered with immature astrocytes, because they derived primarily from a single adult replicate and were not found in other donors 14 months and older. Neuronal annotations were subsequently refined based on predicted mapping to the Allen Brain Cell Atlas using MapMyCells^36^ (see Alignment to Allen Brain Cell Atlas with MapMyCells section below). In designing and implementing downstream analyses (e.g. compositional, pseudotime, and cell-cell interaction analyses), we relied heavily on the Single Cell Best Practices e-book (https://www.sc-best-practices.org/)^102^.

### Alignment to Allen Brain Cell Atlas (ABCA) with MapMyCells^36^.

Both mouse and marmoset mature neurons (GABAergic, glutamatergic, and mixed from mouse P14 and above or marmoset neonate and above) or astrocytes were subsetted from the larger cell x gene matrix of each species by age and region, with some metadata removed to shrink the file size. To use the maximum number of genes available for each marmoset time point, pre-integrated adult or developmental gene counts (that is, with ~27,000 genes for 29–32 month marmoset data and ~31,000 genes for developmental and aged data) were used as input. Per the MapMyCell input requirements (https://portal.brain-map.org/explore/file-requirements-and-limits), cell x gene matrix entries were set to raw counts and NCBI gene symbols were converted to mouse Ensembl gene IDs using g:Profiler’s g:Convert^103^. Marmoset NCBI gene symbols were converted to mouse NCBI gene symbols using a table downloaded from Ensembl BioMart (https://useast.ensembl.org/info/data/index.html)^104^, available in Table S26. If no match was found in the table, the marmoset gene name was converted to sentence case. Due to non-uniqueness after converting marmoset IDs to mouse, *Bex2* was removed from the adult marmoset neuron cell x gene matrix, while *Cstl* was removed from the developmental and aged marmoset neuron cell x gene matrices, before mapping to Ensembl IDs. ~25,300 mouse and ~14,800 marmoset genes were successfully mapped to Ensembl IDs, and the others were excluded from MapMyCells analysis. These smaller (<2Gb) cell x gene matrices were uploaded to the Allen Brain Maps’s MapMyCells web portal (https://knowledge.brain-map.org/mapmycells/process/) and aligned to the 10x Whole Mouse Brain (CCN20230722) reference taxonomy^1^ with the hierarchical mapping algorithm. Using the output of MapMyCells, which is a “class”, “subclass”, “supertype”, and “cluster” assignment for each barcode, we examined the most abundant (as the percentage of our cells in each of our clusters mapping to that subclass) ABCA subclass (about the same taxonomic rank) for each of our cross-region, cross-age, within-species embedding Leiden clusters. Broadly, our annotations were identical or highly similar to the most abundant ABCA mappings. When our annotation disagreed with or was not as specific as the most abundant ABCA subclass mapping, and we were not confident in our annotation, we updated the annotation based on the most abundant ABCA subclass (e.g., comprising over 60% of the cells in the cluster for mice, or over 20% for marmoset). For clusters that had a more uniformly distributed mapping onto ABCA subclasses (e.g., less than 10% mapping onto each subclass), we examined several of the top mapping clusters and their anatomical locations (using the web resource from ref^1^ available at https://knowledge.brain-map.org/data/5C0201JSVE04WY6DMVC/summary), and if they were in the same taxonomic or anatomical neighborhood, annotated our cluster accordingly. In a few cases, such as for marmoset thalamic neurons mapping to a midbrain ABCA population and immature astrocytes mapping to the Allen olfactory bulb/immature neuron subtype, we did not adopt the ABCA subclass label. The class, subclass, supertype, and cluster mappings for each of our leiden clusters (as proportion of cells in that cluster mapping to each ABCA taxonomic rank) for both species are provided in Table S4.

### Compositional analysis with scCODA^37^.

The proportional breakdown of each leiden cluster by developmental time point (age), brain region, and sex are provided in Table S3 (assigned brain region) and displayed in Fig. S3 (dissected brain region). To more quantitatively assess cell type composition changes across these variables, we used the scanpy-based single-cell compositional data analysis (scCODA)^37^ (https://github.com/theislab/scCODA), which implements a Bayesian model of cell type counts to address the issue of low sample sizes in snRNAseq data. For this analysis, we merged the PFC and MO into one “cortex” assignment, due to their high degree of similarity in cell type composition. Per the tutorial in the Single Cell Best Practices e-book (https://www.sc-best-practices.org/)^102^ Section 17.4, we generated an scCODA model of type “cell_level” with the cell type identifier as either “leiden” (cluster) or “cell_type”, sample identifier as “10x_batch” and “sex” and age, region, sex, and replicate as covariate observation. We ran the model with the formula “region + age + sex”, automatic selection of reference cell type for leiden-level analysis and Mural (marmoset) or Astrocyte (mouse) as the reference cell type for cell type-level analysis, and default false discovery rate of 0.05. The “final parameter”, which is a boolean value that indicates whether or not there is a significant effect of age, region, or sex on the composition of each cell type and cluster for both mouse and marmoset are provided in Tables S5-8.

### Region reassignment of cross-contaminant nuclei in fetal and neonate marmoset.

We observed a modest amount of cross-region contamination, particularly between striatum and thalamus in our late embryonic and neonate samples, which is not unexpected given our coarser dissection strategy for these regions (Fig. S4-5). To reduce the effect of this cross-contamination on downstream analyses, we reassigned the region annotation for neurons, astrocytes, and OPCs (the most strongly region-segregated cell types on which we focused our analysis) at these ages. We subsetted the cell type of interest from the cross-age, cross-region integrated cell x gene matrix, re-computed neighbors using the scVI latent space, and recomputed the UMAP space for each cell type. We then assigned each nucleus as either *FOXG1*+ or *FOXG1-* based on an expression threshold of 4 logCPM, and performed Leiden clustering at low resolution (0.3). Any GD135/neonate astrocyte nucleus from the thalamic dissection that co-clustered with primarily telencephalic astrocytes was assigned striatum if it mapped to the telencephalic astrocyte ABCA subclass. Any GD135/neonate astrocyte from the striatum dissection in the same Leiden cluster was the thalamic nuclei was assigned thalamus if it was *FOXG1*- and it mapped to the non-telencephalic ABCA subclass. Any GD135/neonate astrocyte from the thalamic dissection that did not meet the first condition but was *FOXG1*+ or mapped to the telencephalic ABCA subclass was assigned striatum. Any GD135/neonate GABAergic neurons from the striatum clustering with thalamic GABAergic neurons (either TRN or midbrain-derived) was assigned thalamus, any thalamic neuron clustering with medium spiny neurons was assigned striatum, and any *FOXG1*+ nucleus from the thalamic dissection was assigned striatum. Any GD135/neonate neurons clustering with thalamic glutamatergic neurons were assigned thalamus, and any GD135 glutamatergic neurons not clustering with thalamic glutamatergic neurons were assigned PFC. Since OPCs did not cluster by region, we did not reassign region based on clustering, but instead, used only *FOXG1*: any OPCs from the thalamic dissection that were *FOXG1*+ were assigned striatum. The resulting assigned region annotation is saved in the “region” .obs variable of the annotated data files, while the original region is saved in “region_dissected”.

### Region reassignment of cross-contaminant nuclei in mouse.

Cross-region contamination was higher in the E18.5 and P4 mouse timepoints, but present in all ages, primarily between striatum and thalamus (Fig. S5). Similarly to marmoset, we subsetted the cell type of interest from the cross-age, cross-region integrated cell x gene matrix, re-computed neighbors using the scVI latent space, and recomputed the UMAP space for each cell type. We then assigned each nucleus as either *Foxg1*+ or *Foxg1-* based on an expression threshold of 4 logCPM, and performed Leiden clustering at low resolution (0.3). Nuclei in immature astrocyte clusters composed of mixed regions were reassigned based on *Foxg1* expression or assignment in ABCA subclass by MapMyCells. Astrocytes from the thalamic dissection in these mixed region clusters were reassigned as striatum if they were *Foxg1*+ or had the subclass of “Astro-TE-NN”. Immature clusters exhibiting more clear telencephalic and diencephalic divisions (mostly from P4) were manually reassigned, either from thalamus to striatum, or striatum to thalamus, to match the predominant region of origin for that cluster. All thalamic dissected astrocytes in the predominantly telencephalic and P4 cluster were manually reassigned to striatum. For the primarily thalamic and P4 cluster, we reassigned all cells to match their ABCA subclass (all “Astro-TE NN” being labeled striatum and all “Astro-NT NN” being labeled thalamus) and any cells that were *Foxg1*+ were also labeled striatum. Because this cluster contained *Foxg1*+ cells, we did not reassign all nuclei in it to thalamus. For the mature astrocytes, thalamic nuclei were reassigned to striatum if they clustered with telencephalic astrocytes and were assigned the “Astro-TE NN” ABCA subclass, and vice-versa for striatal astrocyte nuclei clustering with thalamic astrocyte nuclei. For OPCs, thalamic dissected cells were reassigned striatum if they were *Foxg1*+. For excitatory neurons, any striatum dissected cells were reassigned to cortex or thalamus based on the predominant region of the nuclei in their cluster. Any thalamic dissected excitatory neurons were reassigned as cortex if they clustered with cortical excitatory neurons and were *Foxg1*+. Finally, for inhibitory neurons, any thalamic dissected cells outside of the TRN cluster in E18.5 or P4 were reassigned either cortex or striatum based on the prominent region of the cluster they grouped with, and any cell at E18.5 or P4 in the excitatory TRN cluster were reassigned as thalamus. At all other time points, any thalamic excitatory neuron that was *Foxg1*+ was reassigned as cortex. The resulting assigned region annotation is saved in the “region” .obs variable of the annotated data files, while the original region is saved in “region_dissected”. 4.4% of astrocytes, 0.2% of OPCs, 2.6% of excitatory neurons, and 3.8% of inhibitory neurons were reassigned from their dissected region. There was also a batch of 90 weeks nuclei that was mislabeled prior to sequencing as striatum, but reassigned to cortex due to almost all cells aligning to cortical clusters within our data and to the ABCA clusters (see “Notes” column in Table S1).

### Calculation of rDEGs, aDEGs, and sDEGs using a pseudobulk method.

Regional differentially expressed genes (rDEGs, Fig. 2) were calculated as previously described^7^ with some modifications. rDEGs were calculated for each individual developmental time point, cell type, and species separately. To use the maximum number of genes available for each marmoset time point, pre-integrated adult or developmental gene counts (that is, with ~27,000 genes for adult marmoset data and ~31,000 genes for developmental and aged data) were used for rDEG calculation. We first created per-region metacells for each cell type (astrocyte, OPC, GABAergic neuron, and glutamatergic neuron) by averaging the raw counts of all cells of each cell type per region and per replicate. If a metacell of one region, cell-type, and replicate combination had fewer than 50 cells, it was omitted. Because they had few rDEGs between them, motor and prefrontal cortices were grouped for rDEG analysis. Metacell counts were normalized to 100,000 counts total and log10-transformed. We required rDEG candidates have at least 10 transcripts per 100,000 in at least one metacell per region and be expressed in at least 33% of nuclei in the metacell. 33% was chosen to require a significant portion of cells in a metacell to express the gene, but also be low enough to account for dropout^105^. Genes with >10^0.5^ (3.16) fold-change (logFC) expression in the same cell type between two regions (pairwise) were considered rDEGs. For marmoset, we restricted this analysis to the 2 adult donors (bi005 and bi007, from our previous study^7^) that were represented in each regions, and we required that rDEGs be found in the same region pair in both individuals at all ages. Because mouse replicates were not perfectly balanced across age-region combinations (e.g., some mouse donors only provided tissue for one or two regions), we merged all mouse replicates of the same age and region into one metacell, to accommodate the metacell code that was written to draw comparisons within each replicate separately.

To plot rDEG expression heatmaps as in Figs. 2–3B, we plotted the rDEG lists from fetal, early adolescent, and adult time points, ordered first by the age the gene was differentially expressed and second by the region(s) that the gene that was more highly expressed in, including repeats if an rDEG was detected at multiple time points. Marmoset rDEGs were only included if they were present in both replicates of a given time point, while mouse rDEGs did not have a replicate restriction.Because of the high heterogeneity in the mouse striatal astrocytes due to the multiple immature populations, these cells were reordered to place cells of similar populations together in the heatmap. To do so, striatal astrocytes were clustered at a low leiden resolution (0.1) and these clusters were ordered using a combination of the pseudotemporal ordering and the ages present in the cluster. A similar logic was applied to order marmoset striatal nuclei in the rDEG expression heatmaps.

Raster plots underneath the rDEG expression heatmaps were generated as separate figures using custom Python scripts with the help of ChatGPT 4.0. In brief, we created a matrix encoding the age and region(s) for which each gene was an rDEG, and plotted a line of the corresponding color in the corresponding raster row. These separate figures were manually aligned below the scanpy-generated rDEG expression heatmaps to the best of our abilities, but the x-axes may not be exactly aligned. To create the UpSet^106^ plots in Figs. 2–3C-D, Fig. 5C-D, Fig. 6E, and Fig. S14B-C, we used the package UpSetPlot (https://github.com/jnothman/UpSetPlot). The “active” dots were manually colored according to the group’s color scheme for clarity.

The pairwise region rDEG scatter plots in Fig. S6 were generated as follows. First, we recalculated rDEGs for each species within each region pair using the same metacell method described above, with one modification to allow more lowly expressed genes to pass the filter: requiring a gene be expressed by a minimum of 33% of cells in *any* region of either cortex, striatum, or thalamus. We then plotted the log fold-changes (calculated as described above) for each gene in (x,y) with x being the logFC value for one region pair (e.g. cortex-striatum), and y being the logFC value for another region pair (e.g. cortex-thalamus). The sign on the logFC value was determine with respect to the region shared across the pairwise comparisons (e.g. cortex). Pearson’s correlation coefficient, r, was calculated using the scipy stats package (https://docs.scipy.org/doc/scipy/reference/generated/scipy.stats.pearsonr.html). A gene was marked as rDEG in one region if it had greater than 0.5 magnitude logFC in that region, or both if it had greater than 0.5 magnitude logFC in both.

aDEGs (Fig. 5) were calculated in a similar manner, except for that the metacell axis was age instead of region, and that no cross-replicate consistency was imposed for either species, because no biological replicate provided tissue for multiple developmental time points. For aDEG and rDEG calculation in marmoset, lateral septal GABAergic neurons and putative hippocampal Cajal-Retzius neurons were removed, as we could not assign their region to either cortex, striatum, or thalamus, before metacell generation. Similarly for mouse, we omitted lateral septal GABAergic neurons and glutamatergic neurons from the anterior olfactory nucleus before aDEG and rDEG calculation. To calculate species differentially expressed genes (sDEGs, Fig. 6), we first combined downsampled mouse and marmoset datasets of all developmental time points and brain regions using the intersection of genes with 1:1 orthologs converted to mouse gene IDs using Table S26. If no match was found in the table, the marmoset gene name was converted to sentence case. We then created metacells of each supercluster for each species, where species was the metacell axis, and compared gene expression between species within each supercluster. As for aDEGs, we did not impose a cross-replicate requirement on sDEGs for either species because no replicate can be a member of both species.

We calculated the overlap between sDEGs and aDEGs between mouse and marmoset (Fig. 6B-C) by converting marmoset gene names to mouse gene names based on 1:1 orthologs using Table S26 (as before, if no match was found in the table, the marmoset gene name was converted to sentence case) and taking the intersection between the two lists. To calculate whether a marmoset astrocyte rDEG was more likely to be a mouse astrocyte rDEG and vice-versa, we performed a Chi-square test using scipy stats’s “chisquare” test (https://docs.scipy.org/doc/scipy/reference/generated/scipy.stats.chisquare.html). The expected (“f_exp”) proportions of non-rDEGs and rDEGs were calculated as the number of non-rDEGs and rDEGs divided by the number of 1:1 species orthologs, while the observed (“f_obs”) proportions of non-rDEGs and rDEGs were calculated as the number of non-shared rDEGs divided by the total number of rDEGs for a given species and the number of species-shared rDEGs divided by the total number of rDEGs for that species.

### Astrocyte subclustering within each brain region.

We conducted sub-clustering for subsetted astrocytes from each brain region for each species separately (Fig. S11). Using the subsetted cell x gene matrix for each region (all cell types), we removed small 10x Chromium batches with smaller than 200 cells (from reassigned regions), highly variable genes were recalculated (minimum number of batches equal to the floor of the total number of 10x Chromium batches divided by 10, to avoid batch-specific differentially expressed genes while including region-specific variation), the scVI model re-trained, and the neighborhood graph and UMAP coordinates re-calculated on subsetted astrocytes as described above. For mouse, highly variable genes were recalculated per region based on the original dissected region rather than the reassigned region due to a known issue with small batch sizes with the sc.pp.highly_variable_genes function. Our sub-clustering procedure was inspired by earlier methods^65^. Scanpy’s “tl.leiden” function was used to identify clusters over a range of decreasing resolution parameters, purposefully starting with an intentionally high resolution that led to over-clustering. At each resolution, the minimum number of pairwise and one-versus-rest (where one group is compared to a metacell of all other groups combined) differentially expressed genes (DEGs) for each cluster and pair of clusters was calculated using the metacell method described above for rDEGs/aDEGs/sDEGs, with metacells calculated for each cluster, not incorporating replicate information. We first conducted a course resolution scan with increments of 0.05, followed by a fine-grained resolution scan in the target resolution range with increments of 0.01. We chose a clustering resolution that resulted in at least 3, but often 10 or more, pairwise and one-vs-rest DEGs for each subcluster and subcluster pair. Mouse thalamic and cortical astrocytes had quality issues that resulted in the mature protoplasmic astrocyte subclusters splitting into multiple clusters with almost only mitochondrial pairwise DEGs. As a result, these clusters were manually combined (merging to a maximum of 5 clusters into 1) to preserve heterogeneity from higher resolutions in other clusters. The resulting pairwise DEGs numbered 5 or above for each cluster.

### Pathway analysis with WebGestalt.

We employed WebGestalt 2024^49,50^ (https://www.webgestalt.org/) over-representation analysis (ORA) for pathway analysis. For the reference gene set, we used either all genes present in the final mouse counts matrix (“adata.var_names”), all genes present in the fetal, neonate, 7-month, 14-month, and aged marmoset data (generated in the current study with the mCalja1.2.Pat.X reference), or all genes present in the adult (29–32 month) marmoset data (generated previously^7^ and aligned to cj1700). We used human as the host species for marmoset, mouse as the mouse species for mouse, the Gene Ontology (GO) Biological Process^107,108^ noRedundant and KEGG^109^ pathway functional databases, weighted set cover redundancy reduction for pathway display, and default advanced WebGestalt settings. Because differentially expressed genes were calculated in a pairwise manner, pathway analyses were performed on genes differentially expressed in both directions (i.e., either up- or down-regulated, as opposed to unidirectionally). Lollipop plots were generated from “Description”, “Ratio”, and “FDR” columns of the weighted set cover redundancy-reduced pathway table using custom Python scripts with the assistance of ChatGPT 4.0.

### Cross-species integration with scANVI^63^.

scANVI^63^ is a semi-supervised variational autoencoder variant of the previously described scVI model that utilizes cell type label information in its latent space. We used a random downsample of 20,000 cells from any age or region for each species. Next, we added a less granular “supercluster” annotation to each cell based on its leiden cluster annotation. For example, two marmoset cortical excitatory neuron L6IT clusters were combined into one supercluster. In some cases, the leiden cluster to supercluster mapping was one:to:one, as for OPCs, lateral septal inhibitory neurons, diencephalic and telencephalic astrocytes, and others. To create the embedding space, we used a small subset of highly variable genes for cross-species integration that was inspired by an earlier approach^65^. Briefly, we converted the marmoset gene names to their mouse orthologs and subsetted both datasets to the intersection of shared genes. Next, we took the top 50 most highly expressed genes in each supercluster (as determined by a Wilcoxon rank-sum test in scanpy) in each species separately, then used the intersection of those lists as the highly variable genes entered into the model. We created a scANVI model using the superclusters as the labels key, the dispersion set to “gene-batch”, and calculated on the raw counts layer. Finally, we calculated the neighborhood graph and UMAP using scanpy’s previously mentioned functions.

### Cross-species integration with SATURN^64^.

SATURN is a deep learning method used to integrate cells from multiple species in the same low-dimensional space. It utilizes the ESM2^110^ language model and reference genomes from Ensembl (for marmoset: https://useast.ensembl.org/Callithrix_jacchus/Info/Index, https://useast.ensembl.org/Mus_musculus/Info/Index for mouse, as in the original publication) to generate protein embeddings that predict similarity of genes across species. The marmoset protein embedding space was generated by the lead author of the SATURN study and is linked at https://github.com/snap-stanford/SATURN/issues/19, and the mouse protein embedding is available at http://snap.stanford.edu/saturn/data/protein_embeddings.tar.gz. SATURN uses a combination of cell type annotations from the user and the protein embeddings to create “macrogenes”, groups of genes that are predicted to be “functionally related” and “coexpressed across species”^64^. It then uses a weakly supervised autoencoder to refine the macrogene space by using a triplet loss function that incorporates within-species cell type annotations. Before running SATURN, we first randomly downsampled the annotated cell x gene matrices for both species to 100,000 nuclei total to reduce computational burden. We provided SATURN with a mapping between mouse and marmoset supercluster names, which were identical except for 2–3 unique superclusters per species. We trained the SATURN model as instructed in the tutorial (https://github.com/snap-stanford/SATURN/blob/main/Vignettes/frog_zebrafish_embryogenesis/Train%20SATURN.ipynb), using raw (not normalized) counts, 2,000 macrogenes, 8,000 highly-variable genes, and our supercluster mapping as the cell type mapping file. The resulting SATURN embedding in UMAP space was used to generate the plots in Fig. S15.

We performed SATURN cross-species integration on the same downsampled, supercluster-annotated cell x gene expression matrix as for our scANVI approach (with the exception of L5IT cortical excitatory neurons labeled separately for marmoset). By visual inspection, the species-integrated UMAP calculated from the SATURN embedding yielded similar results as our scANVI approach: broad conservation with a few species-specific clusters as described above (Fig. S15D). However, because SATURN does not explicitly rely on cell type annotation to group cells from different species, it was able to merge cortical glutamatergic L5IT supercluster mouse neurons with the corresponding population in marmoset (Fig. S15D, dark green cluster with the asterisked label), despite the fact that L5IT mouse neurons were not separately annotated as such, but grouped with L4/5IT cortical glutamatergic neurons. The scANVI approach did not result in this merging unless we grouped L5IT and L4/5IT neuron superclusters in both species, which is the approach we adopted for the results shown in Fig. 6. However, the relative position of superclusters in the SATURN UMAP seems to be less meaningful than for the scVI/scANVI embeddings: related neuronal clusters are no longer adjacent in UMAP space, OPCs and MOLs are far away from one another, and microglia were well integrated, despite species differences apparent in the scANVI integration and past studies suggesting they should be species-divergent^66,111^. Closer examination of the SATURN-integrated astrocytes illustrates concordant results with the scANVI integration: telencephalic marmoset and mouse astrocytes were well-integrated, diencephalic astrocytes were more separated, and immature astrocytes were almost completely separated (Fig. S15E).

### Cell-cell communication analysis.

We performed cell-cell communication analysis on a per-region (prefrontal and motor cortex pooled), per-age, per-species basis in Python using CellPhoneDB^112^ via Liana^113^, a scanpy-friendly package that integrates several cell-cell interaction inference methods. To avoid spurious findings due to differences in cluster proportion (i.e., bias towards or away from rare cell types), we first randomly downsampled all clusters to a maximum of 1,000 nuclei and dropped all clusters with fewer than 100 (thalamus and cortex) or 70 (striatum, to avoid dropping the sparse *CHAT*+ neuron cluster) nuclei. As recommended in the Liana documentation, we ran CellPhone DB on the log1p-transformed normalized counts matrix, grouping by leiden cluster, using the “consensus” resource for marmoset and the “mouse consensus” resource for mouse, and requiring that 33% of nuclei in a cluster express a gene at nonzero levels for it to be considered for ligand-receptor enrichment. CellPhoneDB generated two outputs of interest: 1) “lr_means”, a measure of interaction magnitude, and is simply the mean expression of the ligand in the source cluster averaged with the mean expression of the receptor in the target cluster, and 2) the permutation-based p-value, a measure of interaction specificity. Briefly, the specificity p-value for a ligand-receptor pair is calculated as the proportion of null distribution (generated by randomly permuting the cluster labels of all cells) means that are greater than or equal to the actual mean expression calculated. For all ligand-receptor pairs shown, we required a CellPhoneDB p-value of less than 10^-6^. Because many ligands and receptors are expressed by both neurons and astrocytes, we ran CellPhoneDB on only the neuronal and astrocytic clusters to increase specificity by removing cell types that could deflate the specificity p-value. To generate the dotplots shown in Fig. 4A-B and Fig. S12A-B, we restricted the plot to only those ligand-receptor (L-R) pairs that were near-unique (p-value below 10^−6^) to 2 or fewer (except for mouse P90, which was relaxed to 3 or fewer) neuronal clusters and the dominant astrocyte subtype (or vice-versa). Otherwise, the top 25 L-R pairs shown are mostly shared across all neuronal subtypes.

For the upset plots shown in Fig. 4C-D and Fig. S12C-D, non-filtered (non near-unique) L-R pairs were used. Because immature versions of the most abundant neuronal and astrocytic cluster dominated at early developmental time points in both species, L-R pairs between these immature clusters were used at these time points only, and only if their abundance was greater than the corresponding mature cluster. For example, in neonate mouse, we used L-R pairs between the ‘Neuron_Excit_Ctx_Immature_L23IT_Ptprk’ Leiden cluster and the ‘Astrocyte_Immature_Ptprz1’ Leiden cluster, while in adult mouse, we used L-R pairs between ‘Neuron_Excit_Ctx_L23IT_Cam2ka’ and ‘Astrocyte_Telen_Mature_Slc1a2’. Details are available in the Jupyter notebooks in our GitHub repository (https://github.com/Feng-Lab-MIT/AstrocyteHeterogeneity).

### Pseudotime analysis.

We performed pseudotime analysis using Palantir^59^, a scanpy-friendly package that orders cells along pseudo-temporal trajectories based on diffusion space and assigns each cell a probability of differentiating into each user-defined terminal state based on a Markov chain. Importantly, unlike single-cell velocity based methods that rely on estimates of spliced and unspliced counts^114–116^, Palantir runs diffusion maps in a latent space calculated from the regular counts matrix, which is in our case the scVI latent space (pca_key= “X_scVI”, n_components=5). The “determine_multiscale_space” parameter n_eigs was set to 5 to avoid a documented error (https://github.com/dpeerlab/Palantir/issues/84). Palantir imputes missing gene expression data in log1p-transformed counts per million space using MAGIC^117^, which is useful for visualizing gene expression. The root and terminal cells were manually specified for each cell type (oligodendrocyte lineage or astrocyte) based on their location in UMAP space combined with cluster and age information, and 500 waypoints were used. We calculated pseudotime trajectories for the astrocyte and oligodendrocyte lineages on a per-species region-combined basis. To reduce computational load, marmoset oligodendrocytes were randomly downsampled to 100,000 nuclei. We first ran Palantir pseudotime analysis on the oligodendrocyte lineage as a “sense check” for the algorithm, as the biological ground truth of oligodendrocyte lineage differentiation is well known (OPC→COP→NFOL→MFOL→MOL^60^), and found that Palantir’s calculated pseudotime was able to recapitulate the known differentiation trajectory.

### RNA fluorescence in situ hybridization (FISH).

Neonate (P4) or adult mice were deeply anesthetized via hypothermia (neonates) or isoflurane overdose (adults) and rapidly decapitated. Brains were extracted and frozen in OCT compound (Tissue-Tek) over dry ice and stored at −70°C. Briefly, neonate and adult marmosets were deeply sedated by intramuscular injection of ketamine (20–40 mg/kg) or alfaxalone (5–10 mg/kg), followed by intravenous injection of sodium pentobarbital (10–30 mg/kg). When the pedal with-drawal reflex was eliminated and/or the respiratory rate was diminished, animals were trans-cardially perfused with ice-cold sterile PBS. Whole brains were rapidly extracted into fresh PBS on ice for transfer to the lab (<30mins), then frozen in OCT over dry ice and stored at −70°C. Brains were sectioned sagittally on a cryostat (Leica) at −16um with a cutting temperature between −15 and −17°C and mounted on SuperFrost Plus (VWR/EMS) slides and stored at −70°C.

Multiplexed smFISH was performed using the RNA Scope HiPlex kit and protocol (Advanced Cell Diagnostics). Briefly, sections were removed from 70°C, placed directly in 4% PFA, and fixed for 1 hour at room temp. Next, sections were dehydrated via an ethanol series of 50%, 70%, 100%, and 100% EtOH in water, each immersion for 5 minutes at room temp. Samples were then treated with protease (Protease Plus for neonate mouse, Protease III for neonate marmoset, adult marmoset, and adult mouse). Probes (at a 1:50 dilution factor) were hybridized for 2 hours at 40°C. Following the application of amplifiers and fluorophores at 40°C, adult marmoset sections were incubated with TrueBlack Plus (Biotium) at a 1:30 dilution factor (1.5x). Sections were counterstained with DAPI and coverslips were affixed with ProLong Diamond Antifade Mountant (Thermo Fisher). Following each round of imaging, coverslips were removed by soaking slides in 4x SSC, fluorophores were cleaved, and the next set of tails were applied, followed by additional TrueBlack (for adult marmoset sections) and DAPI application (every other round for mouse, every round for marmoset) prior to mounting and acquiring images for the next round. The tail application and imaging order was as follows: T1–3, T4–6, T7–9, and T10–12 for neonate mouse, adult mouse, and neonate marmoset; T7–9, T4–6, T10–12, and T1–3 for adult marmoset. We changed the round order for adult marmoset after observing low signal-to-noise for T7–9 when imaged in the third round. Imaging control probes (housekeeping genes *POLR2A*, *PPIB*, and *UBC*) also showed significant loss of signal in the third round of imaging in adult marmoset brian.

Images were acquired using an Olympus Fluoview FV3000 confocal microscope using the multi-area time-lapse (MATL) module to record stage positions for re-use in subsequent imaging rounds. Imaging settings were adjusted on a per-experiment (and in rare cases, per-sample) basis due to batch-level variations in signal intensity so as to maximize the dynamic range of pixel intensities. All brain regions within a slide were imaged with the same laser power and voltage settings. Fields of view consisting 3×3 grids or smaller (for smaller regions in neonate tissue) with the 20x magnification objective lens were obtained in each brain region of interest (PFC, striatum, and thalamus). We obtained a z-stack covering the entire axial extent of the tissue section with a z-step size of 2um. Raw images from the microscope were converted to .tif format, flattened using a maximum intensity projection, and separated into individual channels. Round 2–4+ images were registered to round 1 using the DAPI channel and cropped to mutually overlapping area using the HiPlex Image Registration Software v2.1 (ACD, https://acdbio.com/rnascope%E2%84%A2-hiplex-image-registration-software-v21), or, in cases where v2.1 failed to register most of the field of view, HiPlex Image Registration software v1.0.0 (ACD, provided by their technical support team).

RNA quality was assessed using positive control probes targeting housekeeping genes for each species. These included *POLR2A*, *PPIB*, and *UBC* for marmoset and *Polr2a*, *PPIB*, *Ubc*, *Hprt*, *Actb*, *Tubb3*, *Bin1*, *Ldha*, *Gapdh*, *Pgk1*, *Bhlhe22*, and *Cplx2* for mouse (RNAscope HiPlex12 Positive Control Probe - Mm). The control experiments were always performed simultaneously with the experiments for rDEG probes of interest, using the same reagents and brain slices of minimal stereotaxic distance to the slice used for rDEG probes. Control probe signal was not quantified, but the brightness and density of probes that are expected to be expressed ubiquitously in most/all cells were manually observed to judge RNA quality for the inclusion or exclusion of experiments.

### FISH quantification with CellProfiler.

Quantification of RNAscope HiPlex FISH images was preregistered during or after data collection on the Open Science Framework (https://osf.io/crs7v/; https://doi.org/10.17605/OSF.IO/6KUB2) and conducted with the analyzer blinded to rDEG identity, for mouse. The following text has been adapted from the preregistration, with major deviations from the preregistration being noted here. CellProfiler 4.2.5 was used to quantify RNA signal in each nuclei of different regions. Variations between experiments such as tissue quality or probe freshness caused variations in fluorescence, so it was necessary to use separate CellProfiler pipelines for each experiment for mouse (with the exception of one adult mouse experiment that needed to be split by replicate) and neonate marmoset to account for those differences. The adult marmoset tissue had more dramatic difference in quality between replicates, requiring a separate pipeline for each replicate. Crucially, the CellProfiler pipelines were always identical within each slice of tissue, allowing for each replicate to have unbiased comparison between regions.

We first created binary masks to eliminate large artifacts (e.g., very large/bright debris), large tears in the tissue, areas of very high autofluorescence, out-of-focus areas, poorly registered areas, and/or nearby brain regions that were not the region of interest. In most cases, the cropped region of interest was greater than ~80% of the original. For adult marmoset tissue, which exhibited persistent lipofuscin autofluorescence in later rounds despite quenching with TrueBlack Plus, we automatically generated masks to eliminate this punctate fluorescence as follows: minimum intensity projection of all three channels in the third round, gaussian filter with sigma = 2, intensity thresholding at 1.5 standard deviations above the mean, and median filtering with radius of 2 pixels. We then combined this autofluorescence mask with the large artifact mask mentioned above. This quality control step was not mentioned in our preregistration, largely because we did not anticipate the extent of remaining autofluorescence. The registered, cropped, masked max-projected images were processed through the CellProfiler quantification pipeline.

In CellProfiler, all images were scaled to stretch to the full intensity range and masked to regions of interest. The lipofuscin mask was used to filter nuclei covered more than 20% by lipofuscin and crop any smaller areas within remaining nuclei. Some regions had a higher background intensity than others, so the images were binarized to eliminate background noise. Binarization was achieved using the CellProfiler “Threshold” module, with the threshold set manually after using the Otsu algorithm to approximate a value that would separate foreground and background. The manual threshold was used instead of Otsu, so that the definition of foreground and background would be consistent between regions. The exception to this was *Slc1a3*, our astrocyte marker, because its signal was known to be different between regions. Using this binarized signal, we were able to quantify the fluorescence from RNA in each nucleus. There were two rDEG probes in the adult mouse that required additional processing steps prior to binarization: *Sparc* and *Clmn* were particularly noisy in the thalamus specifically, with punctate signal outside of nuclei. Those genes required the “EnhanceOrSuppressFeatures” to suppress speckles. We also applied the “Smooth” module to *Sparc* in all adult mouse regions to reduce noise. From the binarized signal, we calculated mean intensity, or the fraction of the nucleus that is covered by the probe signal, and integrated intensity, the number of thresholded pixels within the area of the nucleus.

All nucleus objects identified by DAPI signal were expanded by 3 pixels to reflect RNA signal likely also existing outside of the nucleus, with the exception of P4 mouse nuclei, as they were more tightly packed together. To identify astrocytes, we first filtered by *SLC1A3/Slc1a3* mean intensity, using 6.5% for mouse astrocytes, 12.5% for neonate marmoset astrocytes, and 8% for adult marmoset astrocytes. This value was determined to reflect expected expression levels and similar fraction of astrocytes out of total cells based on the snRNAseq data. Next, we filtered out the astrocytes that had at least 50% mean intensity for neuronal markers *GAD2/Gad2*, *SLC17A6/Slc17a6*, and *SLC17A7/Slc17a7*. We did not filter based on *OLIG2/Olig2* because a subset of astrocytes are known to express *OLIG2*/*Olig2*^118^. We then measured the intensity of the binarized signal in our rDEG probes inside astrocytes and in all nuclei. To count a cell positive for a probe, the cell needed to either have at least 0.03 mean intensity or 6 integrated intensity. Both measurements were used to account for variations in cell size, as smaller cells would likely have lower integrated intensity and larger cells would likely have lower mean intensity. To compare rDEG expression between regions, we used the fraction of cells counted positive for a probe and the average mean intensity of all cells within the region. For mouse, these values were averaged within slices of the same replicate and then treated as a single data point.

We used DAPI signal, positive control probes, and cell type marker probe signals to qualitatively assess RNA quality (e.g., degraded or not degraded based on the brightness of fluorescence intensity) before running images through the analysis pipeline. If we observed unacceptably low tissue (e.g. over-digested, damaged, or folded slices or extremely high background) or RNA quality (little to no signal) for a given sample, we collected more images from a different slice or animal to maintain sample size prior to starting analysis. Given the low number of samples per region/age/species, we did not remove outliers, as we were not adequately powered to detect outliers post-hoc. Of note, changes were made to marmoset analysis pipelines after unblinding due to unrealistic detection of *FOXG1* expression in the thalamus. This gene is known to be a telencephalic patterning factor^119^, a finding confirmed *in situ* by other groups (see for example the RIKEN Marmoset Gene Atlas^120,121^ at https://gene-atlas.brainminds.jp/gene-image/?gene=370-6), triggering reevaluation of the lipofuscin masks and *FOXG1*’s binarization threshold. Ultimately, we excluded *FOXG1* from our quantification results because no version of our pipeline could overcome the large variations in signal-to-noise between brain regions.

Because we did not have sufficient sample size to test for statistical significance in marmoset, we report only observations and trends. For mouse data, we used 2 univariate ANOVAs with Benjamini–Hochberg p-value correction for each measure presented: fraction of astrocytes positive for a probe and mean fraction of the astrocyte covered by the probe (i.e., mean intensity). If the effect of brain region was significant overall, Tukey’s multiple comparisons (i.e., the Tukey HSD method) test was performed separately on each variable to test for the significance of pairwise differences between brain regions. Statistical analyses were performed in GraphPad Prism. Contrary to our preregistration, we did not first use a one-way repeated measures *multivariate* ANOVA, as we could not find a package to do so, but we believe this divergence to be minor given that we only report two outcome variables.

### Mouse astrocyte labeling and ExR for mouse tissue.

Aldh1l1-Cre (Jax #023748, colony founder was a gift from Dr. Jennifer Shih and Dr. Mriganka Sur) breeding pairs were housed in a facility with a light cycle running from 21:00 to 9:00, temperature 20–22.2°C, humidity 30–70%, and food and water available *ad libitum*. Pups were injected at postnatal day 1 (P1) or P2. Cre genotype was determined using PCR for Cre on genomic DNA extracted from tail samples with the following primers (5’−3’: GGTTCGCAAGAACCTGATGG (forward) and GCCTTCTCTACACCTGCGG (reverse)). Temporal vein injections were performed as previously described^122^, using 2 × 10^11^ viral genomes of PHP.eB-CAG-FLEX-GFP (Addgene #51502^69^) per animal. All AAVs in this study were packaged in-house as previously described^123^. Briefly, for each 150mm culture dish of HEK293 cells, 5.7μg of construct DNA was transfected with 22.8μg of capsid plasmid and 11.45μg of pADDeltaF6 using polyethylenimine 25K MW (Polysciences, 23966–1). Collection of cells and media for AAV harvesting began 72 hours after transfection, followed by iodixanol gradient ultracentrifugation purification using a Type 70 Ti Fixed-Angle Titanium Rotor (Beckman-Coulter, 337922). Titer was calculated using droplet digital PCR (ddPCR) as described by Addgene (https://www.addgene.org/protocols/aav-ddpcr-titration/)^124^ using the QX200 AutoDG Droplet Digital PCR System (BioRad, 1864100).

At 5–6 weeks of age, animals were perfused for ExR as described^34^. Briefly, animals were deeply anesthetized using isoflurane and transcardially perfused with ice-cold 2% acrylamide in PBS followed by ice-cold 30% acrylamide and 4% paraformaldehyde in PBS (by initial volume: e.g., for two mice, we dissolved 15g of acrylamide in 38.75mL deionized water, added 5mL 10x PBS, and 6.25mL 32% PFA). Brains were post-fixed in the same fixative solution overnight, transferred to 100mM glycine for 6 hours, and stored in PBS at 4°C until sectioning. Brains were sectioned coronally at 150μm on a vibrating microtome (Leica) and stained for GFP (primary antibody, Abcam chicken-anti-GFP at 1:1000, secondary antibody, AcX-conjugated goat-anti-chicken Alexa Fluor 488 at 1:200, see Table S27 for antibody product information). Briefly, sections were permeabilized in 1x PBS + 0.05 Triton X-100 solution for 10 minutes at RT, blocked for 2 hours at RT in blocking buffer (5% normal goat serum (NGS) + 0.5% Triton X-100 in 1x PBS), incubated with primary antibodies in carrier solution (5% NGS + 0.25% Triton X-100 in 1xPBS) for 12–24 hours at 4°C, washed in PBST (1x PBS + 0.1% Triton X-100) 3 times for 10 min each at RT, incubated with secondary antibodies in carrier solution for 12–24 hours at 4°C, and washed in PBST 3 times for 10 min each at RT. Slices containing regions of interest (PFC, striatum, and thalamus) were identified using the online Allen Brain Institute adult mouse reference atlas with coronal sections.

One hemisphere was expanded at ~4x expansion followed by staining for GFP, Lectin, and GFAP for morphology characterization, and either the other brain hemisphere (when available) or another hemisphere from a neighboring slice was expanded ~16x for multiplexed super-resolution imaging of astrocytic processes with synaptic proteins and rDEGs using the multiExR protocol (Kang et al., 2024, in press). ~4x expanded gels were generated using the ExR protocol^34^. Tissues were incubated in the first gelling solution for 30 min at 4°C followed by 37C for 30 min - 2 h. To preserve blood vessel morphology, gels were treated with 0.5 kU/mL Collagenase VII overnight at 37°C, a variation on our lab’s previous protocol^125^ (Fig. S18A). After collagenase treatment, tissue-embedded gels were incubated in ExR denaturation buffer for 1h at 95°C. Denatured gels were fully expanded in deionized water by washing 2–4 times for 15–45 min each. ~16x-expanded gels were generated and stained using the ExR/multiExR protocols without collagenase treatment^34^. We omitted collagenase from the multiExR samples for two reasons. First, the goal of this experiment was not to capture astrocyte morphology, which might be locally disrupted by blood vessel breakage (indeed, most of the fields of view we imaged contained no blood vessels). Nevertheless, despite some broken blood vessels, astrocyte morphology appeared largely continuous in non-collagenase treated samples, except specifically at astrocyte contact sites with blood vessels (Fig. S18B). Second, collagenases could in principle be contaminated with proteases, which might degrade sensitive epitopes and reduce signal captured by multiExR. To measure the expansion factor of multiExR gels, we used excess portions of the original, untrimmed gels, which were not used for imaging or staining, and measured an average expansion factor of 15.82x (Table S28).

After full expansion, we shrunk the gels for easier handling by incubating in 10x PBS for 10–30min. We then transferred gels to blocking buffer (5% NGS and 0.5% Triton X-100 in 1x PBS) and incubated for 90 mins - 2 hours at room temperature. Primary antibodies (chicken-anti-GFP and mouse-anti-GFAP, see Table S27 for antibody product information) were incubated at 4°C for 16–24 hours in antibody carrier solution (5% normal goat serum and 0.25% Triton X-100 in 1x PBS) at a dilution factor of 1:200. Gels were washed in 0.01% Triton X-100 in 1x PBS 6 times for 15 minutes each at room temperature, and then incubated with secondary antibodies (donkey-anti-goat AF488, donkey-anti-chicken AF488, donkey-anti-mouse AF555, and Lycopersicon Esculentum (Tomato) Lectin (LEL, TL), DyLight 649, see Table S27 for antibody product information) were incubated at 4°C for 16–24 hours in antibody carrier solution (5% normal goat serum and 0.25% Triton X-100 in 1x PBS) at a dilution factor of 1:200. Gels were washed in 0.05x PBST (e.g., 500uL Triton X-100 in 1xPBS, 25mL of 1x PBS, up to 500mL of DIW) 6 times for 15 minutes each at room temperature to expand the gels for imaging. For multiplexed ExR (multiExR), antibody stripping was conducted as previously described: incubation with ExR denaturation buffer + 100mM beta-mercaptoethanol for 45 min at 95°C, followed by 4×15 minute washes in 1x PBS, before proceeding with blocking and staining as described above (Kang et al., 2024, in press). Protein targets for each round of multiExR are summarized in Table S23.

The expansion factor for each gel was measured before and after expansion (in 0.05x PBST, the buffer used for washing before imaging) using a ruler. Measurement lengths smaller than 0.1cm, the smallest demarcation on the ruler, were estimated. We used the average expansion factor across samples for a given brain region or experiment to convert from physical (pre-expansion) to biological (post-expansion) units for scale bars, area, and volume calculations (Table S28). Final expansion factors for PFC, striatum, and thalamus were 4.22, 4.05, and 4.16, respectively. Images were obtained on an inverted Nikon w1 or SoRa confocal microscope with a 40x water magnification lens. Images for ~4x expanded single-round ExR gels were obtained at a 0.5μm z-step with 200ms exposure and 100% laser power for each optical channel on a Nikon SoRa confocal microscope. Since these stacks were very large (2048×2048×200+ voxel volumes), in order to span the full volume of astrocytes, we imaged the full z-stack for each channel separately using a Piezo Z-drive. When possible, we imaged the astrocytes that were: 1) brightly labeled with GFP, 2) fully contained within the gel (volume not cut off), 3) not overlapping with other brightly labeled astrocytes. During imaging, we observed some dimly labeled non-astrocyte structures that appeared to be neuronal somata or dendrites (resulting either from on-target Cre expression in *Aldh1l1*+ neurons or off-target GFP expression from CAG-FLEX-GFP^126,127^), and whenever possible, avoided imaging astrocytes with these structures in the field of view. After segmentation, we excluded 5 astrocytes that were either cut off in the z-axis or had their soma directly on blood vessels because they were morphologically very distinct (flatter, more elongated morphology, Fig. S17B). Notes on the quality of each imaged astrocyte region of interest are provided in Table S29. Images for ~16x expanded multiExR gels were obtained at a 0.25μm z-step with 1s exposure and 100% laser power for each optical channel on a Nikon w1 confocal microscope. We imaged 2048×2048×81 voxel volumes, with each z-step incrementing only after all 3 channels were imaged. multiExR gels were stained and imaged with the experimenter blinded to region identity.

### Marmoset astrocyte labeling and preparation of tissues for ExR.

Systemic AAV injection in marmoset was carried out as previously described^7^. Briefly, two wild-type adult female marmosets (one 4 years, 10 months, one 4 years, 5 months, see Table S1) were sedated with alfaxalone (5–10 mg/kg), catheterized in the tail vein, and injected with AAV-BI103-*gfaABC1D*-eGFP (cloned from pZac2.1 *gfaABC1D*-tdTomato^74^, Addgene #44332) at 6 (for 18–169) or 10 × 10^13^ vg/kg (for 19–241), followed by 1.5mL of saline to flush the line. The BI103 capsid, used in our prior study^7^ was a gift from Ben Deverman and Ken Chan. Marmoset 18–169 was injected with 6×10^13^ vg/mL of AAV BI103-*gfaABC1D*-eGFP, incubated for 43 days, and trans-cardially perfused with ice-cold PBS followed by 4% PFA. We observed brain-wide expression of GFP in astrocytes (Fig. S17E). To prepare marmoset tissue for expansion using the ExR protocol, we injected 19–241 with 1×10^14^ vg/kg AAV BI103-*gfaABC1D*-eGFP, waited 68 days before tissue harvest, and perfused with the ExR fixative (4% acrylamide and 30% paraformaldehyde). For perfusion, animals were deeply sedated by intramuscular injection of ketamine (20–40 mg/kg) or alfaxalone (5–10 mg/kg), followed by intravenous injection of sodium pentobarbital (10–30 mg/kg). When the pedal with-drawal reflex was eliminated and/or the respiratory rate was diminished, animals were trans-cardially perfused with ice-cold PBS followed by 4% paraformaldehyde (for 18–169) or ice-cold 30% acrylamide + 4% paraformaldehyde solution (for 19–241). The brain was extracted and post-fixed for one week in 4% PFA (for 18–169) or 24 hours in 30% acrylamide + 4% paraformaldehyde solution and transferred to 100mM glycine for 6 hours (for 19–241), and transferred to 1x PBS for storage. The brain was hemisectioned with a razor blade on a flat surface, sectioned sagittally on a vibratome at 80 (for 18–168) or 150μm (for 19–241), and prepared for expansion microscopy as described above (for 19–241). We used the Hardman and Ashwell marmoset brain atlas^128^ to select sagittal slices with our regions of interest.

### Immunostaining and ExR of marmoset tissue.

Conventional immunostaining for cell type markers was conducted on unexpanded marmoset slices as follows. 150μm sections were permeabilized in 1.2% Triton X-100 in PBS for 1 hour at room temperature (RT) and blocked in 3% normal goat serum and 0.1% Triton X-100 in 1x PBS for one hour at RT. Primary antibodies (chicken-anti-GFP at 1:1000, mouse-anti-GFAP at 1:1000, and Rabbit-anti-Olig2 at 1:500 or Rabbit-anti-NeuN at 1:250 in blocking buffer, see Table S27 for antibody product information) were incubated 12–48 hours at 4°C. Sections were then washed 6 times in 0.1% Triton X-100 in 1x PBS for 15 minutes each at RT. Primary antibodies (goat-anti-chicken AF488 at 1:1000 or 1:2000, goat-anti-mouse AF546 at 1:1000, and goat-anti-rabbit AF647 at 1:1000 in blocking buffer) were incubated 12–48 hours at 4°C. Sections were then washed 6 times in 0.1% Triton X-100 in 1x PBS for 15 minutes each at RT, with 1x DAPI included in the second-to-last wash. Lipofuscin autofluorescence was quenched by incubating the slices in 2x TrueBlack Plus (Biotium, diluted from stock at 1:20 in 1x PBS) for 10 minutes, followed by 3 quick rinses in 1x PBS. Sections were mounted on SuperFrost slides (VWR), partially dried, and coverslipped with Fluoromount G mounting medium. Slides were imaged on a TissueFAXS slide scanning confocal microscope (TissueGnostics) at 20x magnification with 405, 488, 594, and 647 nm lasers, or on an Olympus BX61 epifluorescence microscope with the GFP light emitting diode.

Marmoset tissue slices for expansion were pre-stained for GFP as described above for mouse. ~3x expanded gels were generated using the ExR protocol with collagenase treatment and stained as described above for mouse ~4x expanded gels. The expansion factor for each intact gel was measured before and after expansion (in 0.05x PBST, the buffer used for washing before imaging) using a ruler, and the mean across samples was used to calculate the final expansion factor of 3.10 (Table S28). As before, measurement lengths smaller than 0.1cm, the smallest demarcation on the ruler, were estimated. Images were obtained on an inverted Nikon w1 confocal microscope with a 40x water magnification lens at a 0.5μm z-step with 200ms exposure and 100% laser power for each optical channel. We imaged the full z-stack for each channel separately prior to proceeding to the next channel using the Nikon Ti Z-Drive to increase acquisition speed. Image volumes were background-subtracted in Fiji using the rolling ball algorithm with radius of 50. Supplementary Movies 1–18 were generated using Fiji’s Image→Stacks→3D project module with default settings, and exported to .avi with a frame rate of 7fps in jpeg format. Contrast was adjusted before 3D projection by navigating to the approximate middle of the stack and selecting “Reset” on the Fiji Brightness & Contrast window.

### Analysis of ExR and multiExR images.

Parameters for the following analyses were determined in advance of results compilation and statistical testing. All ExR and multiExR images were background-subtracted in Fiji using the rolling ball algorithm, radius of 50 pixels, as in our previous studies^34^ (Kang et al., 2024, in press). To quantify astrocyte morphology from ~4x expanded astrocytes, images were processed as follows after background subtraction: conversion to grayscale, gaussian filtering with sigma = 2 using MATLAB’s “imgaussfilt3”, binarization with an intensity threshold at 1 standard deviation above the mean across the whole stack, and size filtering with a minimum of 10^7^ voxels using MATLAB’s “bwareaopen”. If there were two connected components (MATLAB’s “bwconncomp”, connectivity of 26) over the minimum size filter, a new size filter was set at 10 voxels less than the volume of the largest connected component, so that only the largest astrocyte remained. Volume and surface area were then calculated from the largest connected component using MATLAB’s “regionprops3”, and converted to cubic and square microns using a weighted average of the physical pixel size divided by the expansion factor (e.g., ((⅔)*(0.1625/expansion_factor) + (⅓)*(0.5/expansion_factor))^3^ for volume).

Sholl analysis was run using Fiji’s SNT^129^ Sholl Analysis^71^ plugin, installed and accessed via the Neuroanatomy plugin, as described: https://imagej.net/plugins/snt/#installation. The center of each soma was manually annotated using the point selection tool and saved as an overlay. Sholl analysis was run in batch mode for each image using a custom macro that calls the legacy version of SNT’s Sholl Analysis, with the following parameters: start radius of 5μm, end radius of 50μm, step size 2.5μm, and no polynomial fitting. The number of intersections at each radius for each cell was averaged over all non-excluded astrocytes in each region to create the plots in Fig. 7E(iv).

Analysis of fractal dimension was performed in MATLAB on 3D binary segmentations using the box counting method via “boxcount”, available on the MATLAB file exchange at https://www.mathworks.com/matlabcentral/fileexchange/13063-boxcount (written by Frederick Moisy, accessed August 2024). Briefly, a fractal set (in this case, a binary image of an astrocyte) is one that exhibits self-similarity at progressively smaller scales. The box counting method can be used to characterize the extent to which a set is fractal by counting the number of boxes of size R in a grid needed to cover the edges (in our case, transition from black to white) of an image at progressively smaller grid sizes^70^. A fractal image will have exponentially more detail (edges) at smaller grid sizes, require exponentially more boxes of size R to cover the set, and therefore will have a negatively sloped line on the log-log plot of N, the number of boxes vs. R, the size of each box in the grid. For a straightforward explanation of box counting, see https://fractalfoundation.org/OFC/OFC-10-5.html. The local fractal dimension can therefore be calculated as the slope of the log(N) vs. log(R) plot (df = - diff(log(n))./diff(log(r)), as in the boxcount function “Examples” tab). We took the mean fractal dimension over all 12 local slopes on the line to arrive at a single measure of fractal dimension for each astrocyte.

Statistical significance of differences between astrocytes from different brain regions was determined using a linear mixed effects model via the scipy statsmodels package’s “mixedlm” function (https://www.statsmodels.org/stable/mixed_linear.html), where the outcome variable was the measure of interest, the coefficient was region assignment, and “animal” was the random effect group variable. P-values on the coefficients from striatum and thalamus were corrected for multiple comparisons with the Benjamini-Hochberg method using the scipy stats package’s “false_discovery_control” function (https://docs.scipy.org/doc/scipy/reference/generated/scipy.stats.false_discovery_control.html).

multiExR image stacks from different images rounds were registered as previously described (Kang et al., 2024, in press). Briefly, we registered round 1.1 and round 2 images to round 1.2 with the normalized sum of all channels as reference using the ExSeqProcessing registration pipeline^130^, available at https://github.com/dgoodwin208/ExSeqProcessing. 7 out of 30 imaged fields of view had poor quality or failed registrations in one of the two rounds. Maximum intensity projections of the GFP and target protein channels were examined to choose well-registered, high-quality fields of view for display in Fig. 7F. We excluded Kv4.2 from Fig. 7 due to low signal-to-noise and insufficient stripping of the pre-stained GFP in the same channel.

### Generative AI.

ChatGPT (OpenAI, GPT-3.5 or 4.0) was used to aid computational scripting and debugging. On these occasions, we prompted it to interpret error messages and some lines of code generated by others, generate bash commands and scripts, accelerate existing Python code, and generate some Python functions and code chunks for niche tasks (e.g., generating Venn diagrams and raster plots). ChatGPT-generated code blocks are annotated as such in our Python notebooks. However, ChatGPT did not meaningfully contribute to the intellectual development of experimental design, data analysis, data interpretation, or writing of this manuscript.

## Supplementary Material

Supplement 1

Supplement 2

## References

[1] Yao Z. A high-resolution transcriptomic and spatial atlas of cell types in the whole mouse brain. Nature 624, 317–332 (2023). 38092916 10.1038/s41586-023-06812-z PMC10719114

[2] Langlieb J. The molecular cytoarchitecture of the adult mouse brain. Nature 624, 333–342 (2023). 38092915 10.1038/s41586-023-06818-7 PMC10719111

[3] Aldridge S. & Teichmann S. A. Single cell transcriptomics comes of age. Nat. Commun. 11, 4307 (2020). 32855414 10.1038/s41467-020-18158-5 PMC7453005

[4] Siletti K. Transcriptomic diversity of cell types across the adult human brain. Science 382, eadd7046 (2023). 10.1126/science.add7046 37824663

[5] La Manno G. Molecular architecture of the developing mouse brain. Nature 596, 92–96 (2021). 34321664 10.1038/s41586-021-03775-x

[6] Braun E. Comprehensive cell atlas of the first-trimester developing human brain. Science 382, eadf1226 (2023). 10.1126/science.adf1226 37824650

[7] Krienen F. M. A marmoset brain cell census reveals regional specialization of cellular identities. Sci. Adv. 9, eadk3986 (2023). 10.1126/sciadv.adk3986 PMC10569717 37824615

[8] Lin J.-P. Transcriptomic architecture of nuclei in the marmoset CNS. Nat. Commun. 13, 5531 (2022). 36130924 10.1038/s41467-022-33140-z PMC9492672

[9] Yao Z. A taxonomy of transcriptomic cell types across the isocortex and hippocampal formation. Cell 184, 3222–3241.e26 (2021). 34004146 10.1016/j.cell.2021.04.021 PMC8195859

[10] Allen N. J. & Barres B. A. Glia — more than just brain glue. Nature 457, 675–677 (2009). 19194443 10.1038/457675a

[11] Ben Haim L. & Rowitch D. H. Functional diversity of astrocytes in neural circuit regulation. Nat. Rev. Neurosci. 18, 31–41 (2016). 27904142 10.1038/nrn.2016.159

[12] Oliveira J. F. & Araque A. Astrocyte regulation of neural circuit activity and network states. Glia 70, 1455–1466 (2022). 35460131 10.1002/glia.24178 PMC9232995

[13] Lee H. G., Wheeler M. A. & Quintana F. J. Function and therapeutic value of astrocytes in neurological diseases. Nat. Rev. Drug Discov. 2022 215 21, 339–358 (2022). 10.1038/s41573-022-00390-x PMC9081171 35173313

[14] Verkhratsky A. & Nedergaard M. Physiology of Astroglia. Physiol. Rev. 98, 239–389 (2018). 29351512 10.1152/physrev.00042.2016 PMC6050349

[15] Ramón Y Cajal S., Swanson N. & Swanson L. Histology of the Nervous System of Man and Vertebrates. (Oxford University Press, Oxford, New York, 1995).

[16] Oberheim N. A., Goldman S. A. & Nedergaard M. Heterogeneity of astrocytic form and function. Methods Mol. Biol. 814, 23–45 (2012). 22144298 10.1007/978-1-61779-452-0_3 PMC3506190

[17] Saunders A. Molecular Diversity and Specializations among the Cells of the Adult Mouse Brain. Cell 174, 1015–1030.e16 (2018). 30096299 10.1016/j.cell.2018.07.028 PMC6447408

[18] Batiuk M. Y. Identification of region-specific astrocyte subtypes at single cell resolution. Nat. Commun. 11, 1220 (2020). 32139688 10.1038/s41467-019-14198-8 PMC7058027

[19] Bayraktar O. A. Astrocyte layers in the mammalian cerebral cortex revealed by a single-cell in situ transcriptomic map. Nat. Neurosci. 23, 500–509 (2020). 32203496 10.1038/s41593-020-0602-1 PMC7116562

[20] Zeisel A. Molecular Architecture of the Mouse Nervous System. Cell 174, 999–1014.e22 (2018). 30096314 10.1016/j.cell.2018.06.021 PMC6086934

[21] Morel L. Molecular and Functional Properties of Regional Astrocytes in the Adult Brain. J. Neurosci. 37, 8706–8717 (2017). 28821665 10.1523/JNEUROSCI.3956-16.2017 PMC5588463

[22] Bachoo R. M. Molecular diversity of astrocytes with implications for neurological disorders. Proc. Natl. Acad. Sci. 101, 8384–8389 (2004). 15155908 10.1073/pnas.0402140101 PMC420403

[23] Hochstim C., Deneen B., Lukaszewicz A., Zhou Q. & Anderson D. J. Identification of Positionally Distinct Astrocyte Subtypes whose Identities Are Specified by a Homeodomain Code. Cell 133, 510–522 (2008). 18455991 10.1016/j.cell.2008.02.046 PMC2394859

[24] Tsai H. H. Regional astrocyte allocation regulates CNS synaptogenesis and repair. Science 337, 358–362 (2012). 22745251 10.1126/science.1222381 PMC4059181

[25] Welle A. Epigenetic control of region-specific transcriptional programs in mouse cerebellar and cortical astrocytes. Glia 69, 2160–2177 (2021). 34028094 10.1002/glia.24016

[26] Clavreul S. Cortical astrocytes develop in a plastic manner at both clonal and cellular levels. Nat. Commun. 10, (2019). 10.1038/s41467-019-12791-5 PMC6814723 31653848

[27] Farmer W. T. Neurons diversify astrocytes in the adult brain through sonic hedgehog signaling. Science 351, 849–854 (2016). 26912893 10.1126/science.aab3103

[28] Xie Y. Developmental origin and local signals cooperate to determine septal astrocyte identity. 2023.10.08.561428 Preprint at 10.1101/2023.10.08.561428 (2023).

[29] Molofsky A. V. & Deneen B. Astrocyte development: A Guide for the Perplexed. Glia 63, 1320–1329 (2015). 25963996 10.1002/glia.22836

[30] Tung P. Y. Batch effects and the effective design of single-cell gene expression studies. Sci. Rep. 2017 71 7, 1–15 (2017). 10.1038/srep39921 PMC5206706 28045081

[31] Tran H. T. N. A benchmark of batch-effect correction methods for single-cell RNA sequencing data. Genome Biol. 21, 1–32 (2020). 10.1186/s13059-019-1850-9 PMC6964114 31948481

[32] Zhou B., Zuo Y.-X. & Jiang R.-T. Astrocyte morphology: Diversity, plasticity, and role in neurological diseases. CNS Neurosci. Ther. 25, 665–673 (2019). 30929313 10.1111/cns.13123 PMC6515705

[33] Baldwin K. T., Murai K. K. & Khakh B. S. Astrocyte morphology. Trends Cell Biol. 34, 547–565 (2024). 38180380 10.1016/j.tcb.2023.09.006 PMC11590062

[34] Sarkar D. Revealing nanostructures in brain tissue via protein decrowding by iterative expansion microscopy. Nat. Biomed. Eng. 6, 1057–1073 (2022). 36038771 10.1038/s41551-022-00912-3 PMC9551354

[35] Traag V., Waltman L. & van Eck N. J. From Louvain to Leiden: guaranteeing well-connected communities. Sci. Rep. 9, (2018). 10.1038/s41598-019-41695-z PMC6435756 30914743

[36] Allen Institute for Brain Science. MapMyCells.

[37] Büttner M., Ostner J., Müller C. L., Theis F. J. & Schubert B. scCODA is a Bayesian model for compositional single-cell data analysis. Nat. Commun. 12, 6876 (2021). 34824236 10.1038/s41467-021-27150-6 PMC8616929

[38] Rubenstein J. L. R., Shimamura K., Martinez S. & Puelles L. REGIONALIZATION OF THE PROSENCEPHALIC NEURAL PLATE. Annu. Rev. Neurosci. 21, 445–477 (1998). 9530503 10.1146/annurev.neuro.21.1.445

[39] Sasai Y. & De Robertis E. M. Ectodermal Patterning in Vertebrate Embryos. Dev. Biol. 182, 5–20 (1997). 9073437 10.1006/dbio.1996.8445

[40] Di Bella D. J. Molecular logic of cellular diversification in the mouse cerebral cortex. Nature 595, 554–559 (2021). 34163074 10.1038/s41586-021-03670-5 PMC9006333

[41] Anthony T. E., Klein C., Fishell G. & Heintz N. Radial Glia Serve as Neuronal Progenitors in All Regions of the Central Nervous System. Neuron 41, 881–890 (2004). 15046721 10.1016/s0896-6273(04)00140-0

[42] Rowitch D. H. & Kriegstein A. R. Developmental genetics of vertebrate glial–cell specification. Nature 468, 214–222 (2010). 21068830 10.1038/nature09611

[43] Frassoni C., Amadeo A., Ortino B., Jaranowska A. & Spreafico R. Organization of radial and non-radial glia in the developing rat thalamus. J. Comp. Neurol. 428, 527–542 (2000). 11074449 10.1002/1096-9861(20001218)428:3<527::aid-cne9>3.0.co;2-x

[44] Tan C. X. & Eroglu C. Cell adhesion molecules regulating astrocyte–neuron interactions. Curr. Opin. Neurobiol. 69, 170–177 (2021). 33957433 10.1016/j.conb.2021.03.015 PMC8387342

[45] Sobolczyk M. & Boczek T. Astrocytic Calcium and cAMP in Neurodegenerative Diseases. Front. Cell. Neurosci. 16, (2022). 10.3389/fncel.2022.889939 PMC9161693 35663426

[46] Zhou Z. Astrocytic cAMP modulates memory via synaptic plasticity. Proc. Natl. Acad. Sci. U. S. A. 118, e2016584118 (2021). 33452135 10.1073/pnas.2016584118 PMC7826339

[47] Bazargani N. & Attwell D. Astrocyte calcium signaling: The third wave. Nat. Neurosci. 19, 182–189 (2016). 26814587 10.1038/nn.4201

[48] Kucukdereli H. Control of excitatory CNS synaptogenesis by astrocyte-secreted proteins hevin and SPARC. Proc. Natl. Acad. Sci. U. S. A. 108, (2011). 10.1073/pnas.1104977108 PMC3156217 21788491

[49] Elizarraras J. M. WebGestalt 2024: faster gene set analysis and new support for metabolomics and multi-omics. Nucleic Acids Res. 52, W415–W421 (2024). 38808672 10.1093/nar/gkae456 PMC11223849

[50] Zhang B., Kirov S. & Snoddy J. WebGestalt: an integrated system for exploring gene sets in various biological contexts. Nucleic Acids Res. 33, W741–W748 (2005). 15980575 10.1093/nar/gki475 PMC1160236

[51] Carpenter A. E. CellProfiler: image analysis software for identifying and quantifying cell phenotypes. Genome Biol. 7, R100 (2006). 17076895 10.1186/gb-2006-7-10-r100 PMC1794559

[52] Stirling D. R. CellProfiler 4: improvements in speed, utility and usability. BMC Bioinformatics 22, 433 (2021). 34507520 10.1186/s12859-021-04344-9 PMC8431850

[53] Hodge R. D. Conserved cell types with divergent features in human versus mouse cortex. Nat. 2019 5737772 573, 61–68 (2019). 10.1038/s41586-019-1506-7 PMC6919571 31435019

[54] Chai H. Neural Circuit-Specialized Astrocytes: Transcriptomic, Proteomic, Morphological, and Functional Evidence. Neuron 95, 531–549.e9 (2017). 28712653 10.1016/j.neuron.2017.06.029 PMC5811312

[55] Fang R. Conservation and divergence of cortical cell organization in human and mouse revealed by MERFISH. Science 377, 56–62 (2022). 35771910 10.1126/science.abm1741 PMC9262715

[56] Halassa M. M., Fellin T., Takano H., Dong J.-H. & Haydon P. G. Synaptic Islands Defined by the Territory of a Single Astrocyte. J. Neurosci. 27, 6473–6477 (2007). 17567808 10.1523/JNEUROSCI.1419-07.2007 PMC6672436

[57] Delaunay D. Early Neuronal and Glial Fate Restriction of Embryonic Neural Stem Cells. J. Neurosci. 28, 2551–2562 (2008). 18322099 10.1523/JNEUROSCI.5497-07.2008 PMC6671176

[58] Hong W. Temporal-spatial Generation of Astrocytes in the Developing Diencephalon. Neurosci. Bull. 40, 1–16 (2024). 37843774 10.1007/s12264-023-01131-9 PMC10774245

[59] Setty M. Characterization of cell fate probabilities in single-cell data with Palantir. Nat. Biotechnol. 37, 451–460 (2019). 30899105 10.1038/s41587-019-0068-4 PMC7549125

[60] Marques S. Oligodendrocyte heterogeneity in the mouse juvenile and adult central nervous system. Science 352, 1326–1329 (2016). 27284195 10.1126/science.aaf6463 PMC5221728

[61] Zhang Y. Purification and Characterization of Progenitor and Mature Human Astrocytes Reveals Transcriptional and Functional Differences with Mouse. Neuron 89, 37–53 (2016). 26687838 10.1016/j.neuron.2015.11.013 PMC4707064

[62] Han X. Forebrain Engraftment by Human Glial Progenitor Cells Enhances Synaptic Plasticity and Learning in Adult Mice. Cell Stem Cell 12, 342–353 (2013). 23472873 10.1016/j.stem.2012.12.015 PMC3700554

[63] Xu C. Probabilistic harmonization and annotation of single‐cell transcriptomics data with deep generative models. Mol. Syst. Biol. 17, e9620 (2021). 33491336 10.15252/msb.20209620 PMC7829634

[64] Rosen Y. Toward universal cell embeddings: integrating single-cell RNA-seq datasets across species with SATURN. Nat. Methods 1–9 (2024) doi:10.1038/s41592-024-02191-z. 38366243 PMC11310084

[65] Bakken T. E. Comparative cellular analysis of motor cortex in human, marmoset and mouse. Nat. 2021 5987879 598, 111–119 (2021). 10.1038/s41586-021-03465-8 PMC8494640 34616062

[66] Jorstad N. L. Comparative transcriptomics reveals human-specific cortical features. Science 382, eade9516 (2023). 10.1126/science.ade9516 PMC10659116 37824638

[67] Krienen F. M. Innovations present in the primate interneuron repertoire. Nature 586, 262–269 (2020). 32999462 10.1038/s41586-020-2781-z PMC7957574

[68] Endo F. Molecular basis of astrocyte diversity and morphology across the CNS in health and disease. Science 378, (2022). 10.1126/science.adc9020 PMC9873482 36378959

[69] Oh S. W. A mesoscale connectome of the mouse brain. Nature 508, 207–214 (2014). 24695228 10.1038/nature13186 PMC5102064

[70] Liebovitch L. S. & Toth T. A fast algorithm to determine fractal dimensions by box counting. Phys. Lett. A 141, 386–390 (1989).

[71] Ferreira T. A. Neuronal morphometry directly from bitmap images. Nat. Methods 11, 982–984 (2014). 25264773 10.1038/nmeth.3125 PMC5271921

[72] Soto J. S. Astrocyte Gi-GPCR signaling corrects compulsive-like grooming and anxiety-related behaviors in Sapap3 knockout mice. Neuron 0, (2024). 10.1016/j.neuron.2024.07.019 PMC11512628 39163865

[73] Baldwin K. T., Murai K. K. & Khakh B. S. Astrocyte morphology. Trends Cell Biol. 0, (2023). 10.1016/j.tcb.2023.09.006 PMC11590062 38180380

[74] Shigetomi E. Imaging calcium microdomains within entire astrocyte territories and endfeet with GCaMPs expressed using adeno-associated viruses. J. Gen. Physiol. 141, 633–647 (2013). 23589582 10.1085/jgp.201210949 PMC3639581

[75] Heffernan K. S., Rahman K., Smith Y. & Galvan A. Characterization of the GfaABC1D promoter to selectively target astrocytes in the rhesus macaque brain. J. Neurosci. Methods 372, 109530 (2022). 35202614 10.1016/j.jneumeth.2022.109530 PMC8940704

[76] Falcone C. Redefining varicose projection astrocytes in primates. Glia 70, 145–154 (2022). 34533866 10.1002/glia.24093

[77] Zhang Y. & Barres B. A. Astrocyte heterogeneity: an underappreciated topic in neurobiology. Curr. Opin. Neurobiol. 20, 588–594 (2010). 20655735 10.1016/j.conb.2010.06.005

[78] Bayraktar O. A., Fuentealba L. C., Alvarez-Buylla A. & Rowitch D. H. Astrocyte Development and Heterogeneity. Cold Spring Harb. Perspect. Biol. 7, a020362 (2015). 10.1101/cshperspect.a020362 PMC4292163 25414368

[79] Schober A. L., Wicki-Stordeur L. E., Murai K. K. & Swayne L. A. Foundations and implications of astrocyte heterogeneity during brain development and disease. Trends Neurosci. (2022) doi:10.1016/J.TINS.2022.06.009. 35879116

[80] Semple B. D., Blomgren K., Gimlin K., Ferriero D. M. & Noble-Haeusslein L. J. Brain development in rodents and humans: Identifying benchmarks of maturation and vulnerability to injury across species. Prog. Neurobiol. 106–107, 1–16 (2013). 10.1016/j.pneurobio.2013.04.001 PMC3737272 23583307

[81] Freeman M. R. Specification and Morphogenesis of Astrocytes. Science 330, 774–778 (2010). 21051628 10.1126/science.1190928 PMC5201129

[82] Cho F. S. Enhancing GAT-3 in thalamic astrocytes promotes resilience to brain injury in rodents. Sci. Transl. Med. 14, 4310 (2022). 10.1126/scitranslmed.abj4310 PMC9491689 35857628

[83] Craig A. M. & Kang Y. Neurexin–neuroligin signaling in synapse development. Curr. Opin. Neurobiol. 17, 43–52 (2007). 17275284 10.1016/j.conb.2007.01.011 PMC2820508

[84] Holt L. M. Astrocyte morphogenesis is dependent on BDNF signaling via astrocytic TrkB.T1. eLife 8, e44667 (2019). 31433295 10.7554/eLife.44667 PMC6726422

[85] Nowakowski T. J. Spatiotemporal gene expression trajectories reveal developmental hierarchies of the human cortex. Science 358, 1318–1323 (2017). 29217575 10.1126/science.aap8809 PMC5991609

[86] Breschi A., Gingeras T. R. & Guigó R. Comparative transcriptomics in human and mouse. Nat. Rev. Genet. 18, 425–440 (2017). 28479595 10.1038/nrg.2017.19 PMC6413734

[87] Okano H., Hikishima K., Iriki A. & Sasaki E. The common marmoset as a novel animal model system for biomedical and neuroscience research applications. Semin. Fetal. Neonatal Med. 17, 336–340 (2012). 22871417 10.1016/j.siny.2012.07.002

[88] Abbott D. H., Barnett D. K., Colman R. J., Yamamoto M. E. & Schultz-Darken N. J. Aspects of Common Marmoset Basic Biology and Life History Important for Biomedical Research. Comp. Med. 53, 339–350 (2003). 14524409

[89] Lindhout F. W., Krienen F. M., Pollard K. S. & Lancaster M. A. A molecular and cellular perspective on human brain evolution and tempo. Nature 630, 596–608 (2024). 38898293 10.1038/s41586-024-07521-x

[90] Khakh B. S. & Deneen B. The Emerging Nature of Astrocyte Diversity. Annu. Rev. Neurosci. 42, 187–207 (2019). 31283899 10.1146/annurev-neuro-070918-050443

[91] Cheng Y.-T. Inhibitory input directs astrocyte morphogenesis through glial GABABR. Nature 617, 369–376 (2023). 37100909 10.1038/s41586-023-06010-x PMC10733939

[92] Paxinos G., Watson C., Petrides M., Rosa M. & Tokuno H. The Marmoset Brain in Stereotaxic Coordinates. (Elsevier, 2012).

[93] Pool A.-H., Poldsam H., Chen S., Thomson M. & Oka Y. Recovery of missing single-cell RNA-sequencing data with optimized transcriptomic references. Nat. Methods 20, 1506–1515 (2023). 37697162 10.1038/s41592-023-02003-w

[94] Fleming S. J. Unsupervised removal of systematic background noise from droplet-based single-cell experiments using CellBender. Nat. Methods 20, 1323–1335 (2023). 37550580 10.1038/s41592-023-01943-7

[95] Virshup I. The scverse project provides a computational ecosystem for single-cell omics data analysis. Nat. Biotechnol. 41, 604–606 (2023). 37037904 10.1038/s41587-023-01733-8

[96] Takabayashi S. & Katoh H. Sex Identification Using the ZFX and ZFY Genes in Common Marmosets (Callithrix jacchus). Exp. Anim. 60, 417–420 (2011). 21791881 10.1538/expanim.60.417

[97] Zargari M. Fetal Sex Determination using Non-Invasive Method of Cell-free Fetal DNA in Maternal Plasma of Pregnant Women During 6th– 10th Weeks of Gestation. Avicenna J. Med. Biotechnol. 3, 201–206 (2011). 23407464 PMC3558193

[98] Wolf F. A., Angerer P. & Theis F. J. SCANPY: large-scale single-cell gene expression data analysis. Genome Biol. 19, 15 (2018). 29409532 10.1186/s13059-017-1382-0 PMC5802054

[99] Lopez R., Regier J., Cole M. B., Jordan M. I. & Yosef N. Deep generative modeling for single-cell transcriptomics. Nat. Methods 15, 1053–1058 (2018). 30504886 10.1038/s41592-018-0229-2 PMC6289068

[100] Becht E. Dimensionality reduction for visualizing single-cell data using UMAP. Nat. Biotechnol. 37, 38–44 (2019). 10.1038/nbt.4314 30531897

[101] Bernstein N. J. Solo: Doublet Identification in Single-Cell RNA-Seq via Semi-Supervised Deep Learning. Cell Syst. 11, 95–101.e5 (2020). 32592658 10.1016/j.cels.2020.05.010

[102] Heumos L. Best practices for single-cell analysis across modalities. Nat. Rev. Genet. 24, 550–572 (2023). 37002403 10.1038/s41576-023-00586-w PMC10066026

[103] Kolberg L. g:Profiler—interoperable web service for functional enrichment analysis and gene identifier mapping (2023 update). Nucleic Acids Res. 51, W207–W212 (2023). 37144459 10.1093/nar/gkad347 PMC10320099

[104] Cunningham F. Ensembl 2019. Nucleic Acids Res. 47, D745–D751 (2019). 30407521 10.1093/nar/gky1113 PMC6323964

[105] van Dijk D. Recovering Gene Interactions from Single-Cell Data Using Data Diffusion. Cell 174, 716–729.e27 (2018). 29961576 10.1016/j.cell.2018.05.061 PMC6771278

[106] Lex A., Gehlenborg N., Strobelt H., Vuillemot R. & Pfister H. UpSet: Visualization of Intersecting Sets. IEEE Trans. Vis. Comput. Graph. 20, 1983–1992 (2014). 26356912 10.1109/TVCG.2014.2346248 PMC4720993

[107] Ashburner M. Gene Ontology: tool for the unification of biology. Nat. Genet. 25, 25–29 (2000). 10802651 10.1038/75556 PMC3037419

[108] The Gene Ontology Consortium The Gene Ontology knowledgebase in 2023. Genetics 224, iyad031 (2023). 10.1093/genetics/iyad031 PMC10158837 36866529

[109] Kanehisa M. & Goto S. KEGG: Kyoto Encyclopedia of Genes and Genomes. Nucleic Acids Res. 28, 27–30 (2000). 10592173 10.1093/nar/28.1.27 PMC102409

[110] Lin Z. Evolutionary-scale prediction of atomic-level protein structure with a language model. Science 379, 1123–1130 (2023). 36927031 10.1126/science.ade2574

[111] Geirsdottir L. Cross-Species Single-Cell Analysis Reveals Divergence of the Primate Microglia Program. Cell 179, 1609–1622.e16 (2019). 31835035 10.1016/j.cell.2019.11.010

[112] Efremova M., Vento-Tormo M., Teichmann S. A. & Vento-Tormo R. CellPhoneDB: inferring cell–cell communication from combined expression of multi-subunit ligand–receptor complexes. Nat. Protoc. 15, 1484–1506 (2020). 32103204 10.1038/s41596-020-0292-x

[113] Dimitrov D. Comparison of methods and resources for cell-cell communication inference from single-cell RNA-Seq data. Nat. Commun. 13, 3224 (2022). 35680885 10.1038/s41467-022-30755-0 PMC9184522

[114] Bergen V., Lange M., Peidli S., Wolf F. A. & Theis F. J. Generalizing RNA velocity to transient cell states through dynamical modeling. Nat. Biotechnol. 38, 1408–1414 (2020). 32747759 10.1038/s41587-020-0591-3

[115] La Manno G. RNA velocity of single cells. Nature 560, 494–498 (2018). 30089906 10.1038/s41586-018-0414-6 PMC6130801

[116] Aivazidis A. Model-based inference of RNA velocity modules improves cell fate prediction. 2023.08.03.551650 Preprint at 10.1101/2023.08.03.551650 (2023).

[117] Dijk D. van MAGIC: A diffusion-based imputation method reveals gene-gene interactions in single-cell RNA-sequencing data. 111591 Preprint at 10.1101/111591 (2017).

[118] Wang H. Region-specific distribution of Olig2-expressing astrocytes in adult mouse brain and spinal cord. Mol. Brain 14, 36 (2021). 33618751 10.1186/s13041-021-00747-0 PMC7901088

[119] Martynoga B., Morrison H., Price D. J. & Mason J. O. Foxg1 is required for specification of ventral telencephalon and region-specific regulation of dorsal telencephalic precursor proliferation and apoptosis. Dev. Biol. 283, 113–127 (2005). 15893304 10.1016/j.ydbio.2005.04.005

[120] Kita Y. Cellular-resolution gene expression profiling in the neonatal marmoset brain reveals dynamic species- and region-specific differences. Proc. Natl. Acad. Sci. 118, e2020125118 (2021). 33903237 10.1073/pnas.2020125118 PMC8106353

[121] Shimogori T. Digital gene atlas of neonate common marmoset brain. Neurosci. Res. 128, 1–13 (2018). 29111135 10.1016/j.neures.2017.10.009

[122] Gombash Lampe S. E., Kaspar B. K. & Foust K. D. Intravenous Injections in Neonatal Mice. J. Vis. Exp. JoVE 52037 (2014) doi:10.3791/52037. PMC4353426 25407048

[123] Challis R. C. Systemic AAV vectors for widespread and targeted gene delivery in rodents. Nat. Protoc. 14, 379–414 (2019). 30626963 10.1038/s41596-018-0097-3 PMC13333184

[124] Lock M., Alvira M. R., Chen S.-J. & Wilson J. M. Absolute Determination of Single-Stranded and Self-Complementary Adeno-Associated Viral Vector Genome Titers by Droplet Digital PCR. Hum. Gene Ther. Methods 25, 115–125 (2014). 24328707 10.1089/hgtb.2013.131 PMC3991984

[125] Valdes P. A. Improved immunostaining of nanostructures and cells in human brain specimens through expansion-mediated protein decrowding. Sci. Transl. Med. 16, eabo0049 (2024). 10.1126/scitranslmed.abo0049 PMC10911838 38295184

[126] Botterill J. J. Off-Target Expression of Cre-Dependent Adeno-Associated Viruses in Wild-Type C57BL/6J Mice. eNeuro 8, (2021). 10.1523/ENEURO.0363-21.2021 PMC8614227 34785571

[127] Fischer K. B., Collins H. K. & Callaway E. M. Sources of off-target expression from recombinase-dependent AAV vectors and mitigation with cross-over insensitive ATG-out vectors. Proc. Natl. Acad. Sci. 116, 27001–27010 (2019). 31843925 10.1073/pnas.1915974116 PMC6936690

[128] Hardman C. D. & Ashwell K. W. S. Stereotaxic and Chemoarchitectural Atlas of the Brain of the Common Marmoset (Callithrix Jacchus). (CRC Press, 2012).

[129] Arshadi C., Günther U., Eddison M., Harrington K. I. S. & Ferreira T. A. SNT: a unifying toolbox for quantification of neuronal anatomy. Nat. Methods 18, 374–377 (2021). 33795878 10.1038/s41592-021-01105-7

[130] Alon S. Expansion sequencing: Spatially precise in situ transcriptomics in intact biological systems. Science 371, (2021). 10.1126/science.aax2656 PMC7900882 33509999

