# Supplementary material for: Astrocyte regional specialization is shaped by postnatal development
Source: bioRxiv. 2024 Oct 12:2024.10.11.617802. Preprint. [Version 1] doi: 10.1101/2024.10.11.617802 (PMC11482951; doi:10.1101/2024.10.11.617802)
Supplement: Supplementary file 1 [file NIHPP2024.10.11.617802v1-supplement-2.pdf]

bioRxiv preprint doi: https://doi.org/10.1101/2024.10.11.617802; this version posted October 12, 2024. The copyright holder for this preprint (which was not certified by peer review) is the author/funder, who has granted bioRxiv a license to display the preprint in perpetuity. It is made available under aCC-BY 4.0 International license.

Supplementary Figures and Movies

Supplementary Figure 1. Summary of sequencing coverage for the cross-region developmental snRNAseq atlas.

Supplementary Figure 2. Distribution of nuclei across cell types, regions, ages, sexes, and study for each species.

Supplementary Figure 3. Leiden cluster annotations and proportions for the cross-region developmental snRNAseq atlas.

Supplementary Figure 4. Dissection strategies for marmoset brains and region reassignment to mitigate cross-region contamination for developing samples

Supplementary Figure 5. Region reassignment for cross-contaminant nuclei in mouse samples.

Supplementary Figure 6. Correlation of pairwise astrocyte rDEG log-fold change between region pairs across development in mouse and marmoset.

Supplementary Figure 7. Validation of marmoset astrocyte rDEG expression in situ using multiplexed FISH.

Supplementary Figure 8. Quantification of selected rDEG and astrocyte subtype marker expression in situ in adult and neonate marmoset.

Supplementary Figure 9. Validation of mouse astrocyte rDEG expression in situ using multiplexed FISH.

Supplementary Figure 10. Quantification of selected rDEG and astrocyte subtype marker expression in situ in adult and neonate mouse.

Moved to Main figures.

Supplementary Figure 11. Astrocyte sub-clustering captures intra-regional heterogeneity in both species.

bioRxiv preprint doi: https://doi.org/10.1101/2024.10.11.617802; this version posted October 12, 2024. The copyright holder for this preprint (which was not certified by peer review) is the author/funder, who has granted bioRxiv a license to display the preprint in perpetuity. It is made available under aCC-BY 4.0 International license.

Supplementary Figure 12. Cell-cell communication analysis for neuron-astrocyte and astrocyte-neuron predicted ligand-receptor pairs across regions and developmental time points in mouse.

Supplementary Figure 13. Pseudotime inference in mouse and marmoset oligodendrocyte lineage and mouse astrocytes.

Supplementary Figure 14. Gene expression signatures underlying the postnatal developmental specification of mouse astrocytes within and across brain regions.

Supplementary Figure 15. Cell type composition across development in both species and cross-species integration with SATURN.

Supplementary Figure 16. Expression of Top2a in astrocytes of the mouse subventricular zone via multiplexed FISH.

Supplementary Figure 17. Additional detail on astrocyte viral labeling and expansion microscopy in mouse and marmoset brain.

Supplementary Figure 18. High-concentration collagenase treatment preserves blood vessel morphology in ExR samples.

Supplementary Movies 1-18. 3D-projected image volumes and binary segmentations of ~4x expanded mouse astrocytes.

<u>Supplementary Tables</u>

Supplementary Table 1. Biological donor information.

Supplementary Table 2. Abundance of cell types and Leiden clusters and descriptions of Leiden clusters in mouse and marmoset.

Supplementary Table 3. Proportional breakdown of each Leiden cluster by age, assigned region, and sex for mouse and marmoset.

Supplementary Table 4. Proportional breakdown of MapMyCells-derived Allen Brain Cell Atlas subclass assignments by Leiden cluster for mouse and marmoset.

Supplementary Table 5. Summary of significant compositional differences in cell type for marmoset as found by scCODA.

Supplementary Table 6. Summary of significant compositional differences in cell type for mouse as found by scCODA.

Supplementary Table 7. Summary of significant compositional differences in Leiden cluster for marmoset as found by scCODA.

Supplementary Table 8. Summary of significant compositional differences in Leiden cluster for mouse as found by scCODA.

Supplementary Table 9. Marmoset rDEGs shared across both replicates for each age.

Supplementary Table 10. WebGestalt enrichment results for marmoset rDEGs by age.

Supplementary Table 11. Mouse rDEGs for each age.

Supplementary Table 12. WebGestalt enrichment results for mouse rDEGs by age.

Supplementary Table 13. Image-level quantification of each rDEG probe for adult and neonate marmoset and mouse and results of statistical tests for mouse.

Supplementary Table 14. CellPhoneDB-generated cell-cell communication results for each region-age combination in mouse and marmoset.

Supplementary Table 15. CellPhoneDB-generated unique ligand-receptor pairs for astrocytes and striatal neurons in mouse and marmoset.

Supplementary Table 16. Marmoset astrocyte, OPC, GABAergic, and glutamatergic neuron aDEGs for each brain region.

Supplementary Table 17. Mouse astrocyte, OPC, GABAergic, and glutamatergic neuron aDEGs for each brain region.

bioRxiv preprint doi: https://doi.org/10.1101/2024.10.11.617802; this version posted October 12, 2024. The copyright holder for this preprint (which was not certified by peer review) is the author/funder, who has granted bioRxiv a license to display the preprint in perpetuity. It is made available under aCC-BY 4.0 International license.

Supplementary Table 18. Shared and species-unique cortex-thalamus astrocyte rDEGs for each developmental timepoint in mouse and marmoset.

Supplementary Table 19. Shared and species-unique fetal-late adolescent astrocyte aDEGs for each region in mouse and marmoset.

Supplementary Table 20. sDEGs for each supercluster.

Supplementary Table 21. Telencephalic-specific, diencephalic-specific, and shared astrocyte sDEGs.

Supplementary Table 22. Summary statistics for quantification of mouse astrocyte morphology across brain regions.

Supplementary Table 23. Proteins stained in each round of mouse astrocyte multiExR.

Supplementary Table 24. Blocking strategy for marmoset region dissection based on custom brain matrix.

Supplementary Table 25. Sequencing coverage statistics for all 10x Chromium reactions.

Supplementary Table 26. Table used to convert between mouse, marmoset, and human gene IDs for 1:1 orthologs.

Supplementary Table 27. List of reagents, including vendor and product information, used for experiments.

Supplementary Table 28. Expansion factor measurements for mouse and marmoset ExR samples.

Supplementary Table 29. Notes on each ~4x expanded mouse astrocyte imaged (Fig. 7).

bioRxiv preprint doi: https://doi.org/10.1101/2024.10.11.617802; this version posted October 12, 2024. The copyright holder for this preprint (which was not certified by peer review) is the author/funder, who has granted bioRxiv a license to display the preprint in perpetuity. It is made available under aCC-BY 4.0 International license.

Supplementary Figure 1. Summary of sequencing coverage for the cross-region developmental snRNAseq atlas. A, Violin plot showing the estimated population distribution of sense reads per rough quality control (QC)-passed (low-UMI and low-gene but not doublet removal, see **Methods**) for all nuclei across all 144 10x Chromium reactions sequenced in the current study (not including adult marmoset data from our previous study[7]). Dashed lines indicate the median and quartiles. **B**, Violin plot (as described for **(A)**) of the median number of unique molecular identifiers (UMIs) per nucleus as described in **(A)** (n = 144 10x Chromium reactions). **C,** Same as **(B)**, for the median number of genes per nucleus. **D**, Same as **(C)**, for sequencing saturation as calculated by 10x Cell Ranger software (v7.1). **E**, Same as **(D)**, for the fraction of reads mapped antisense to genes, as calculated by 10x Cell Ranger software (v7.1). **F**, Number of nuclei called by 10x Cell Ranger software before (pre-QC) and after (post-QC) rough QC (low-UMI and low-gene but not doublet removal, see **Methods**). **G,** Violin plot (scanpy's default) showing the estimated distribution of the number of UMIs (left) and genes (right) for full QC-passed marmoset nuclei, as presented in the main figures, grouped by cell type. **H**, Same as **(G)**, for mouse nuclei.

Supplementary Figure 2. Distribution of nuclei across cell types, dissected regions, ages, sexes, and study for each species. Integrated UMAP embedding of marmoset (**A**, 881,832) or mouse (**B**, 597,668) nuclei from PFC, motor cortex, striatum, and thalamus (as dissected, before reassignment) across all developmental time points assayed and a randomly downsampled portion of adult nuclei from our previous study[7] colored by **A(i)** study (present, "schroeder" or previous, "krienen"), **A(ii)** and **B(i)** sex, or **A(iii)** and **B(ii)** biological replicate (i.e., donor). **C-D**, Stacked bar plots showing the number or proportion of full QC-passed nuclei for each **(i)** dissected brain region, **(ii)** age (number of nuclei), **(iii)** age (proportion of nuclei) **(iv)** sex (proportion of nuclei) and **(v)** biological donor, broken down by assigned cell type for **(C)** marmoset and **(D)** mouse. **E-G**, Log2-fold change in cell type composition as estimated by single-cell compositional data analysis (scCODA)[37] for the coefficients on **(E)** age (reference = P32 or 14 months), **(F)** region (reference = cortex), and **(G)** male sex (reference = female) for **(i)** marmoset and **(ii)** mouse. Only fold-changes for credible effects (i.e., significantly nonzero coefficient) are shown. We obtained 597,668 mouse nuclei and 881,832 marmoset nuclei, which were composed of 12 broad cell classes: glutamatergic excitatory neurons (44.7% in mouse, 29.7% in marmoset), GABAergic inhibitory neurons (21.8% in mouse, 25.2% in marmoset), neurons belonging to mixed glutamatergic and GABAergic clusters (5.54% in mouse, 6.24% in marmoset), astrocytes (11.5% in mouse, 11.7% in marmoset), oligodendrocytes (8.41% in mouse, 14.0% in marmoset), oligodendrocyte progenitor cells (OPCs, 3.91% in mouse, 5.35% in marmoset), microglia and macrophages (2.98% in mouse, 3.45% in marmoset), mural cells (0.28% in mouse, 0.51% in marmoset), endothelial cells (0.26% in mouse, 1.22% in marmoset), vascular-associated fibroblasts (0.62% in mouse, 1.05% in marmoset), ependymal cells (0% in mouse, 1.11% in marmoset), and choroid plexus cells (0.12% in mouse, 0.45% in marmoset).

Supplementary Figure 3. Leiden cluster annotations and proportions for the cross-region developmental snRNAseq atlas. A, Integrated UMAP embedding of marmoset (**A**, 881,832) or mouse (**B**, 597,668) nuclei from PFC, motor cortex, striatum, and thalamus (as dissected, before reassignment) across all developmental time points assayed and a randomly downsampled portion of adult nuclei from our previous study[7] colored by **(i)** Leiden-algorithm determined cluster annotation (hereafter, Leiden cluster), **(ii)** dissected region and **(iii)** developmental time point. **B**, Stacked bar plot showing the proportion of all marmoset nuclei from each developmental time point (see legend in **A(iii)**). **C,** Stacked bar plot showing the proportion of each Leiden cluster from each developmental time point for a hand-selected set of 10 clusters that are developmentally enriched or depleted. **D,** Stacked barplots showing the number of nuclei assigned to each of the top 5 most abundant Allen Brain Cell Atlas mouse whole-

bioRxiv preprint doi: https://doi.org/10.1101/2024.10.11.617802; this version posted October 12, 2024. The copyright holder for this preprint (which was not certified by peer review) is the author/funder, who has granted bioRxiv a license to display the preprint in perpetuity. It is made available under aCC-BY 4.0 International license.

brain atlas class or subclass using MapMyCells[36] for **(i)** excitatory neurons **(ii)** inhibitory neurons and **(iii)** astrocytes, broken down by dissected brain region. **E-H,** Same as **A-D,** for mouse.

Supplementary Figure 4. Dissection strategies for marmoset brains and region reassignment to mitigate cross-region contamination for developing samples. A, Dissection strategy for 7-month, 14-month, and aged marmosets used in this study. **(i)** Ventral view of brain from female 14-month marmoset donor 21-164 in brain matrix. **(ii)** Blades positioned in blade holder to create desired tissue slabs for dissection. Arrows on the blade holder indicate anterior direction. **(iii)** Tissue slab for prefrontal cortex (PFC) dissection, with red arrows indicating PFC. Some white matter was removed, and then the entire slab was frozen for nuclei isolation. **(iv)** One out of two tissue slabs used for striatum dissection, with yellow arrows indicating striatum (not yet dissected). **(v)** Tissue slab used for motor cortex dissection, with blue arrows indicating where motor cortex was removed (already removed in this photo). **(vi)** Tissue slab used for thalamus dissection, with dark green arrows indicating the thalamus (already dissected from the left hemisphere, but not yet removed). **B,** Dissection strategy for GD135 marmoset (similar for neonate). **(i)** Dorsal view of brain from female GD135 donor 19-221_fetus1. **(ii)** Prefrontal cortex as grossly dissected from lateral surface of the brain. **(iii)** Two slabs cut from the bissected region spanning approximately from the anterior beginning of the temporal lobe to its posterior end, used to dissect the striatum (slab 1, S1) and thalamus (slab 2, S2). **(iv)** Striatum as dissected from slab 1 (S1). **(v)** Thalamus as dissected from slab 2 (S2). **C**, Analysis of dissection cross-contamination in marmoset (all ages, but only nuclei from GD135 and neonate were reassigned) using medium spiny neurons (MSNs). **(i-v)** UMAP projection of marmoset MSNs colored by **(i)** *DRD1* expression in logCPM units, **(ii)** *DRD2* expression in logCPM units, **(iii)** dissected brain region, **(iv)** reassigned brain region, and **(v)** age. **(iii)** Shows contamination of thalamus in striatal dissections for GD135 and neonate samples. **D,** Same as **(C)**, for marmoset thalamic excitatory neurons, with **(i)** showing *SLC17A6* expression and **(ii)** showing *TCF7L2* expression. **(iii)** Shows contamination of striatum in thalamic dissections for GD135 and neonate samples. **E,** Analysis of dissection cross-contamination in marmoset (all ages, but only nuclei from GD135 and neonate were reassigned) astrocytes of all clusters. UMAP projection of marmoset astrocytes colored by **(i)** *SLC1A3* expression, **(ii)**, *FOXG1* expression, **(iii)** dissected brain region, **(iv)** reassigned brain region, **(v)** age, and **(vi)** ABCA subclass, as determined using MapMyCells.

Supplementary Figure 5. Region reassignment for cross-contaminant nuclei in mouse samples. A, Analysis of dissection cross-contamination in mouse (all ages) using medium spiny neurons (MSNs). **(i-v)** UMAP projection of mouse MSNs colored by **(i)** *Drd1* expression in logCPM units, **(ii)** *Drd2* expression in logCPM units, **(iii)** dissected brain region, **(iv)** reassigned brain region, and **(v)** age. **B,** Same as **(A)**, for mouse thalamic excitatory neurons, with **(i)** showing *Slc17a6* expression and **(ii)** showing *Tcf7l2* expression. **C,** Same as **(A),** for immature mouse MSNs, showing a higher degree of striatum-thalamus cross-contamination in E18.5 mouse samples. **D,** Same as **(A-C)**, for immature mouse cortical neurons, with **(i)** showing *Slc17a7* expression and **(ii)** showing *Satb2* expression. **(iii)** Illustrates cortical contamination of striatal and thalamic dissections in the mouse E18.5 data. **E,** Analysis of dissection cross-contamination in mouse (all ages) astrocytes of all clusters. UMAP projection of mouse astrocytes colored by **(i)** *Slc1a3* expression, **(ii)**, *Foxg1* expression, **(iii)** dissected brain region, **(iv)** reassigned brain region, **(v)** age, and **(vi)** ABCA subclass, as determined using MapMyCells.

Supplementary Figure 6. Correlation of pairwise astrocyte rDEG log-fold change between region pairs across development in mouse and marmoset. A, Scatterplot of log fold-change (logFC) difference in expression in marmoset GD135 astrocytes for genes meeting minimum metacell expression criteria (relaxed from the criteria for rDEGs shown in **Figs. 2-3**, see **Methods**) for 2 different region pairs:

bioRxiv preprint doi: https://doi.org/10.1101/2024.10.11.617802; this version posted October 12, 2024. The copyright holder for this preprint (which was not certified by peer review) is the author/funder, who has granted bioRxiv a license to display the preprint in perpetuity. It is made available under aCC-BY 4.0 International license.

(i) cortex-striatum vs. cortex-thalamus (a negative value indicates downregulation in cortex), **(ii)** striatum-thalamus vs. striatum-cortex (a negative value indicates downregulation in striatum), and **(iii)** thalamus-striatum vs. thalamus-cortex (a negative value indicates downregulation in thalamus). Light green dots indicate genes who meet rDEG (magnitude of logFC > 0.5) criteria for one region pair, and dark green dots indicate genes who meet rDEG criteria for both region pairs. In marmoset, logFC is averaged across replicates. r, Pearson's correlation coefficient calculated using all the points shown. **B,** Same as **(A)** for 7-month marmoset. **C,** Same as **(A)** for 30-month marmoset. **D**, Same as **(A)** for E18.5 mouse. **E**, Same as **(A)** for P14 mouse. **F**, Same as **(A)** for P90 mouse.

Supplementary Figure 7. Validation of marmoset astrocyte rDEG expression in situ using multiplexed FISH. A, Composite images of registered (across imaging rounds), cropped maximum intensity projections of imaged fields of view in the **(i)** PFC **(ii)** striatum and **(iii)** thalamus in the one female adult marmoset, where each channel is one cell type marker. *SLC1A3* was used to select astrocytes for quantification, while *SLC17A6* and *SLC17A7* were used to exclude neurons (see **Methods**). Scale bar, 50μm. **B,** Single-channel and composite images of telencephalic astrocyte rDEGs for 3 exemplary astrocytes (low autofluorescence and high signal-to-noise) indicated by the white box corners in the fields of view shown in **(A)**. **C,** Same as **(B)**, for diencephalic astrocyte rDEGs. Scale bar, 5μm. **D-F,** Same as **A-C**, for one male neonate marmoset. Gain was manually scaled by setting minimum and maximum intensity values, using the same values across regions within each age, except for *SLC1A3* in neonate*,* for which we reduced the maximum intensity value in the thalamus (vs. cortex and striatum), to account for lower expression in order to visualize its expression in astrocytes.

Supplementary Figure 8. Quantification of selected rDEG and astrocyte subtype marker expression *in situ* in adult and neonate marmoset. A, Mean expression (in logCPM from the snRNAseq data) of selected rDEG (*DNM3, KNC7, SPARC, CSMD1, KCND2*) and astrocyte subtype (*GFAP*) genes by region in the **(i)** adult (29-32 month) marmoset and **(ii)** neonate marmoset (with the addition of *FOXG1*, which we excluded from **(i)** due to low signal-to-noise that led to false positive identification of signal in the thalamus, see **Methods**). **B, (i)** Line plots showing fraction of astrocytes positive for these genes in PFC (red), striatum (yellow), and thalamus (green) from FISH data (**Fig. S7**) for 2 adult female marmosets. **(ii)** Line plots showing mean fraction of astrocytes covered by probes for the genes in **(i)** in PFC (red), striatum (yellow), and thalamus (green) from FISH data (**Fig. S7**) for 2 adult female marmosets. See **Methods** for details on quantification, which was performed semi-automatically using CellProfiler. Colored dots connected by lines are data originating from the same marmoset donor: gray lines indicate one donor, while black lines indicate another. **C,** Same as **(B)**, for two neonate marmoset donors, one male and one female.

Supplementary Figure 9. Validation of mouse astrocyte rDEG expression in situ using multiplexed FISH. A, Composite images of registered (across imaging rounds), cropped maximum intensity projections of imaged fields of view in the **(i)** PFC **(ii)** striatum and **(iii)** thalamus in the one female P90 mouse, where each channel is one cell type marker. *Slc1a3* was used to select astrocytes for quantification, while *Slc17a6* and *Slc17a7* were used to exclude neurons (see **Methods**). Scale bar, 50μm. **B,** Single-channel and composite images of telencephalic astrocyte rDEGs for 3 exemplary astrocytes indicated by the white box corners in the fields of view shown in **(A)**. **C,** Same as **(B)**, for diencephalic rDEGs (*Sparc, Kcnd2*) and other astrocyte subtype markers (*Gfap* and *Top2a*). Scale bar, 5μm. **D-F,** Same as **A-C**, for one male P4 mouse. Gain was manually scaled by setting minimum and maximum intensity values, using the same values across regions within each age.

bioRxiv preprint doi: https://doi.org/10.1101/2024.10.11.617802; this version posted October 12, 2024. The copyright holder for this preprint (which was not certified by peer review) is the author/funder, who has granted bioRxiv a license to display the preprint in perpetuity. It is made available under aCC-BY 4.0 International license.

Supplementary Figure 10. Quantification of selected rDEG and astrocyte subtype marker expression *in situ* in adult and neonate mouse. A, Mean expression (in logCPM from the snRNAseq data) of selected rDEG (*Clmn, Slco1c1, Csmd1, Sparc,* and *Kcnd2*) and astrocyte subtype (*Gfap, Top2a*) genes by region in the **(i)** adult (P90) and **(ii)** neonate (P4) mouse. **B, (i)** Bar plots showing the mean fraction of astrocytes positive for these genes in PFC (red), striatum (yellow), and thalamus (green) from FISH data in P90 mice (**Fig. S9**, n = 4 mice, 2 of each sex, data point shows average of 2 tissue sections each). **(ii)** Bar plots showing the mean fraction of astrocytes covered by probes for the genes in **(i)** in PFC (red), striatum (yellow), and thalamus (green) from FISH data (**Fig. S9**, n = 4 mice, 2 of each sex, data point shows average of all astrocytes from 2 tissue sections each). Error bars indicate standard error of the mean. See **Methods** for details on quantification, which was performed semi-automatically using CellProfiler. Statistical significance was determined in GraphPad Prism using a repeated-measures one-way ANOVA, followed by Tukey's multiple comparisons test (see **Table S13** for full statistics for each gene). ns, non significant, *p≤0.05, **p≤0.01, ***p≤0.001, ****p≤0.0001. **C,** Same as **(B)**, for P4 mice (n = 4, 2 of each sex, data point shows average across 2 tissue sections each).

Supplementary Figure 11. Astrocyte sub-clustering captures intra-regional heterogeneity in both species. A, UMAP embedding of 36,136 marmoset cortical astrocytes from all developmental time points colored by **(i)** subcluster (see **Methods**), **(ii)** age, or **(iii)** *FOXG1, GFAP, AQP4, ID3, CRYM,* or *SPARC* expression in logCPM units. *FOXG1* marks telencephalic astrocytes, *GFAP, AQP4*, and *ID3* mark fibrous and interlaminar astrocytes[53], *CRYM* marks striatal astrocytes[54], and *SPARC* marks thalamic astrocytes[21]. **B,** Same as **(A)** for 29,931 marmoset striatal astrocytes. **C,** Same as **(A)** for 35,493 marmoset thalamic astrocytes. There are 1,380 *FOXG1*+ astrocytes in the thalamus clusters, ~81% of which are assigned to the non-telencephalic astrocyte ABCA subclass, most but not all of which come from the donors from the current study, and may reflect contamination from neighboring regions such as globus pallidus or septum. **D-F**, same as **(A-C)** for mouse astrocytes: **(D)** 25,558 from cortex **(E)** 19,687 from striatum, and **(F)** 23,240 in thalamus. *Top2a* marks immature and SVZ rostral migratory stream astrocytes.

Supplementary Figure 12. Cell-cell communication analysis for neuron-astrocyte and astrocyte-neuron predicted ligand-receptor pairs across regions and developmental time points in mouse. A, Dot plot showing magnitude and specificity of the top 25 near-unique (shared with at most one other neuronal cluster) CellPhoneDB-predicted **(i)** neuron-astrocyte and **(ii)** astrocyte-neuron ligand receptor pairs for the most abundant astrocyte and neuronal Leiden clusters in the neonate mouse thalamus. The source cell (top of the plot) expresses the ligand (left side of arrow on the row labels), while the target cell (bottom of the plot) expresses the receptor (right side of arrow on the row labels). The color of the dot indicates ligand-receptor expression magnitude ("Lr_means", calculated as the average of the mean expression of the ligand in the source group and the mean expression of the receptor in the target group), while the size of the dot is inversely related to the p-value on ligand-receptor expression sensitivity (see **Methods**). Glut-Hab, glutamatergic habenula; Glut-Re-XI, glutamatergic reunions/xiphoid nucleus; Glut-PF, glutamatergic parafascicular nucleus; Gad-TRN, GABAergic thalamic reticular nucleus. **B,** Same as **(A)**, for the P90 mouse thalamus, with the uniqueness criteria relaxed to up to 3 neuronal clusters. Glut-ATN, glutamatergic anterior thalamic nuclei. **C,** UpSet plot showing the number of overlapping neuron-astrocyte predicted ligand-receptor pairs between regions, from the most abundant neuronal and astrocyte subtypes in each region for **(i)** fetal and **(ii)** late adolescent mouse. For cortex (purple), glutamatergic L2/3IT neurons to cortical astrocytes; striatum (yellow), *Drd1*+ medium spiny neurons to striatal telencephalic astrocytes; and thalamus (green) thalamic glutamatergic neurons to thalamic

bioRxiv preprint doi: https://doi.org/10.1101/2024.10.11.617802; this version posted October 12, 2024. The copyright holder for this preprint (which was not certified by peer review) is the author/funder, who has granted bioRxiv a license to display the preprint in perpetuity. It is made available under aCC-BY 4.0 International license.

astrocytes. **D**, UpSet plot (as in **(C)**) showing the number of overlapping cortical glutamatergic L2/3IT neuron to cortical astrocyte predicted ligand-receptor pairs between ages. **E,** Examination of unique predicted ligand-receptor interactions between individual striatal neuron Leiden clusters and striatal astrocytes. **(i)** Pie chart showing the proportion of unique striatal neuron to striatal astrocyte predicted ligand-receptor pairs for each Leiden cluster of striatal neurons. *CHAT+*, cholinergic interneurons (ChINs); Imm *Top2a+,* immature Top2a+; MGE, medial ganglionic eminence; LGE, lateral ganglionic eminence. **(ii)** Dot plot showing expression of selected unique (not significantly enriched in other striatal neurons) ChIN-astrocyte ligand-receptor pairs in ChINs (top row), striatal astrocytes (second row), and other striatal neurons (bottom 8 rows). Ligands are odd gene names, starting from *Nlgn2* and receptors are the even gene names on their right, starting with *Nrxn1*. The full list of unique ChIN-astrocyte predicted ligand-receptor pairs and their associated mean expression and CellPhoneDB p-values are provided in **Table S15.**

Supplementary Figure 13. Pseudotime inference in mouse and marmoset oligodendrocyte lineage and mouse astrocytes. A, Integrated UMAP embeddings of 100,000 (out of 170,786, randomly downsampled) marmoset oligodendrocyte lineage cells colored by **(i)** Palantir-predicted pseudotime **(ii)** developmental time point **(iii)** brain region and **(iv)** Leiden cluster assignment. **B,** Violin plot (scanpy's default) showing the estimated distribution of pseudotime values for the oligodendrocyte lineage cells in **(A)** grouped brain region and developmental time point. Color scheme for ages as in **(ii)**, with vertical dashed lines indicating divisions between time points. **C-D,** Same as **(A-B)**, for 73,638 mouse oligodendrocyte lineage cells. **E,** Integrated UMAP embeddings of 68,485 mouse astrocytes colored by **(i)** Palantir-predicted pseudotime **(ii)** developmental time point **(iii)** brain region and **(iv)** Leiden cluster assignment. **F,** Violin plot (scanpy's default) showing the estimated distribution of pseudotime values for the astrocytes in **(E)** grouped by brain region and developmental time point. Color scheme for ages as in **(ii)**.

Supplementary Figure 14. Gene expression signatures underlying the postnatal developmental specification of mouse astrocytes within and across brain regions. A, Heatmaps (rows corresponding to nuclei and columns to gene) showing expression in logCPM of astrocyte age differentially expressed genes (aDEGs) in astrocytes from **(i)** cortex, **(ii)** striatum, and **(iii)** thalamus, grouped by developmental time point as indicated on the left of the heatmap. The strategy for calculating aDEGs is schematized on the left. **B**, UpSet plot showing the number of overlapping P4 vs. P90 astrocyte aDEGs between cortex, striatum, and thalamus. The colored dots below each vertical bar indicate which region(s) share that set of aDEGs, while the colored horizontal bars indicate the total number of cortex-thalamus aDEGs for each region. **C**, UpSet plot (as in **(B)**) showing the number of overlapping P4 vs. P90 cortical astrocyte aDEGs between OPCs (light blue), astrocytes (dark blue), excitatory neurons (pink), and inhibitory neurons (gray). **D,** Matrix plot showing mean expression of selected cortex group AS-RS aDEGs (rows) in mouse astrocytes grouped by region and developmental time point (columns, blocked by region first and then by increasing age within each region block). Expression units of mean logCPM are standardized between 0 and 1 by subtracting the minimum and dividing by the maximum for each trait.

Supplementary Figure 15. Cell type composition across development in both species and cross-species integration with SATURN. A, Line graphs showing the proportions of major cell classes in the marmoset (solid lines) and mouse (dashed lines) in log10 scale across developmental time points in the **(i)** cortex, **(ii)** striatum, and **(iii)** thalamus. **B,** scANVI-integrated UMAP embeddings of downsampled marmoset and mouse nuclei, colored by **(i)** supercluster, **(ii)** species, and **(iii)** age. **C,** SATURN-

bioRxiv preprint doi: https://doi.org/10.1101/2024.10.11.617802; this version posted October 12, 2024. The copyright holder for this preprint (which was not certified by peer review) is the author/funder, who has granted bioRxiv a license to display the preprint in perpetuity. It is made available under aCC-BY 4.0 International license.

integrated UMAP embeddings of downsampled marmoset and mouse nuclei, colored by **(i)** supercluster, **(ii)** species, and **(iii)** age. **D,** SATURN-integrated UMAP embeddings of marmoset and mouse astrocytes, colored by **(i)** supercluster, **(ii)** species, **(iii)** age, and **(iv)** region.

Supplementary Figure 16. Expression of *Top2a* in astrocytes of the mouse subventricular zone via multiplexed FISH. A, Composite image of a registered (across imaging rounds), cropped maximum intensity projection of imaged fields of view in the **(i)** PFC **(ii)** striatum and **(iii)** thalamus in one female P90 mouse. Scale bar, 200µm. **B,** Single-channel and composite images of the boxed regions of the subventricular zone in **(A)**. Scale bar, 20µm. LV, lateral ventricle. SVZ, subventricular zone. CC, corpus callosum. Contrast was manually scaled by setting minimum and maximum intensity values.

Supplementary Figure 17. Additional detail on astrocyte viral labeling and expansion microscopy in mouse and marmoset brain. A, Maximum intensity projections of 3D segmentations of examples of astrocytes exhibiting atypical morphologies in the mouse brain. The abbreviated label indicates brain region of origin. The leftmost striatal astrocyte was excluded from our quantification results, as its volume was cut off on the z-axis. Scale bar, 10µm. **B, (i)** Maximum intensity projection of a background-subtracted image of a ~4x expanded astrocyte in the thalamus with its soma located directly on a large blood vessel, co-stained with GFP, Gfap, and the blood vessel marker Lectin. Scale bar, 10µm. **(ii)** Maximum intensity projection of the 3D binary segmentation of the astrocyte in **(i)**. Because of its irregular morphology, this astrocyte was excluded from our quantification results. **C,** Maximum intensity projections of the full fields of view of the registered multiExR image volumes shown in **Fig. 7G**, scaled to the same min-max intensity ranges as in **Fig. 7G**. Scale bar, 2µm. White arrows indicate loop-like structures on astrocyte processes. **D, (i)** Sagittal 150µm thick brain slice from the adult female marmoset (19-241) whose astrocytes are shown in **Fig. 7H-I**, after pre-expansion staining and before dissection and processing for ExR. **(ii-iv)** Dissected regions of interest after pre-expansion staining for GFP, as visible through the eyepiece on a dissecting microscope under blue light illumination. White arrows indicate tissue pieces processed for ExR. **E,** Brain-wide astrocyte labeling in a second adult marmoset processed with conventional fixation in 4% PFA only. **(i)** Single-plane epifluorescence image of a sagittal brain slice from a different adult female marmoset injected systemically with AAV BI103 gfaABC1D-eGFP after GFP staining (80µm thickness, donor 18-169, see **Methods**). Scale bar, 2mm. Contrast was manually adjusted in Fiji. **(ii)** Extended focus projection (TissueGnostics software) of a field of view in the **(ii)** PFC and **(iii)** thalamus of the marmoset brain shown in **(i)**, co-stained for GFP, the neuronal marker NeuN **(1)** or the oligodendrocyte lineage marker Olig2 **(2)**, and GFAP. Contrast was manually adjusted in Fiji, with equal min-max for the GFP channel across all regions in both sets.

Supplementary Figure 18. High-concentration collagenase treatment preserves blood vessel morphology in ExR samples. A, (i) Maximum intensity projection cropped field of view obtained at 4x magnification of ~4x expanded tissue from an Aldh1l1-Cre mouse injected at P1-P2 with PhP.eB CAG-FLEX-GFP and perfused with ExR fixative after 5 weeks. Tissue was sectioned at 80µm, pre-stained for GFP and processed according to the original ExR protocol without collagenase treatment[34]. Blood vessels (cyan, Lectin stain) appear fragmented. This animal is from a litter of mice from a separate experiment whose data was not included in the current paper. **(ii),** Same as **(i)**, for an animal from the batch used in the current paper, sectioned at 150µm, pre-stained for GFP, and treated overnight with collagenase VII at 0.5kU/mL (see **Methods**). Blood vessels appear continuous. Both fields of view are from the thalamus. Scale bar, 420µm in physical units (~100-120µm in biological units, assuming a 3.5-4.2x expansion factor; the expansion factor was lower in the experiment in **(i)**). **B,** Maximum intensity projections of 40x magnification astrocyte fields of view in the **(i)** PFC, **(ii)** striatum, and **(iii)** thalamus

bioRxiv preprint doi: https://doi.org/10.1101/2024.10.11.617802; this version posted October 12, 2024. The copyright holder for this preprint (which was not certified by peer review) is the author/funder, who has granted bioRxiv a license to display the preprint in perpetuity. It is made available under aCC-BY 4.0 International license.

from the experiment shown in **(A)(i)**. Fields of view in **(i)** and **(ii)** are from one male mouse, while **(iii)** is from a female mouse. Contrast was manually adjusted in Fiji at or above 35% saturation to show similar GFP brightness across regions. Despite fragmented blood vessels, astrocyte morphology appears almost entirely continuous (compare to **Fig. 7C-D**), except at end feet directly touching blood vessels. Smaller white arrows indicate breaks within blood vessels where astrocyte end feet may exhibit possible local distortion, while larger white arrowheads indicate unbroken blood vessels, where astrocyte processes appear continuous and in good registration with the vessel. Scale bar, 35µm in physical units (~10µm in biological units, assuming a 3.5x expansion factor).

SUPPLEMENTARY FIGURE 1

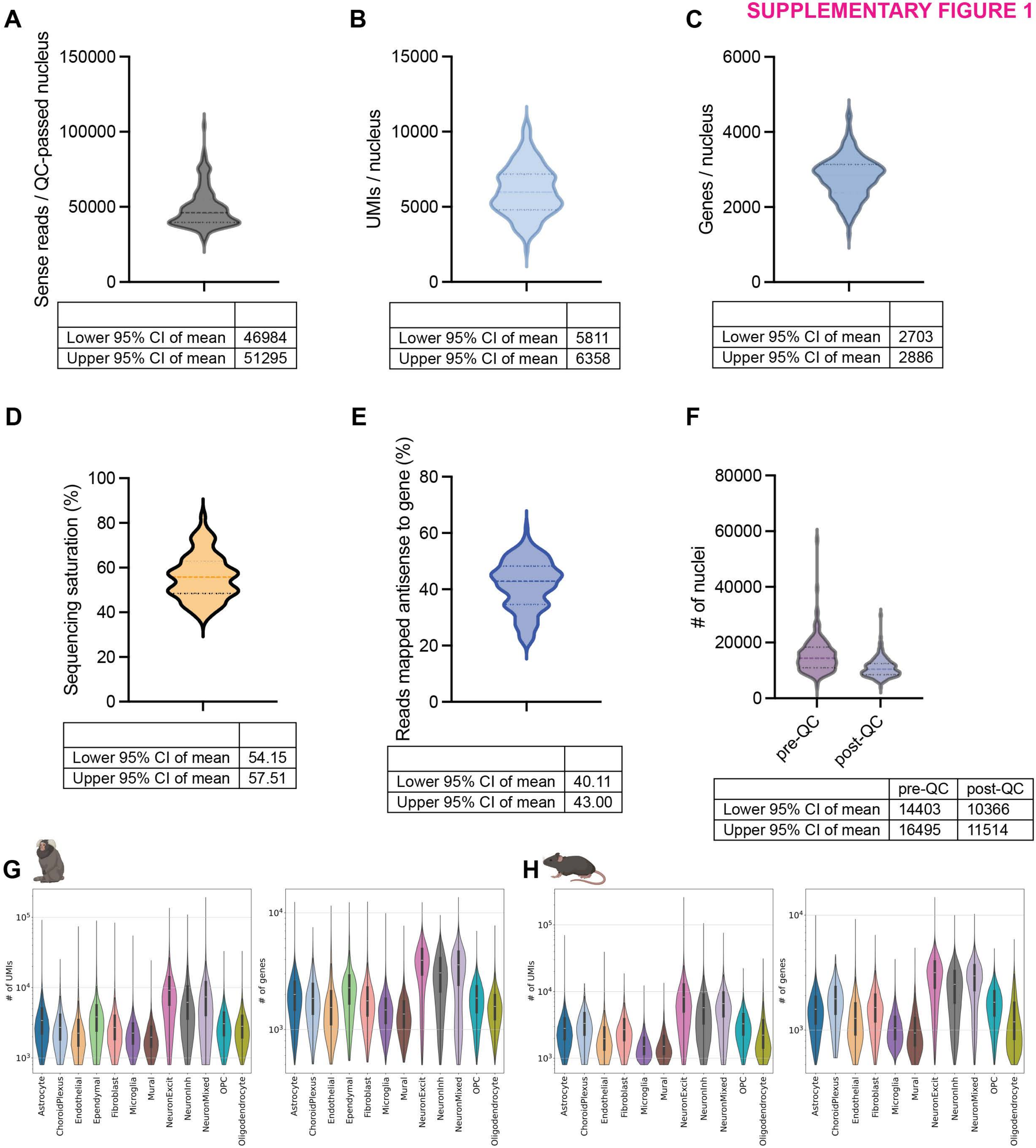

A

Lower 95% CI of mean	46984
Upper 95% CI of mean	51295

B

Lower 95% CI of mean	5811
Upper 95% CI of mean	6358

C

Lower 95% CI of mean	2703
Upper 95% CI of mean	2886

D

Lower 95% CI of mean	54.15
Upper 95% CI of mean	57.51

E

Lower 95% CI of mean	40.11
Upper 95% CI of mean	43.00

F

	pre-QC	post-QC
Lower 95% CI of mean	14403	10366
Upper 95% CI of mean	16495	11514

bioRxiv preprint doi: https://doi.org/10.1101/2024.10.11.617682; this version posted October 12, 2024. The copyright holder for this preprint (which was not certified by peer review) is the author/funder, who has granted bioRxiv a license to display the preprint in perpetuity. It is made available under aCC-BY 4.0 International license.

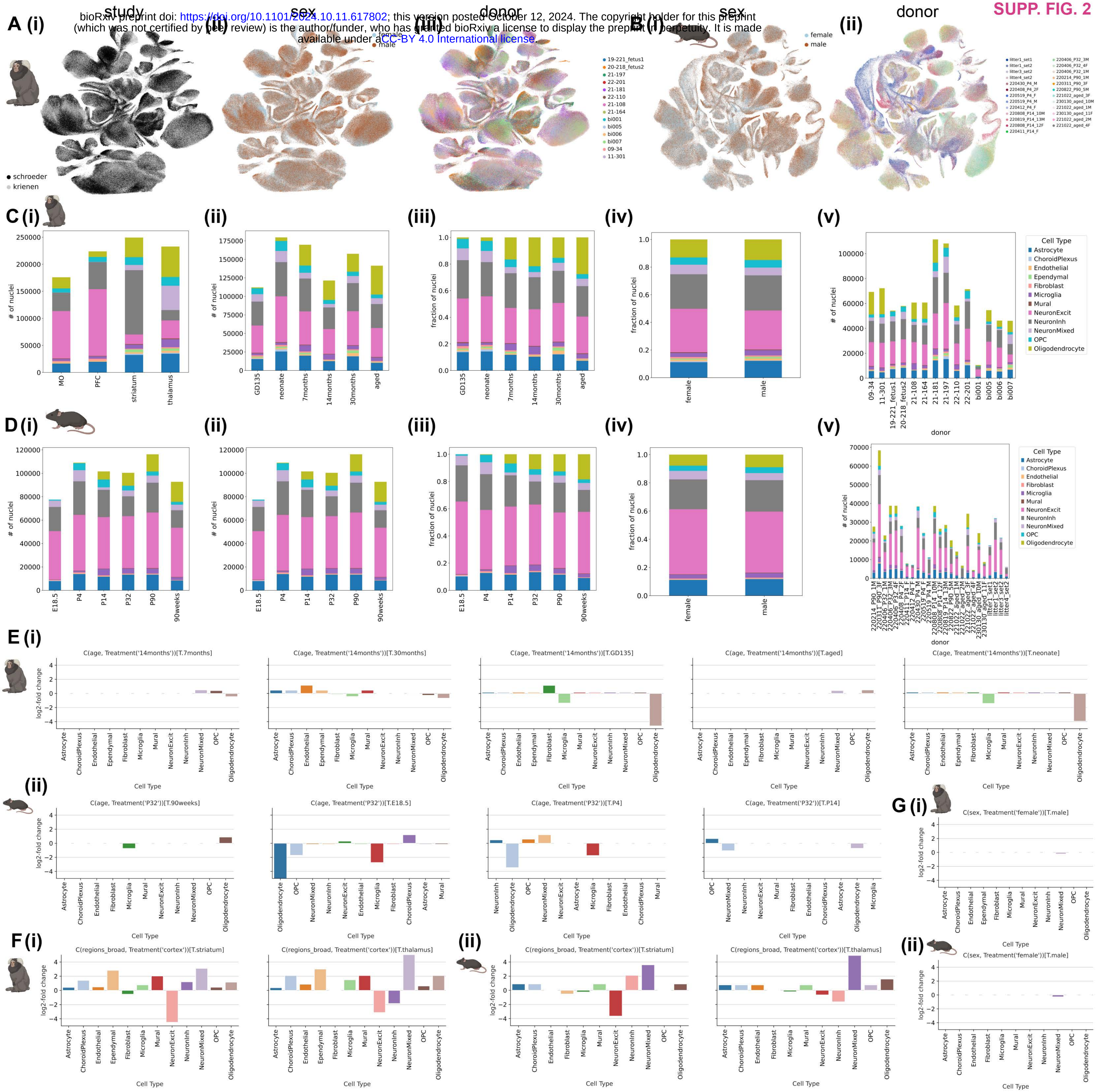

SUPP. FIG. 3

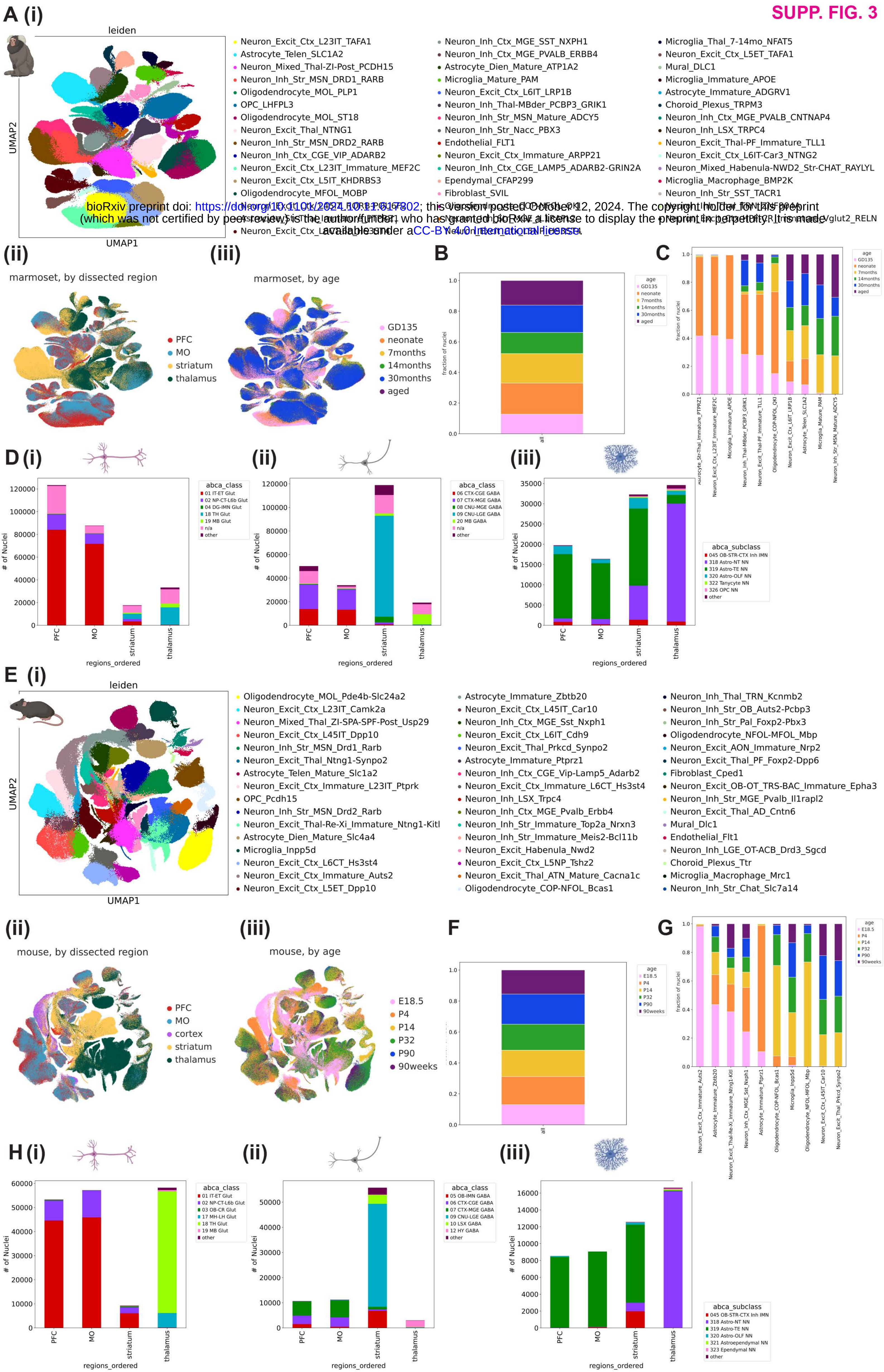

bioRxiv preprint doi: https://doi.org/10.1101/2024.10.11.617802; this version posted October 12, 2024. The copyright holder for this preprint (which was not certified by peer review) is the author/funder, who has granted bioRxiv a license to display the preprint in perpetuity. It is made available under aCC-BY 4.0 International license.

bioRxiv preprint doi: https://doi.org/10.1101/2024.10.11.617802; this version posted October 12, 2024. The copyright holder for this preprint (which was not certified by peer review) is the author/funder, who has granted bioRxiv a license to display the preprint in perpetuity. It is made available under aCC-BY 4.0 International license.

SUPP. FIG. 4

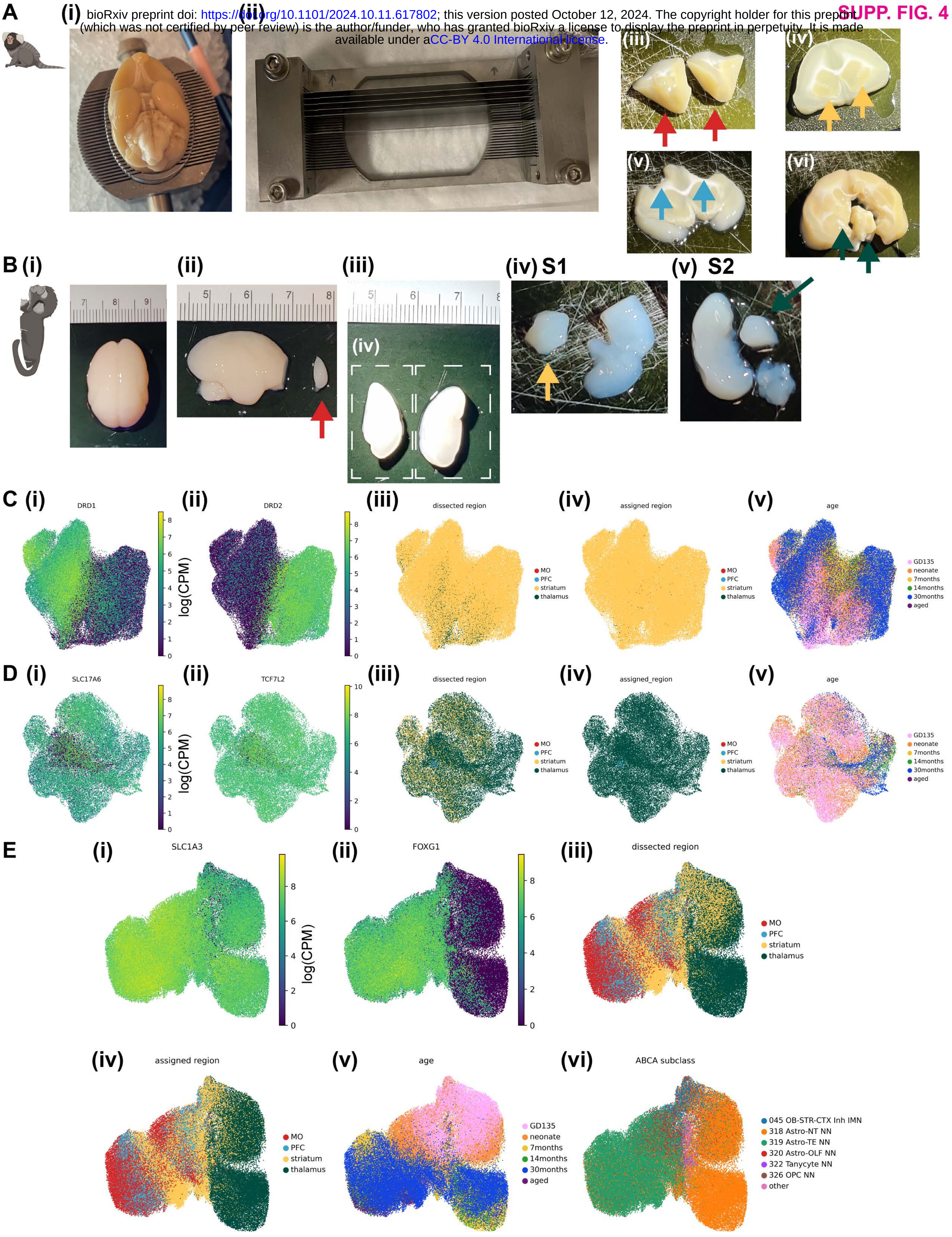

bioRxiv preprint doi: https://doi.org/10.1101/2024.10.11.617802; this version posted October 12, 2024. The copyright holder for this preprint (which was not certified by peer review) is the author/funder, who has granted bioRxiv a license to display the preprint in perpetuity. It is made available under aCC-BY 4.0 International license.

SUPP. FIG. 5

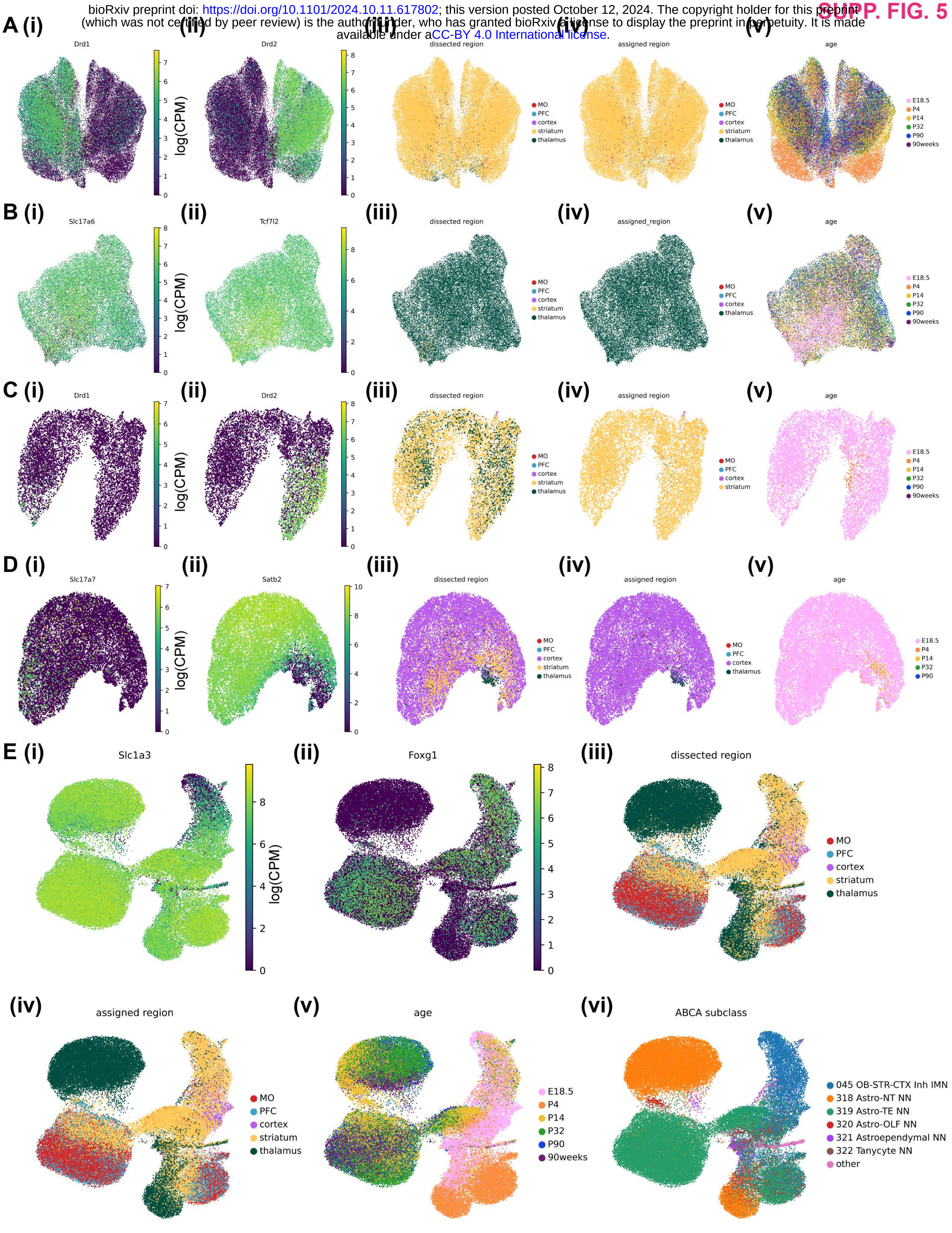

bioRxiv preprint doi: https://doi.org/10.1101/2024.10.11.617802; this version posted October 12, 2024. The copyright holder for this preprint (which was not certified by peer review) is the author/funder, who has granted bioRxiv a license to display the preprint in perpetuity. It is made available under aCC-BY 4.0 International license.

SUPP. FIG. 6

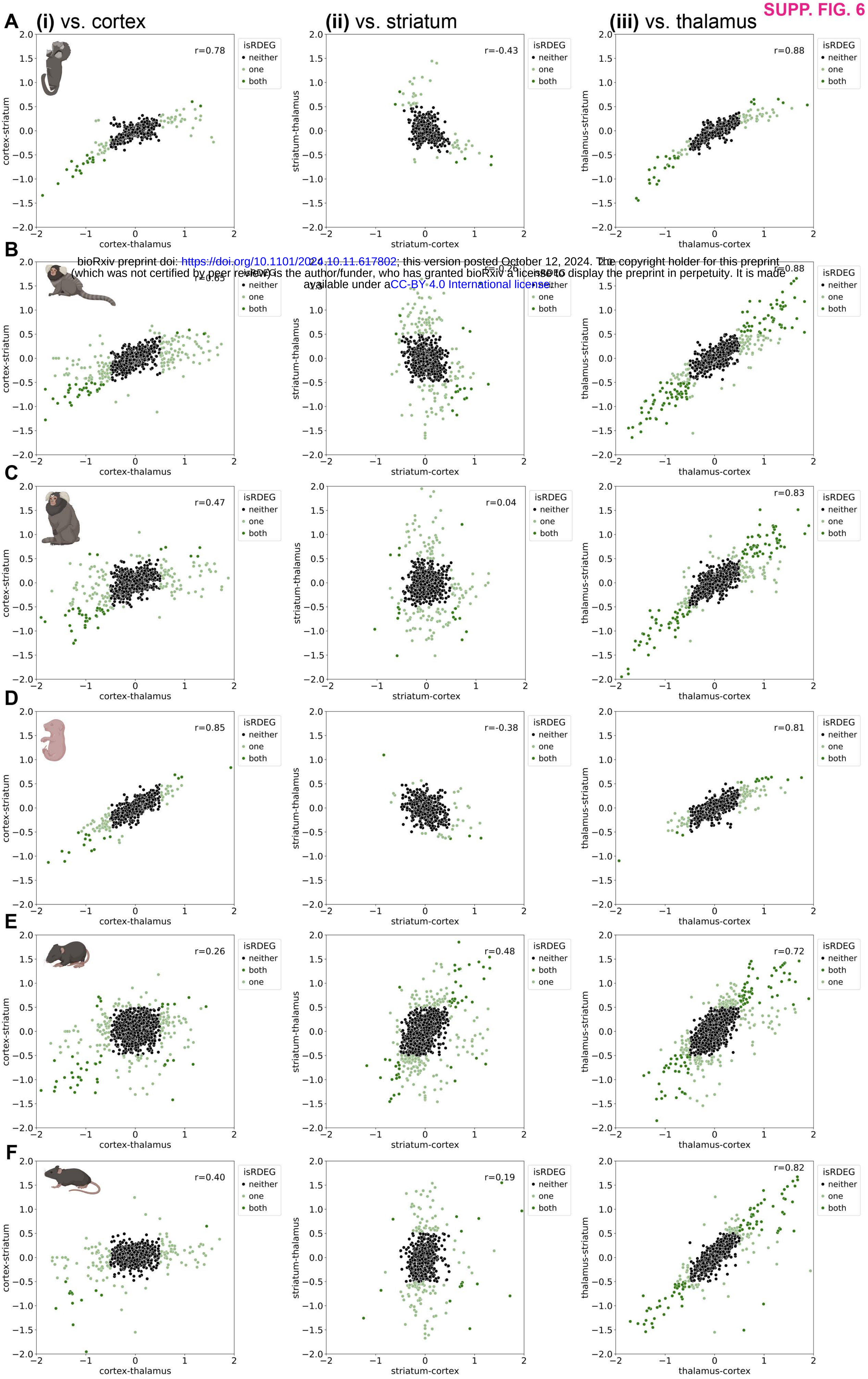

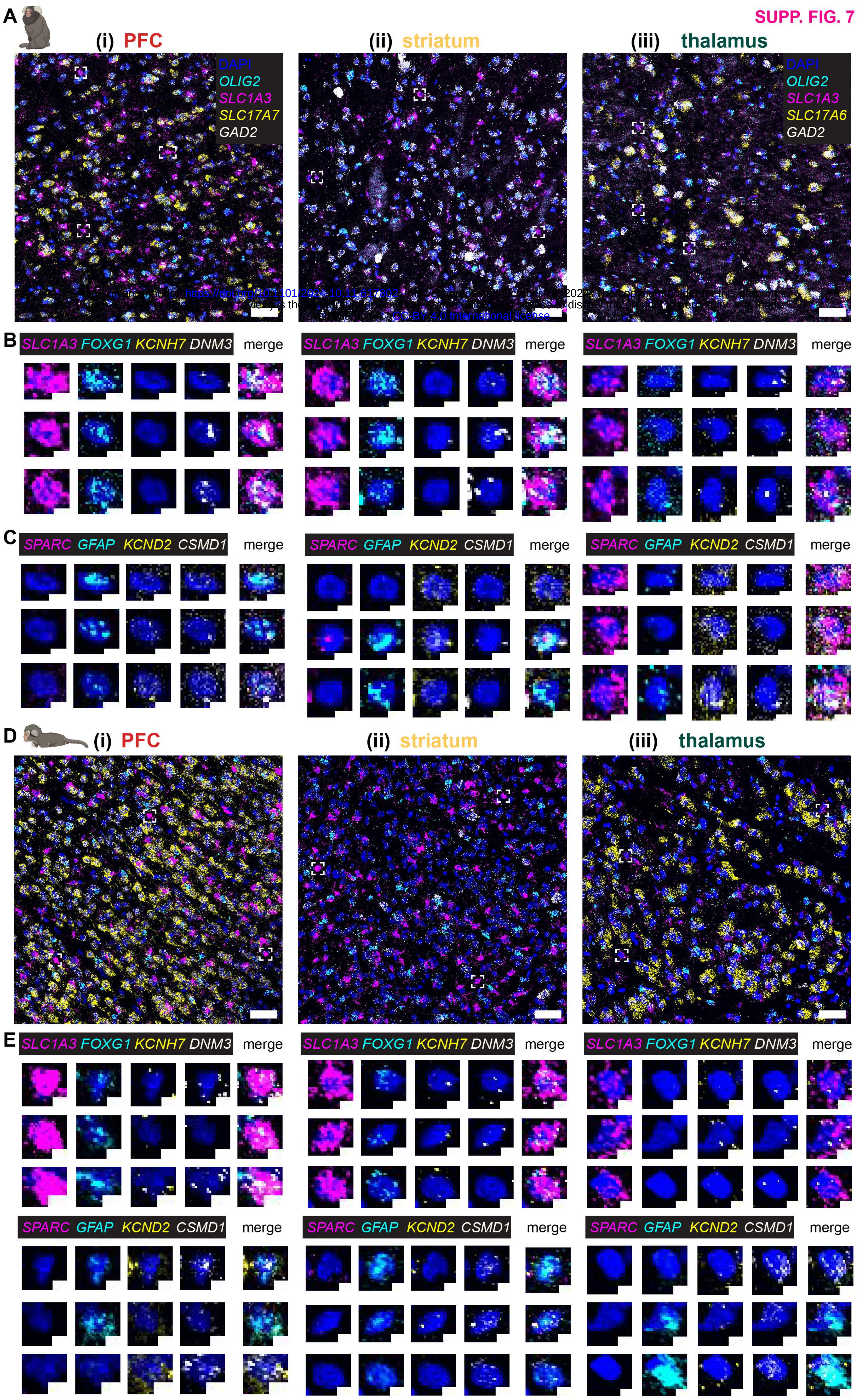

https://doi.org/10.1101/2024.10.11.617802 ... 2024. The copyright holder for this preprint ... is the author/funder, who has granted bioRxiv a license to display the preprint in perpetuity. It is made available under a CC-BY 4.0 International license.

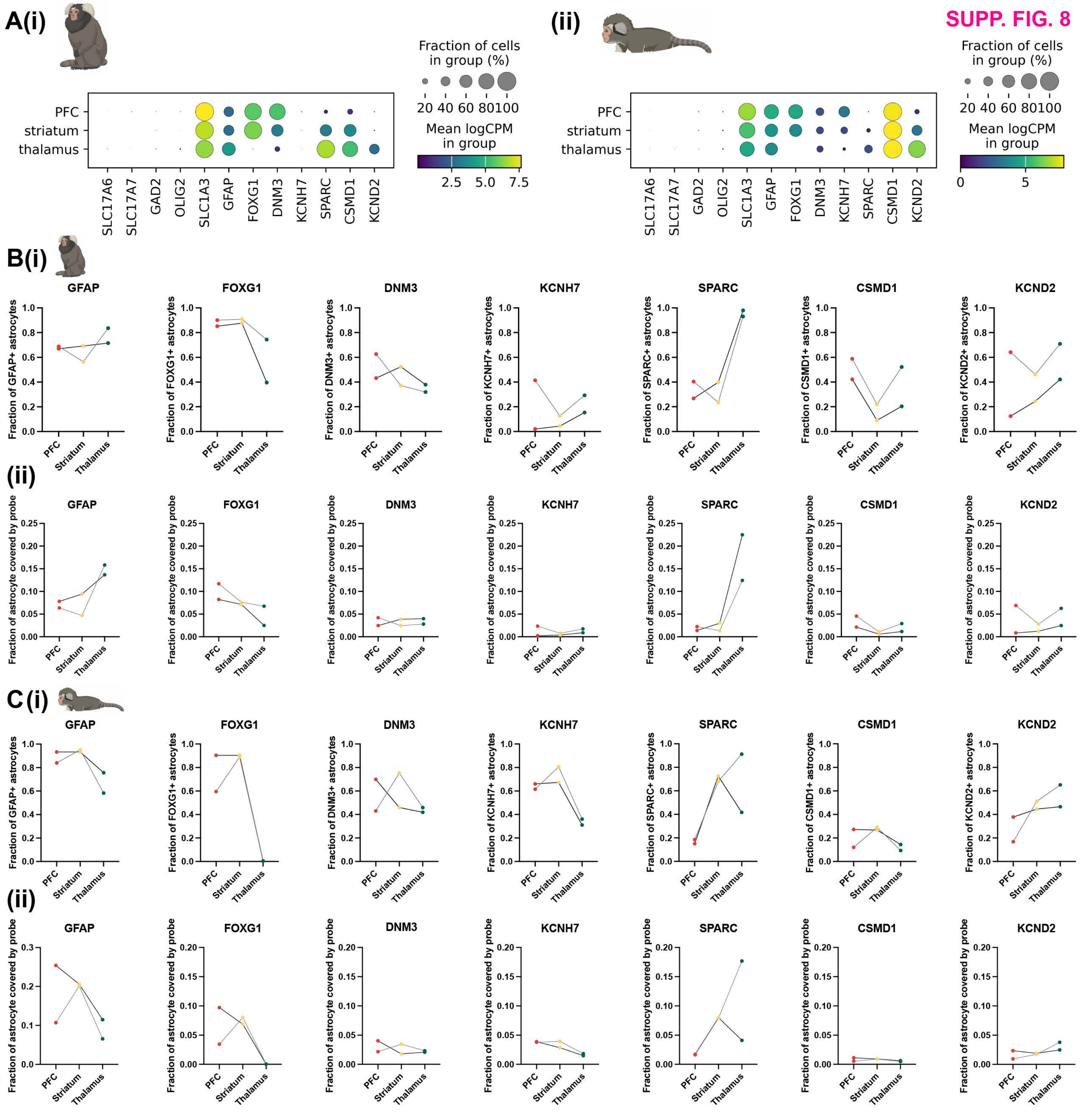
SUPP. FIG. 8
A(i)
(ii)
Fraction of cells in group (%)
20 40 60 80100
Mean logCPM in group
2.5 5.0 7.5
0 5
PFC
striatum
thalamus
SLC17A6 SLC17A7 GAD2 OLIG2 SLC1A3 GFAP FOXG1 DNM3 KCNH7 SPARC CSMD1 KCND2
B(i)
GFAP FOXG1 DNM3 KCNH7 SPARC CSMD1 KCND2
Fraction of GFAP+ astrocytes
Fraction of FOXG1+ astrocytes
Fraction of DNM3+ astrocytes
Fraction of KCNH7+ astrocytes
Fraction of SPARC+ astrocytes
Fraction of CSMD1+ astrocytes
Fraction of KCND2+ astrocytes
PFC Striatum Thalamus
(ii)
Fraction of astrocyte covered by probe
C(i)
(ii)

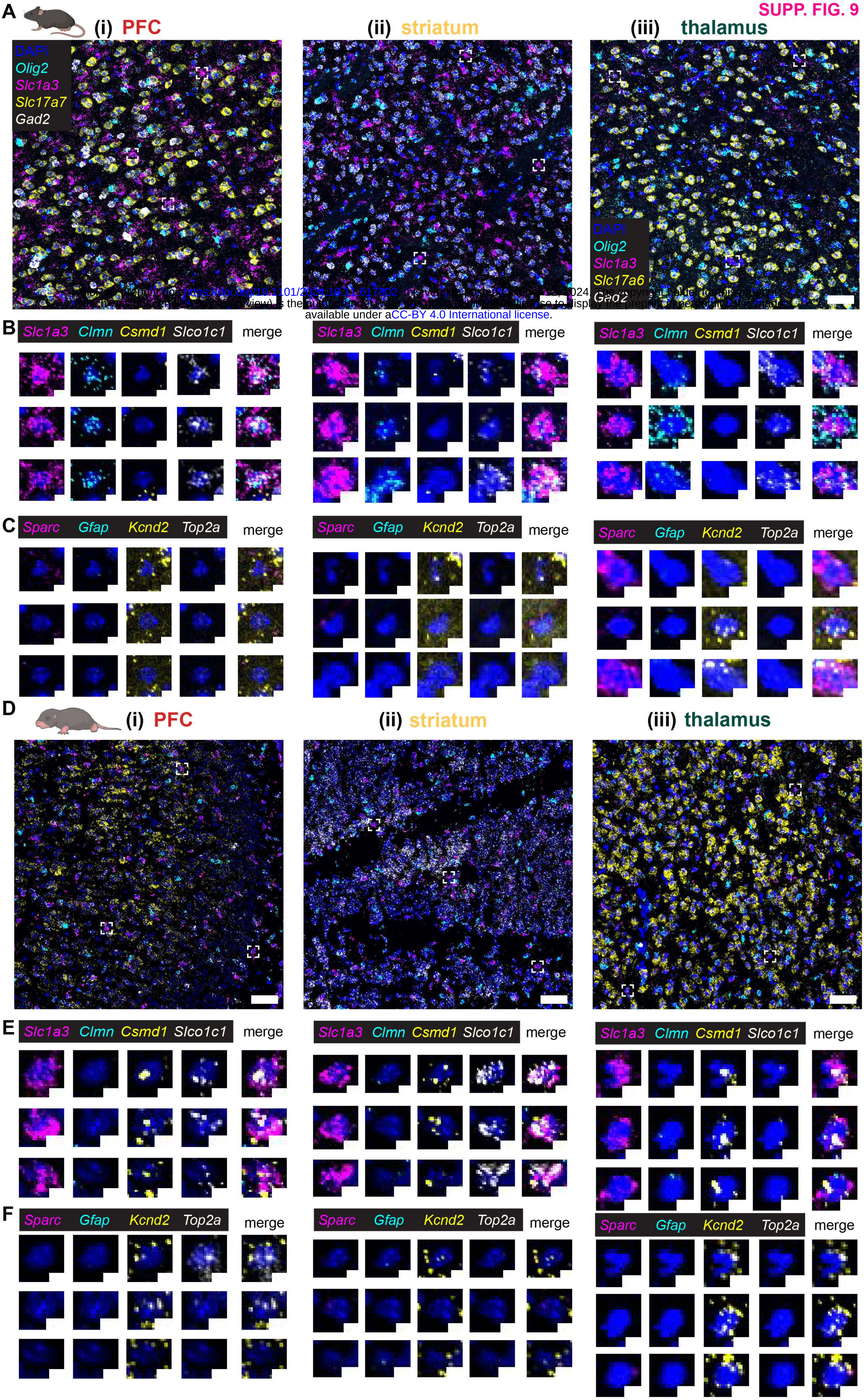

bioRxiv preprint doi: https://doi.org/10.1101/2024.10.11.617802; this version posted [illegible] 2024. The copyright holder for this preprint (which was not certified by peer review) is the author/funder, who has granted bioRxiv a license to display the preprint in perpetuity. It is made available under aCC-BY 4.0 International license.

SUPP. FIG. 10

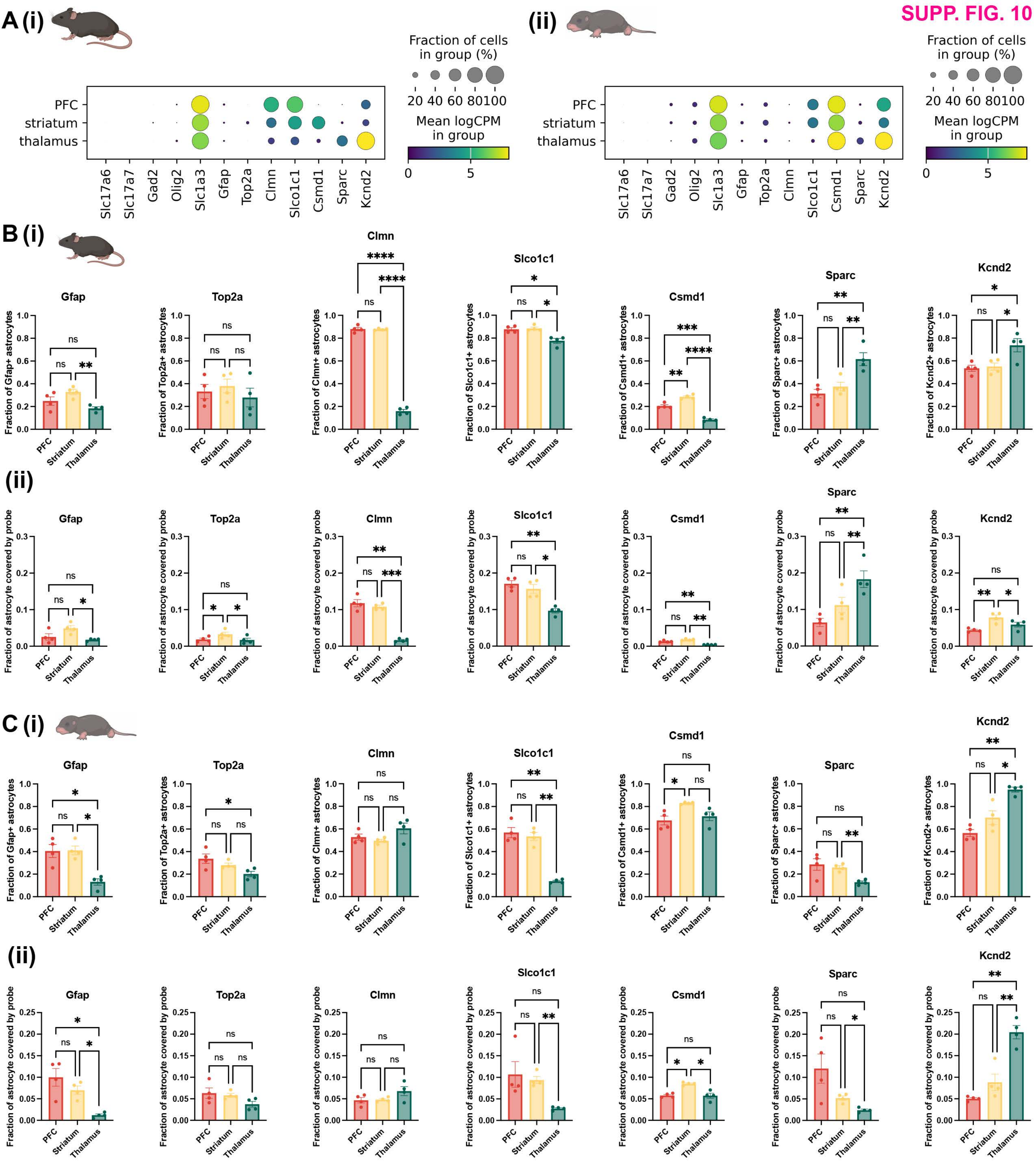
A (i)
(ii)
Fraction of cells in group (%)
20 40 60 80 100
Mean logCPM in group
0
5
PFC
striatum
thalamus
Slc17a6
Slc17a7
Gad2
Olig2
Slc1a3
Gfap
Top2a
Clmn
Slco1c1
Csmd1
Sparc
Kcnd2
B (i)
(ii)
C (i)
(ii)
Gfap
Top2a
Clmn
Slco1c1
Csmd1
Sparc
Kcnd2
Fraction of Gfap+ astrocytes
Fraction of Top2a+ astrocytes
Fraction of Clmn+ astrocytes
Fraction of Slco1c1+ astrocytes
Fraction of Csmd1+ astrocytes
Fraction of Sparc+ astrocytes
Fraction of Kcnd2+ astrocytes
Fraction of astrocyte covered by probe
PFC
Striatum
Thalamus
ns
*
**
***
****

SUPP. FIG. 11

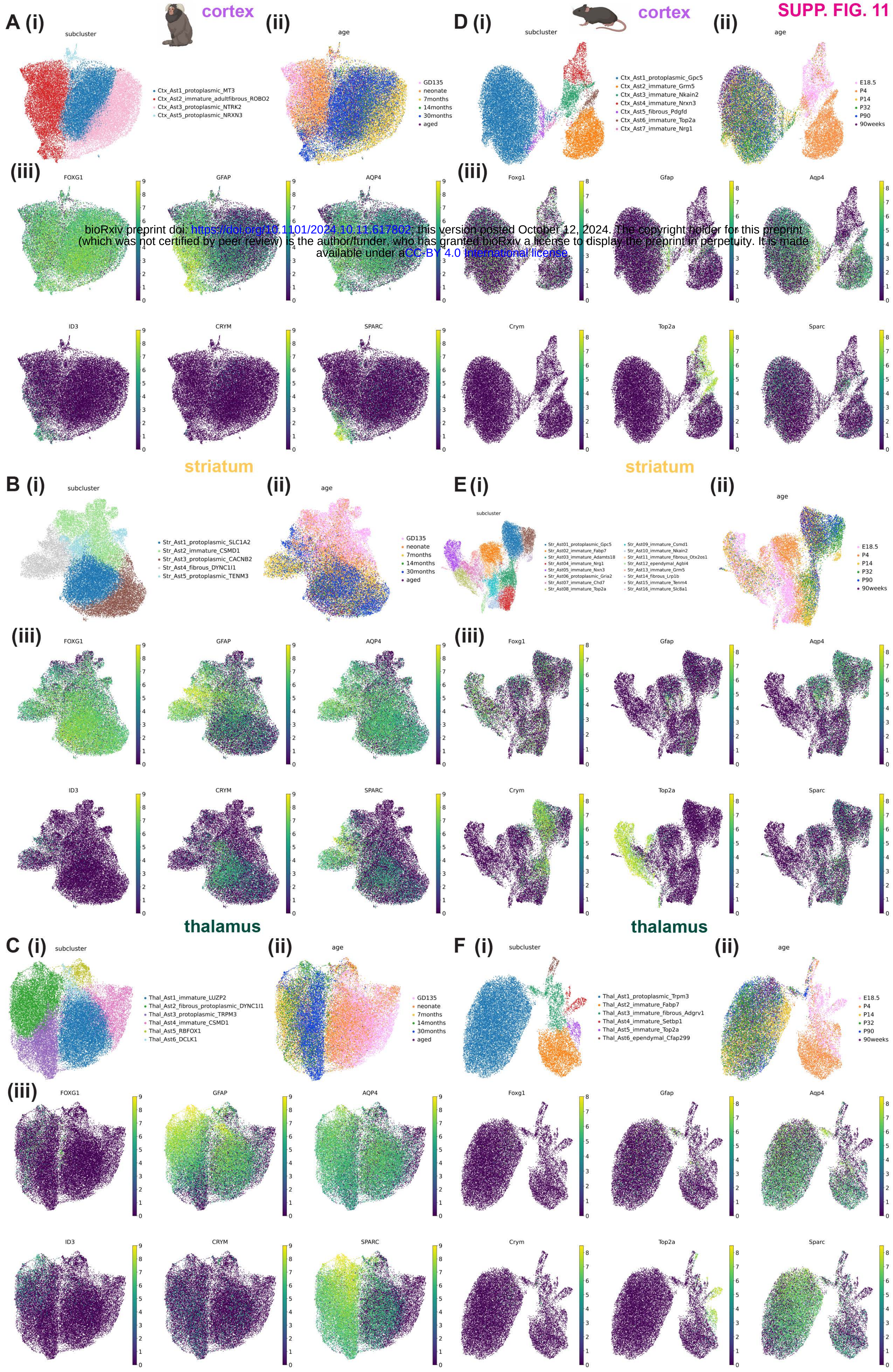

bioRxiv preprint doi: https://doi.org/10.1101/2024.10.11.617802; this version posted October 12, 2024. The copyright holder for this preprint (which was not certified by peer review) is the author/funder, who has granted bioRxiv a license to display the preprint in perpetuity. It is made available under aCC-BY 4.0 International license.

bioRxiv preprint doi: https://doi.org/10.1101/2024.10.11.617802; this version posted October 12, 2024. The copyright holder for this preprint (which was not certified by peer review) is the author/funder, who has granted bioRxiv a license to display the preprint in perpetuity. It is made available under aCC-BY 4.0 International license.

SUPP. FIG. 12

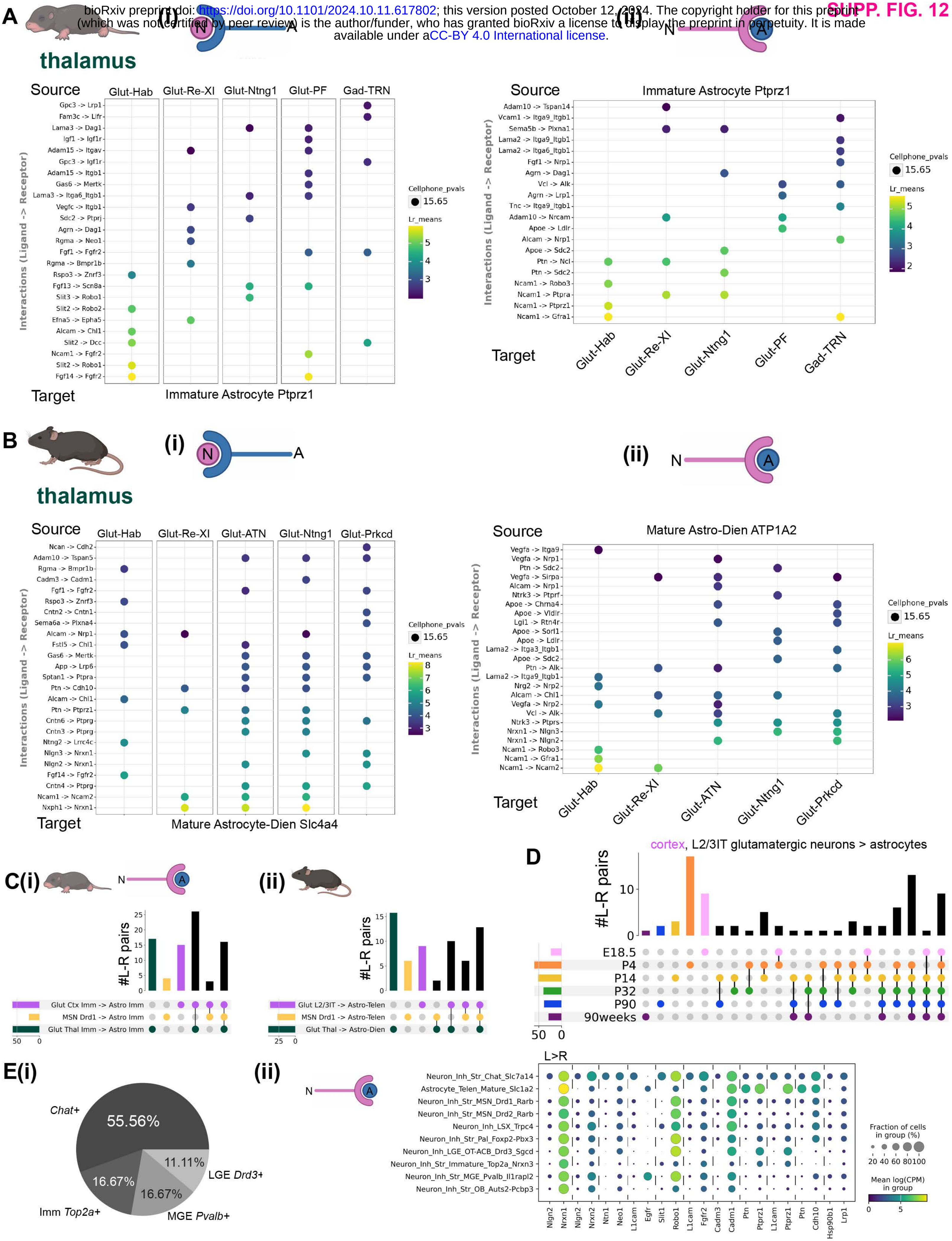

SUPP. FIG. 13

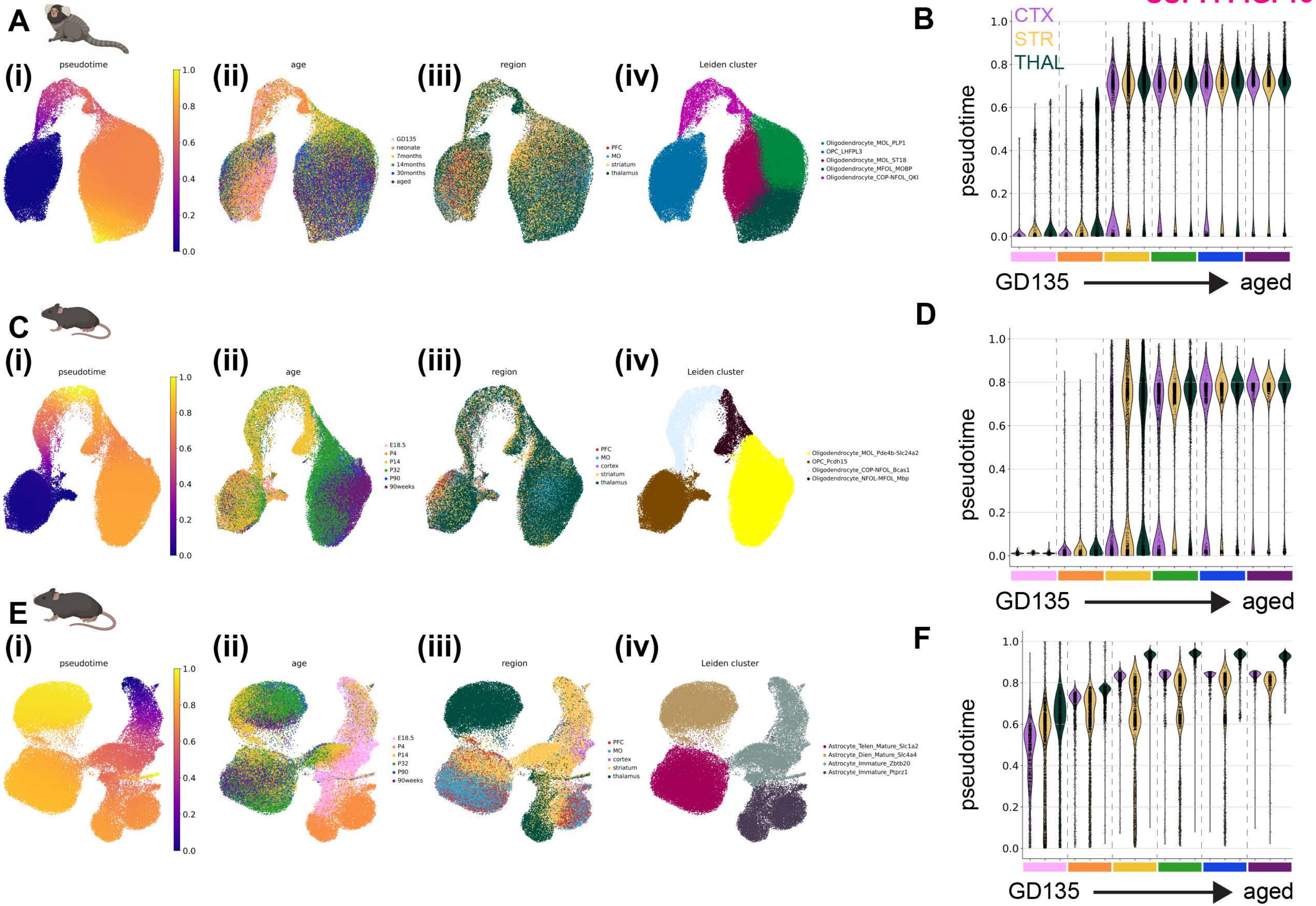
A
(i)
pseudotime
(ii)
age
GD135
neonate
7months
14months
30months
aged
(iii)
region
PFC
MO
striatum
thalamus
(iv)
Leiden cluster
Oligodendrocyte_MOL_PLP1
OPC_LHFPL3
Oligodendrocyte_MOL_ST18
Oligodendrocyte_MFOL_MOBP
Oligodendrocyte_COP-NFOL_QKI
B
CTX
STR
THAL
pseudotime
GD135
aged
C
(i)
pseudotime
(ii)
age
E18.5
P4
P14
P32
P90
90weeks
(iii)
region
PFC
MO
cortex
striatum
thalamus
(iv)
Leiden cluster
Oligodendrocyte_MOL_Pde4b-Slc24a2
OPC_Pcdh15
Oligodendrocyte_COP-NFOL_Bcas1
Oligodendrocyte_NFOL-MFOL_Mbp
D
pseudotime
GD135
aged
E
(i)
pseudotime
(ii)
age
E18.5
P4
P14
P32
P90
90weeks
(iii)
region
PFC
MO
cortex
striatum
thalamus
(iv)
Leiden cluster
Astrocyte_Telen_Mature_Slc1a2
Astrocyte_Dien_Mature_Slc4a4
Astrocyte_Immature_Zbtb20
Astrocyte_Immature_Ptprz1
F
pseudotime
GD135
aged

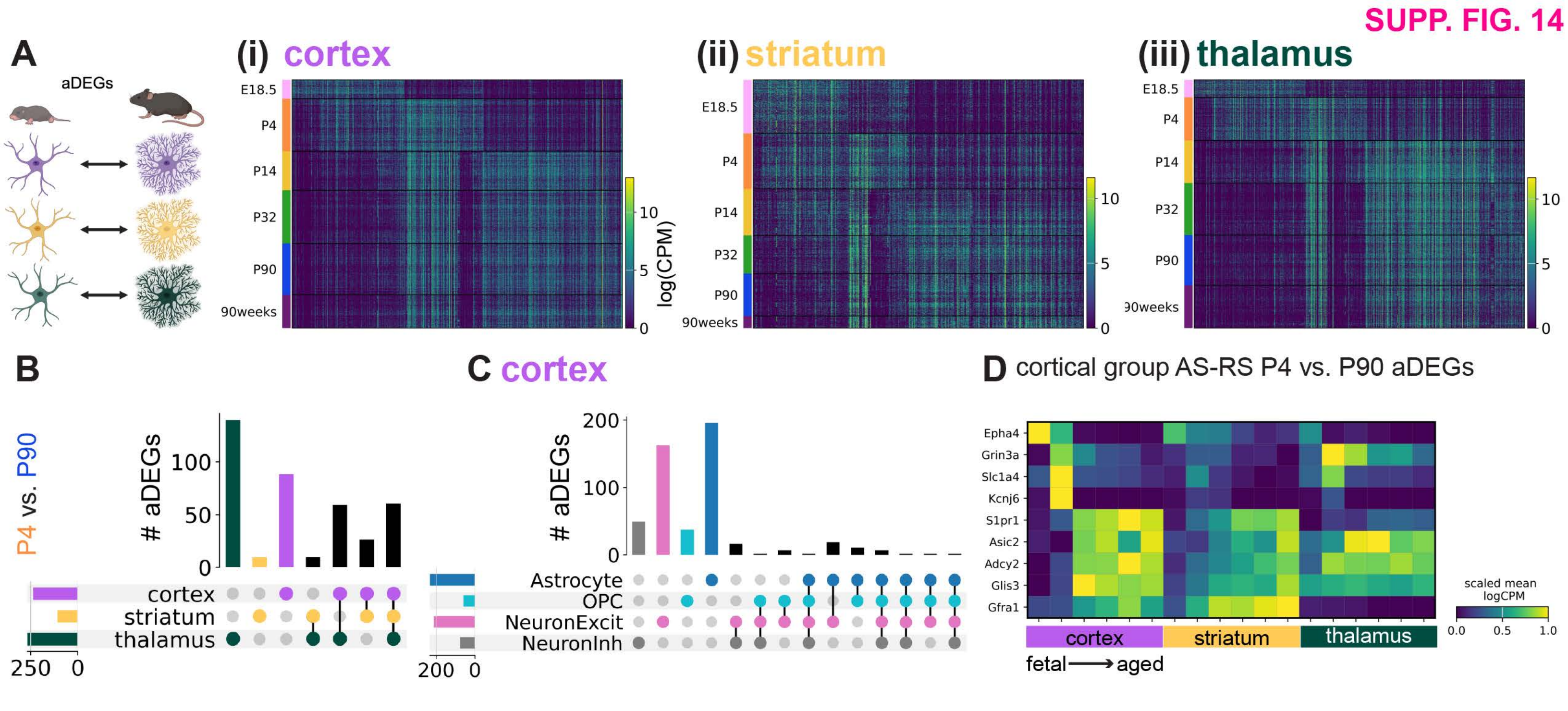
SUPP. FIG. 14
A
aDEGs
(i) cortex
(ii) striatum
(iii) thalamus
E18.5
P4
P14
P32
P90
90weeks
log(CPM)
B
P4 vs. P90
aDEGs
cortex
striatum
thalamus
C cortex
Astrocyte
OPC
NeuronExcit
NeuronInh
D cortical group AS-RS P4 vs. P90 aDEGs
Epha4
Grin3a
Slc1a4
Kcnj6
S1pr1
Asic2
Adcy2
Glis3
Gfra1
scaled mean logCPM
fetal ⟶ aged

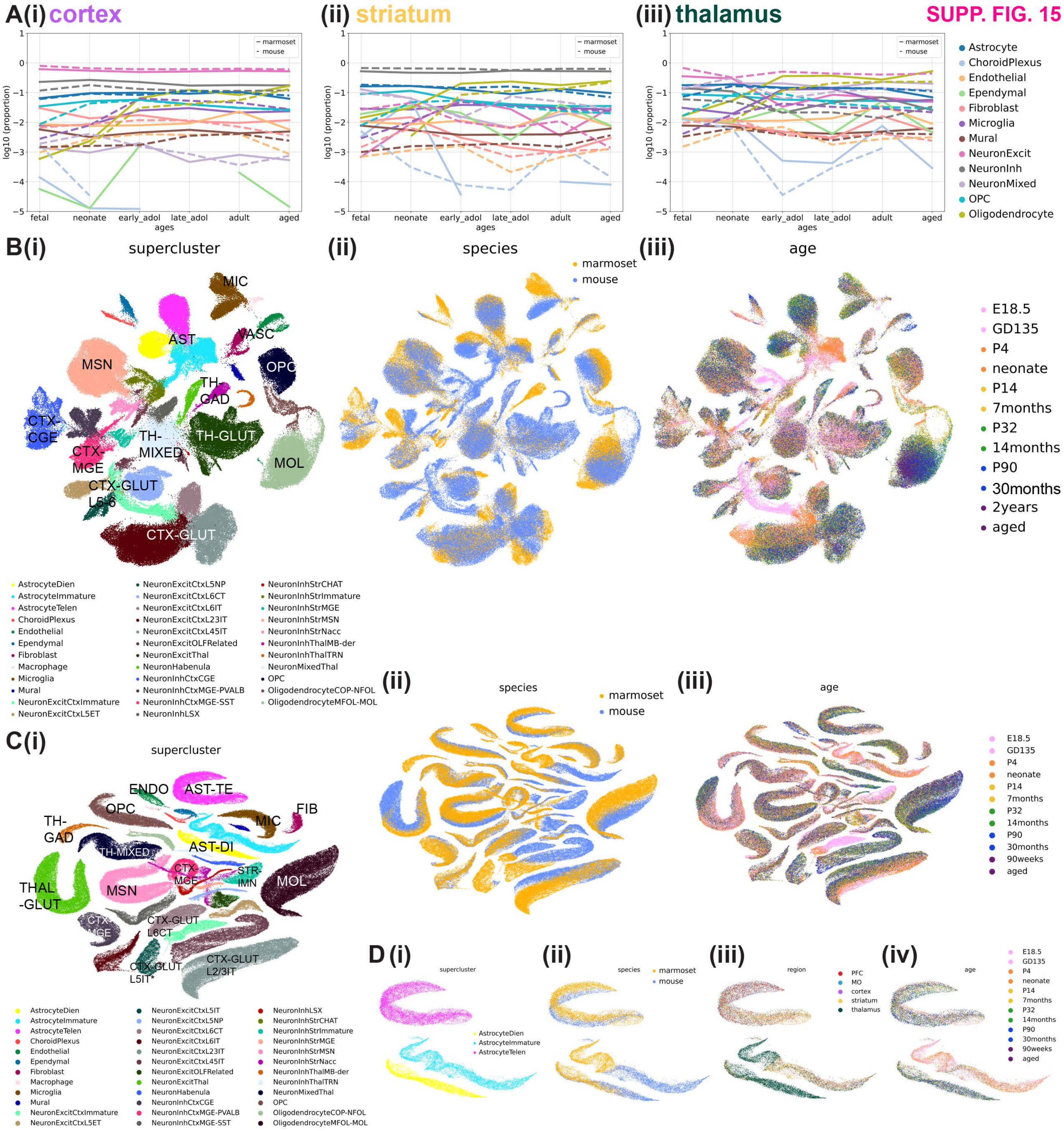
SUPP. FIG. 15
A(i) cortex
(ii) striatum
(iii) thalamus
marmoset
mouse
log10 (proportion)
fetal
neonate
early_adol
late_adol
adult
aged
ages
Astrocyte
ChoroidPlexus
Endothelial
Ependymal
Fibroblast
Microglia
Mural
NeuronExcit
NeuronInh
NeuronMixed
OPC
Oligodendrocyte
B(i) supercluster
(ii) species
(iii) age
MIC
AST
VASC
OPC
MSN
TH-GAD
CTX-CGE
TH-MIXED
TH-GLUT
CTX-MGE
MOL
CTX-GLUT L5-6
CTX-GLUT
E18.5
GD135
P4
neonate
P14
7months
P32
14months
P90
30months
2years
aged
AstrocyteDien
AstrocyteImmature
AstrocyteTelen
ChoroidPlexus
Endothelial
Ependymal
Fibroblast
Macrophage
Microglia
Mural
NeuronExcitCtxImmature
NeuronExcitCtxL5ET
NeuronExcitCtxL5NP
NeuronExcitCtxL6CT
NeuronExcitCtxL6IT
NeuronExcitCtxL23IT
NeuronExcitCtxL45IT
NeuronExcitOLFRelated
NeuronExcitThal
NeuronHabenula
NeuronInhCtxCGE
NeuronInhCtxMGE-PVALB
NeuronInhCtxMGE-SST
NeuronInhLSX
NeuronInhStrCHAT
NeuronInhStrImmature
NeuronInhStrMGE
NeuronInhStrMSN
NeuronInhStrNacc
NeuronInhThalMB-der
NeuronInhThalTRN
NeuronMixedThal
OPC
OligodendrocyteCOP-NFOL
OligodendrocyteMFOL-MOL
C(i) supercluster
(ii) species
(iii) age
ENDO
AST-TE
OPC
FIB
TH-GAD
MIC
TH-MIXED
AST-DI
THAL-GLUT
MSN
CTX-MGE
STR-IMN
MOL
CTX-CGE
CTX-GLUT L6CT
CTX-GLUT L5IT*
CTX-GLUT L2/3IT
90weeks
NeuronExcitCtxL5IT
D (i) supercluster
(ii) species
(iii) region
(iv) age
PFC
MO
cortex
striatum
thalamus

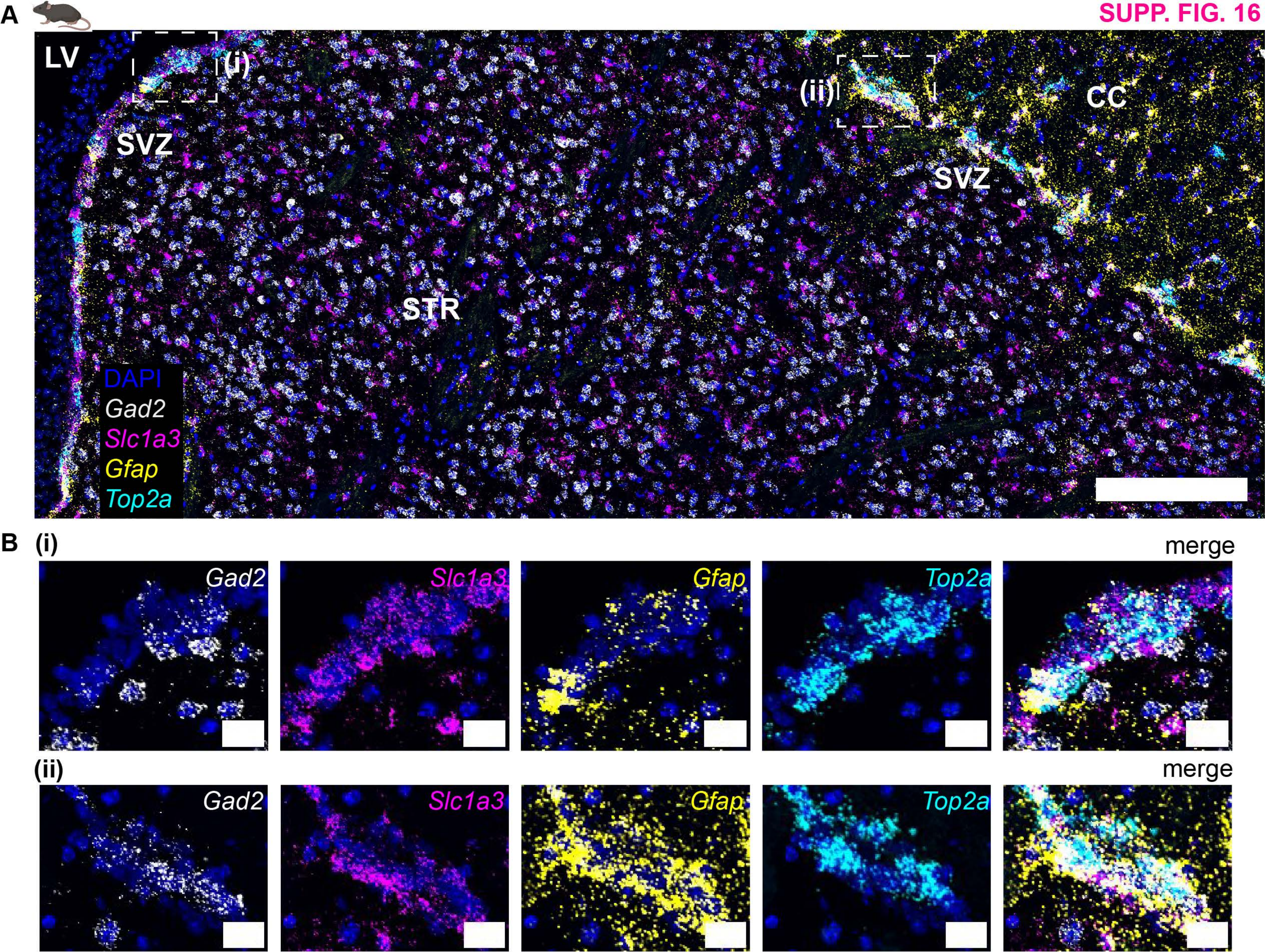
A
LV
(i)
(ii)
CC
SVZ
SVZ
STR
DAPI
Gad2
Slc1a3
Gfap
Top2a
B
(i)
merge
Gad2
Slc1a3
Gfap
Top2a
(ii)
merge
Gad2
Slc1a3
Gfap
Top2a

(which was not certified by peer review) is the author/funder, who has granted bioRxiv a license to display the preprint in perpetuity. It is made available under aCC-BY 4.0 International license.

SUPP. FIG. 17

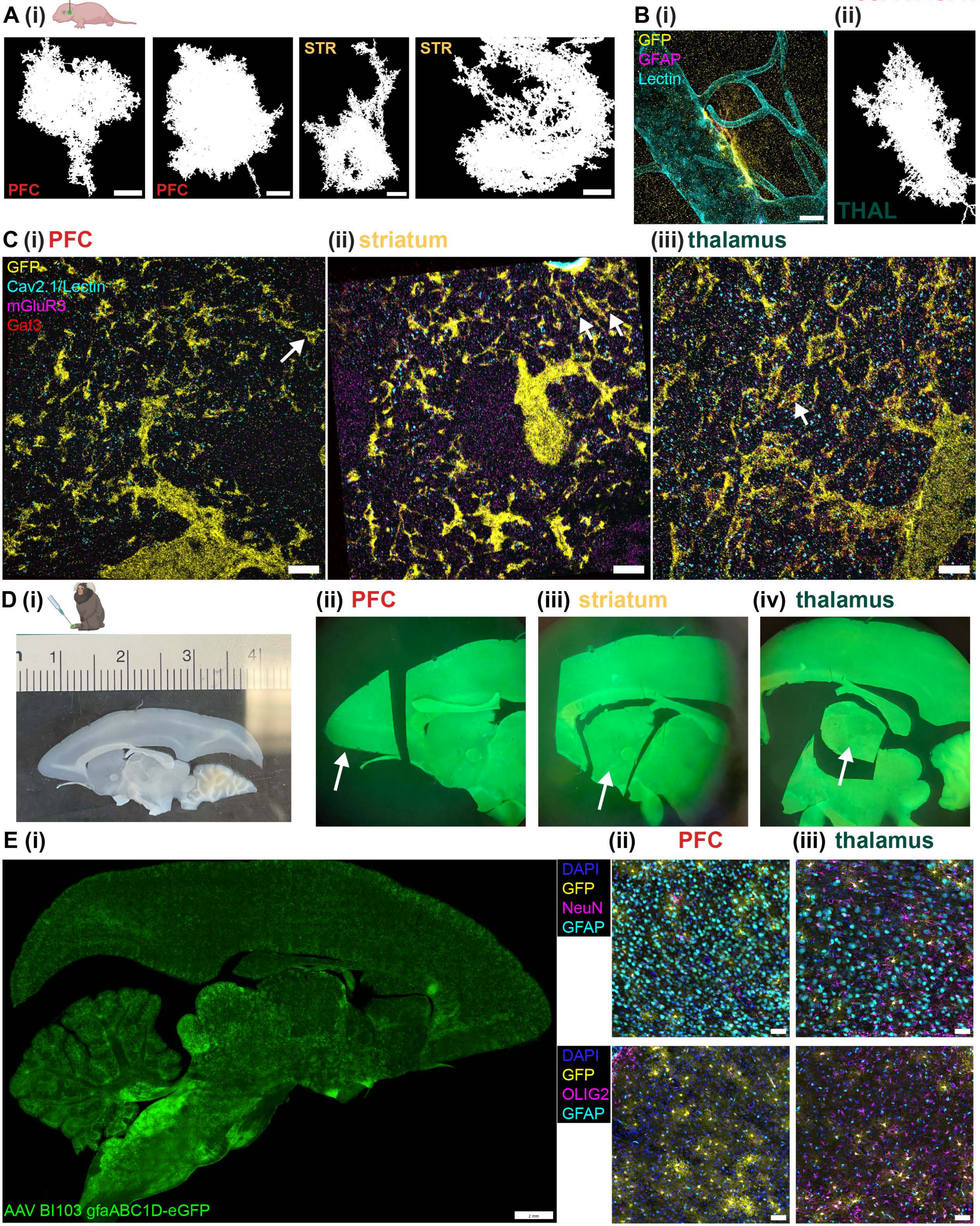

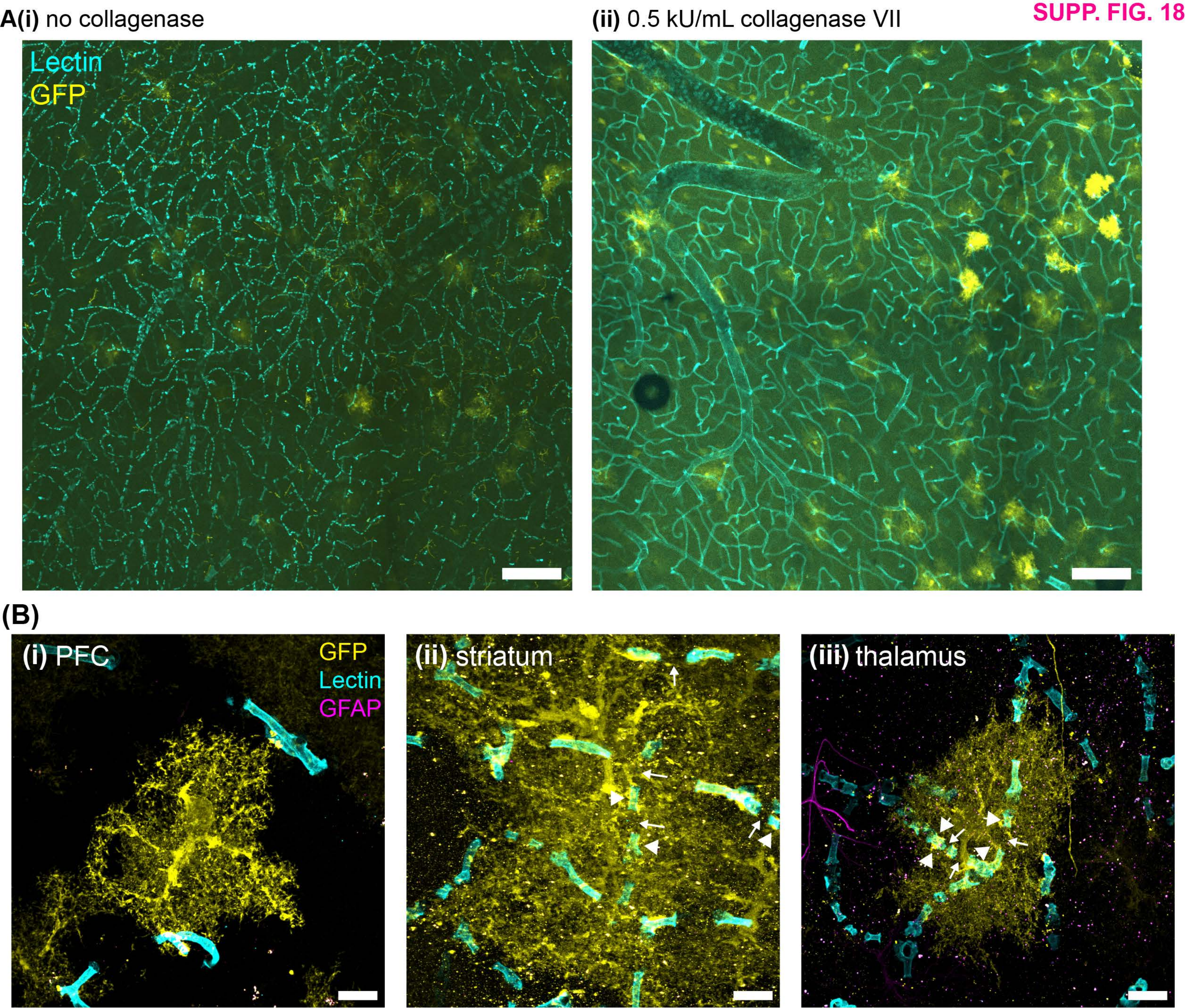
SUPP. FIG. 18
A(i) no collagenase
(ii) 0.5 kU/mL collagenase VII
Lectin
GFP
(B)
(i) PFC
GFP
Lectin
GFAP
(ii) striatum
(iii) thalamus

bioRxiv preprint doi: https://doi.org/10.1101/2024.10.11.617802; this version posted October 12, 2024. The copyright holder for this preprint (which was not certified by peer review) is the author/funder, who has granted bioRxiv a license to display the preprint in perpetuity. It is made available under aCC-BY 4.0 International license.

Supplementary Note 1

The reader will note that not all marmoset astrocyte rDEGs followed the predicted expression pattern by RNAscope HiPlex FISH, and that there is a high degree of variability between the two replicates (**Fig. S8**). For example, *FOXG1* expression was spuriously detected in adult marmoset thalamus (at lower levels than PFC and striatum) by our CellProfiler pipeline, perhaps due to low and variable signal-to-noise. Nevertheless, *FOXG1* expression appears negligible in thalamic astrocytes when compared to PFC and striatum in our higher-quality marmoset sample, highlighting the limitations of a thresholding-based quantification approach (**Fig. S7**). In another example, the fraction of astrocytes positive for *KCND2* differs by several fold in the PFC between the two adult marmoset replicates. Our inability to quantitatively validate rDEG patterns in some replicates is likely due to variable tissue quality (even between regions, with striatum usually exhibiting less autofluorescence than PFC or thalamus), sampling a different position on the medial/lateral axis (especially for neonate marmoset samples), decreased signal-to-noise in later rounds of the RNAscope HiPlex protocol, overlapping RNA signal from adjacent cells of other cell types, or a combination of these factors. We found the RNAscope HiPlex protocol to produce variable results in marmoset tissue, especially in later rounds, even after several attempts at optimization. In mouse, our quantification suggested *Gfap* expression was higher at both time points than predicted by snRNAseq, as was *Sparc* expression in neonates (**Fig. S10C**), findings which were not readily apparent by visual inspection of the images (**Fig. S9**). This discrepancy may reflect intra-regional variability in rDEG expression or over-sensitivity of the CellProfiler pipeline. In some cases, discrepancies between snRNAseq expression and FISH signal may be due to distinct gene capture strategies, or the use of probe target sequences on exons not highly expressed in astrocytes. Nevertheless, taken together, our multiplexed FISH results confirm the divergent gene expression patterns of telencephalic and diencephalic astrocytes, with notable changes occurring over postnatal development.
